# Endogenous Glandular Chemistry and Methyl Eugenol–derived Metabolites in the Pheromone Communication of *Bactrocera umbrosa*

**DOI:** 10.1007/s10886-026-01726-2

**Published:** 2026-06-13

**Authors:** Tatsuya Kiuchi, Suk-Ling Wee, Stefan Schulz

**Affiliations:** 1 Institute of Organic Chemistry, Technische Universität Braunschweig, Hagenring 30, 38106 Braunschweig, Germany; 2 Centre for Insect Systematics, Department of Biological Sciences and Biotechnology, Faculty of Science and Technology, Universiti Kebangsaan Malaysia, Bangi, 43600 Selangor Darul Ehsan Malaysia

**Keywords:** Tephritidae, *Bactrocera umbrosa*, GC–MS, Phenylpropanoids, Pharmacophagy, Sexual attraction, Pheromone communication

## Abstract

*Bactrocera umbrosa* (Fabricius) is an oligophagous fruit fly of economic importance that infests mainly *Artocarpus* fruits (Moraceae). The sexual communication system of this species is poorly understood, partly due to difficulties in establishing laboratory colonies. Males of *B. umbrosa* are strongly attracted to and voraciously fed on methyl eugenol (ME), a naturally occurring plant secondary compound. Here, we elucidate the endogenously produced and ME–derived chemical signals involved in the species’ communication. Gas chromatography–mass spectrometry analyses of pheromonal glands revealed that immature males produced four endogenous compounds, whereas sexually mature males produced six additional volatile compounds, including the terpenoids (*R*)-6-methyl-2-vinylhept-5-ene-1,2-diol and (*S*)-7-methyl-3-methyleneoct-6-ene-1,2-diol, which, to our knowledge, have not previously been documented in insects. Following ME ingestion, males accumulated six ME metabolites—eugenol, isoeugenol, (*S*)-1ʹ-hydroxymethyleugenol ((*S*)-1ʹ-HME), 2-allyl-4,5-dimethoxyphenol (DMP), (*Z*)-3,4-dimethoxycinnamyl alcohol (Z-DMC), and (*E*)-coniferyl alcohol (E-CF). Behavioral assays using a Y-tube olfactometer demonstrated that two endogenous compounds (3-ethyl-2,5-dimethylpyrazine and 6-oxo-1-nonanol) and two ME metabolites (DMP and E-CF) significantly attracted virgin females during courtship. In contrast, none of the endogenous volatiles was attractive to the males, whereas three ME metabolites—eugenol, isoeugenol, and DMP—elicited significant male attraction during dusk. These findings demonstrated that *B. umbrosa* utilizes endogenous compounds as sex-specific signals to attract conspecific females, while distinct ME-derived metabolites mediate both male-male and male-female communication, facilitating male aggregation and enhancing sexual attraction. These insights contribute to a deeper understanding of the intricate glandular chemistry and chemical communication system of the *Artocarpus* fruit fly, which may inform future strategies for its control and management.

**Supplementary Information:**

The online version contains supplementary material available at 10.1007/s10886-026-01726-2.

## Introduction

*Bactrocera* is a genus of tephritid flies comprising nearly 500 species, some of which are major pests of a wide variety of fruit and vegetable crops (Drew 1989; Vargas et al. 2015). In most *Bactrocera* species, volatile organic compounds released from the male rectal glands during the courtship period play key roles in intra- and inter-specific communication (Fletcher 1969; Nishida et al. 1988a).

The *Artocarpus* fruit fly, *Bactrocera umbrosa* (Fabricius) (Diptera: Tephritidae), is an oligophagous tephritid that infests fruits of the Moraceae family, including jackfruit (*Artocarpus heterophyllus* Lam.), chempedak (*A. integer* (Thunberg)), and breadfruit (*A. altilis* (Parkinson)) (Allwood et al. 1999; Lauciello et al. 2024). The species occurs across a wide geographic range extending from the tropical South Pacific (New Caledonia, Papua New Guinea, Solomon Islands, Vanuatu) westwards into Southeast Asia (the Philippines, Indonesia, Malaysia, and southern Thailand), overlapping with the distributions of *B. dorsalis* and *B. carambolae* (Clarke et al. 2005; Hardy 1973; Krosch et al. 2019). Although *B. umbrosa* has long been recognized as an economically important pest (Drew et al. 1982), research progress has been constrained by the challenges of establishing laboratory colonies and by the seasonality of some *Artocarpus* hosts (Walker et al. 1997; Clarke et al. 2001; Lauciello et al. 2024).

Chemical communication in the *Artocarpus* fruit fly is understudied. Previous chemical analyses of male rectal glands of *B. umbrosa* identified several major endogenous constituents, including 6-oxo-1-nonanol (Perkins et al. 1990). However, their roles in the pheromonal communication of *B. umbrosa* remain largely unexplored, although 6-oxo-1-nonanol is also produced endogenously by *B. carambolae* males and elicited attraction response in conspecific females (Wee and Tan 2005a), in addition to functioning as an allomone against predation (Wee and Tan 2005b).

Methyl eugenol (ME) is a naturally occurring phenylpropanoid widely distributed in the plant kingdom, being reported from more than 450 species in 80 families and 38 orders (Tan and Nishida 2012). Since its serendipitous discovery as the active attractant for certain male *Bactrocera* species in citronella oil (Howlett 1915), ME has been extensively used in species detection, monitoring, and male annihilation programs (Steiner et al. 1965; Vargas et al. 2010a, b). Once ingested, ME is metabolized into ME analogues that are released primarily during the mating period at dusk to enhance male mating competitiveness (Nishida et al. 1988a; Tan et al. 2011). After consuming ME, males of the invasive oriental fruit fly, *B. dorsalis* (Hendel), produce (*E*)-coniferyl alcohol (E-CF) and 2-allyl-4,5-dimethoxyphenol (DMP), which were shown to function as sex pheromone and as allomone against vertebrate predators (Nishida et al. 1988a; Tan and Nishida 1996, 1998; Khoo and Tan 2005; Nishida and Fukami 1990; Jakubas et al. 1992; Wee and Tan 2001). Conversely, in the carambola fruit fly, *B. carambolae* Drew and Hancock, ME is only converted into E-CF with a delayed mating enhancement when compared to its sibling species, *B. dorsalis* (Tan and Nishida 1996; Wee and Tan 2005a, 2007a). This shows that ME-mediated sexual communication is unique to species, including closely related cryptic species.

Males of *B. umbrosa* are strongly attracted to and voraciously feed on ME (Tan 1985; Tan and Jaal 1986; Tan and Nishida 2012). ME consumption by *B. umbrosa* has been found to enhance intraspecific sexual communication and male mating success (Wee et al. 2018). Preliminary chemical analyses on the rectal glands of ME-fed *B. umbrosa* males have found several ME-derived metabolites, including DMP and (*Z*)-3,4-dimethoxycinnamyl alcohol (Z-DMC) (Tan 2010); however, it remains unclear if these compounds are involved in the sexual communication of the species. Here, we further elucidate the endogenous and ME-derived compounds present in the rectal gland of *B. umbrosa* males and assess their behavioral activities, thereby advancing our understanding of the species’ chemical communication system.

## Materials and Methods

### Insects

Mature larvae of *B. umbrosa* were collected from naturally infested jackfruits and chempedak at a local organic plantation in Dengkil, Selangor, Malaysia (2°53’02.5"N, 101°45’47.9"E). The emerged adults were sex-segregated within four days after emergence to ensure virginity. Male and female flies were maintained separately in screened cages and provided with a sugar-yeast hydrolysate diet (3:1, w/w) along with water *ad libitum*. The insectary was maintained at 25 ± 2 °C and 75 ± 10% relative humidity under fluorescent illumination with a 12:12 h light: dark photoperiod, supplemented with natural daylight from windows.

### Chemicals

Methyl eugenol (ME) (> 99.8% purity) was purchased from Agrisense-BCS Ltd. Chemical standards: 3-Methyl-1-butanol (**1**, > 99% purity) was purchased from Sigma-Aldrich, 3-methyl-2-buten-1-ol (**2**, > 98% purity) from TCI, and eugenol (**10**, 98% purity) and isoeugenol (**11**, > 99% purity) from BLD Pharmatech Ltd. Additional standards, (2*S*,5*SR*)-2-methyl-1,6-dioxaspiro[4.5]decanes (**3**), 3-ethyl-2,5-dimethylpyrazine (EDMP, **4**), 1,7-dioxaspiro[5.5]undecane (**5**), *N*-(3-methylbutyl)acetamide (**6**), (*S*)-6-methyl-2-vinylhept-5-ene-1,2-diol ((*S*)-**7**), 6-oxo-1-nonanol (**8**), (*S*)-7-methyl-3-methyleneoct-6-ene-1,2-diol ((*S*)-**9**), (*S*)-1ʹ-hydroxymethyleugenol ((*S*)-1ʹ-HME, (*S*)-**12**), 2-allyl-4,5-dimethoxyphenol (DMP, **13**), (*Z*)-3,4-dimethoxycinnamyl alcohol (Z-DMC, **14**) and (*E*)-coniferyl alcohol (E-CF, **15**) were synthesized in-house following standard organic synthesis protocols (see Supplementary information for details). For bioassays, all compounds listed above were used except for (*S*)-1ʹ-HME ((*S*)-**12**), whose structure was elucidated only after completion of the behavioral experiments in Malaysia. Chiral diol compounds were tested as racemates, and the spiroacetals were used as mixtures of stereoisomers at the spiro center. All products were purified (> 98% purity by GC–MS), and their structures were confirmed by NMR analyses.

### Extraction of Rectal Glands for Chemical Analysis

- Non-manipulated male flies

Male flies at two developmental stages—immature (6 days after emergence, DAE) and sexually mature (20 DAE) (Wee et al. 2018)—were lightly cold-immobilized at − 5 °C for 5 min. The rectal gland was carefully removed from the body by grasping its aedeagus and snipping it with fine forceps. Each isolated gland was extracted with 0.50 mL of absolute ethanol (> 99.8% purity Sigma-Aldrich).

(b) ME-fed male flies

During the morning hours (08:00–11:00 h), when *B. umbrosa* males exhibit strong responsiveness to ME (Wee et al. 2018), 1.0 µL of undiluted ME was applied to a 9.0 cm diameter Whatman No. 1 filter paper using a Hamilton glass syringe. The treated filter paper was placed in a disposable Petri dish and used as the ME source. A single male fly was introduced and allowed to feed on the ME for 15 min. This procedure was repeated until 10 males had been fed. The ME-fed flies were then placed in a cylindrical mesh cage (7 cm diameter × 15 cm height) supplied with food and water *ad libitum*. During the dusk mating period of the following day, the rectal glands of ME-fed males were extracted with 0.50 mL absolute ethanol.

### Gas Chromatography-mass Spectrometry and Chemical Analysis

GC–MS analyses were performed with a combination of an Agilent Technologies 8860 gas chromatograph connected to an Agilent Technologies 5977B Series MSD. Mass spectrometry was performed in electron ionization (EI) mode at 70 eV. An HP-5 MS column (Agilent Technologies, 30 m length, 0.25 mm diameter, 0.25 μm film thickness, 350 °C) with helium (Westfalen AG, 99.999%) at a flow rate of 1.2 mL/min as the carrier gas was used. The temperature program started at 50 °C, held for 5 min, then increased at 5 °C/min to 320 °C. Linear retention indices were determined from a linear, homologous series of *n*-alkanes. Volatile organic compounds were identified using AMDIS software (version 2.73) by comparing both their mass spectra and GC retention indices (RI) with reference data from NIST23 and MACE databases (Schulz and Möllerke 2022). For selected unknown compounds, GC–IR analysis and derivatization techniques were also employed. Compound identifications were further verified by comparison with authentic standards, either commercially obtained or synthesized in-house. For chiral compounds, enantiomeric compositions were determined by chiral GC using HYDRODEX β-6TBDM (Macherey & Nagel, 30 m × 0.25 mm i.d., film thickness 0.40 μm) and Beta-Dex™ 225 (SUPELCO, 30 m × 0.25 mm i.d., film thickness 0.25 μm) chiral stationary phase columns (Fig. S19, S20, and S25). Quantification was performed using calibration curves generated from authentic standards, with internal standard 1-dodecanol (Sigma-Aldrich) used for correction (Fig. S21–S24).

### Y-olfactometer Bioassay

The dual-choice tests to evaluate the behavioral response of sexually mature virgin females (22–33 DAE) and males (19–30 DAE) of *B. umbrosa* towards the endogenous compounds and ME metabolites was carried out in a Y-tube olfactometer. The Y-olfactometer (2-cm diameter) consisted of a main glass tube (13 cm long) with two arms (13 cm long) positioned at a 30° angle. Each arm, connected to a glass adaptor (9 cm long, 2 cm diameter), was used to contain the odor source.

All test chemicals were diluted with absolute ethanol to 1 µg/µL, and 2 µL of solution was applied to a piece of filter paper (1 × 1 cm; Whatman No. 1). After allowing the solvent to evaporate for 30 s, the treated filter paper was then placed into one of the glass adaptors as treatment while another filter paper treated with 2 µL of absolute ethanol was placed into the other glass adaptor as control. Charcoal-purified and humidified air (15 mL/min) was passed through both arms to deliver the stimulus to the test insects. A fly was introduced into the main arm of the Y-tube using a clean glass vial and observed for 10 min. A positive response was defined as the fly moving upward to one of the arms of the Y-olfactometer (i.e., either the treatment or control) and remaining there for at least 30 s. If a fly stayed in the main arm and did not make a choice within 10 min, the data were recorded as “no choice/response”, and the fly was discarded. After every five replicates, the olfactometer was rinsed with absolute ethanol and air-dried. After every set of replicates, the positions of the arms were alternated to avoid positional bias. Each fly was tested only once, and a total of 30 responsive replicates were obtained for each compound. The Y-tube olfactometer was positioned near a window to receive illumination from natural sunlight, with all artificial lights turned off. All experiments were conducted during dusk between 17:30 and 19:15, coinciding with the courtship period in this species (Wee et al. 2018), in an environmentally controlled room (27 ± 2 °C, 75 ± 10% relative humidity). Only data with positive responses were used in the statistical analysis.

### Statistical Analysis

Yates’ corrected Chi-square test was used to evaluate the differences between the number of *B. umbrosa* males and females entering each arm of the Y-tube olfactometer.

## Results

**Fig. 1:**
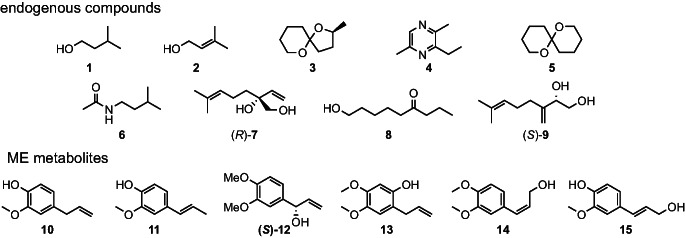
Structures of compounds identified from rectal glands of mature male *B. umbrosa*: 3-methyl-1-butanol (**1**), 3-methyl-2-buten-1-ol (**2**), (2*S*,5*SR*)-2-methyl-1,6-dioxaspiro[4.5]decane (**3**), 3-ethyl-2,5-dimethylpyrazine (EDMP, **4**), 1,7-dioxaspiro[5.5]undecane (**5**), *N*-(3-methylbutyl)acetamide (**6**), (*R*)-6-methyl-2-vinylhept-5-ene-1,2-diol ((*R*)-**7**), 6-oxo-1-nonanol (**8**), (*S*)-7-methyl-3-methyleneoct-6-ene-1,2-diol ((*S*)-**9**), eugenol (**10**), isoeugenol (**11**), (*S*)-1ʹ-hydroxymethyleugenol ((*S*)-1ʹ-HME, (*S*)-**12**), 2-allyl-4,5-dimethoxyphenol (DMP, **13**), (*Z*)-3,4-dimethoxycinnamyl alcohol (Z-DMC, **14**), and (*E*)-coniferyl alcohol (E-CF, **15**)

The chemical structures and compound numbering of all compounds identified in this study are shown in Fig. 1.

### Identification of Endogenous Compounds in Rectal Glands by GC–MS Analysis

**Fig. 2:**
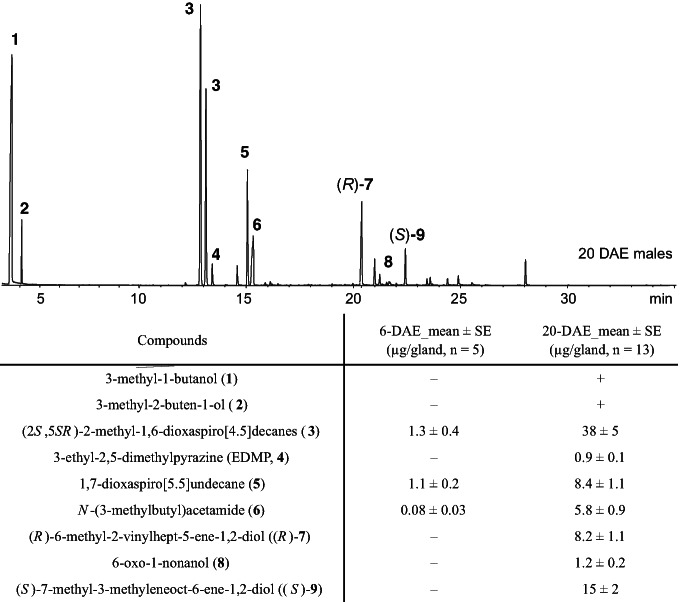
The chromatogram shows detected compounds in rectal gland extracts from 20-DAE males (top), and changes in the quantities of identified endogenous compounds between 6-DAE and 20-DAE in a single rectal gland (bottom). “+” indicates presence detected but not quantified; “–” indicates compound not detected. Quantification was based on calibration curves from authentic standards

The rectal glands of 6-DAE immature males contained mainly three compounds: (2*R*,5*R*)-2-methyl-1,6-dioxaspiro[4.5]decane ((2*R*,5*R*)-**3**), (2*R*,5*S*)-2-methyl-1,6-dioxaspiro[4.5]decane ((2*R*,5*S*)-**3**), 1,7-dioxaspiro[5.5]undecane (**5**) with less than 1.5 µg per gland, whereas *N*-(3-methylbutyl)acetamide (**6**) was present only in trace amounts (Fig. 2). As age increases, the production of spiroacetals **3** and acetamide **6** increased as much as 30-fold and over 70-fold, respectively, while spiroacetal **5** increased about 7-fold in 20-DAE males compared to that of immature males. At this age, six additional compounds were detected in mature males. Upon GC–MS analysis and comparison with authentic standards, four of these were identified as 3-methyl-1-butanol (**1**), 3-methyl-2-buten-1-ol (**2**), EDMP (**4**), and 6-oxo-1-nonanol (**8**); however, these compounds were present at relatively low levels. In males of both ages, the C-2 methyl group of 2-methyl-1,6-dioxaspiro[4.5]decane (**3**) was predominantly *R* configured (80% ee), whereas the spiroacetal center was present as a mixture of epimers. The structures of two major compounds **7** and **9** could not be elucidated by GC–MS analysis alone. Therefore, the identities of these compounds were further elucidated through a synthetic approach.

### Identification of Endogenous Compounds 7 and 9 in Rectal Glands by Synthetic Approach

Detailed structural analyses, combined with synthetic approaches, were employed to identify compounds **7** and **9**. High-resolution electron impact mass spectrometry (HREIMS) coupled to GC indicated that both compounds had the molecular formula C₁₀H₁₈O₂, corresponding to two double bond equivalents. GC–IR analysis showed the absence of carbonyl functionalities and confirmed the presence of two hydroxyl groups in both compounds (Fig. S6 and S7). Hydrogenation of rectal gland extracts demonstrated that each compound contained two double bonds and possessed linear carbon skeletons (Fig. S8–S10). This conclusion was further supported by GC–MS comparisons with in-house–synthesized cyclic diol standards, including 1-(hydroxymethyl)-4-(prop-1-en-2-yl)cyclohexan-1-ol, 2-(hydroxymethyl)-5-(prop-1-en-2-yl)cyclohexan-1-ol, and 1-(hydroxymethyl)-4-(propan-2-ylidene)cyclohexan-1-ol, which exhibited higher linear retention indices and reduced fragmentation patterns compared with the natural compounds (Fig. S11–S17).

Based on these results, three candidate linear diols: 2-(4-methylpent-3-en-1-yl)but-2-ene-1,4-diol (α-acaridiol), 6-methyl-2-vinylhept-5-ene-1,2-diol, and 7-methyl-3-methyleneoct-6-ene-1,2-diol were synthesized and directly compared with the rectal gland extracts. Comparison with these synthetic standards, which showed identical retention times and mass spectra, established the structures of the two natural compounds as 6-methyl-2-vinylhept-5-ene-1,2-diol (**7**) and 7-methyl-3-methyleneoct-6-ene-1,2-diol (**9**), respectively (Fig. S1 and S3). Chiral GC analysis based on comparison with in-house–synthesized standards of known configuration revealed that the major enantiomers of the natural diols were (*R*)-6-methyl-2-vinylhept-5-ene-1,2-diol ((*R*)-**7**) and (*S*)-7-methyl-3-methyleneoct-6-ene-1,2-diol ((*S*)-**9**) (Fig. S19 and S20). These two compounds were the most abundant components in the rectal gland extracts of mature males but were not detected in 6-DAE males.

### Identification of ME Metabolites in Rectal Glands

**Fig. 3:**
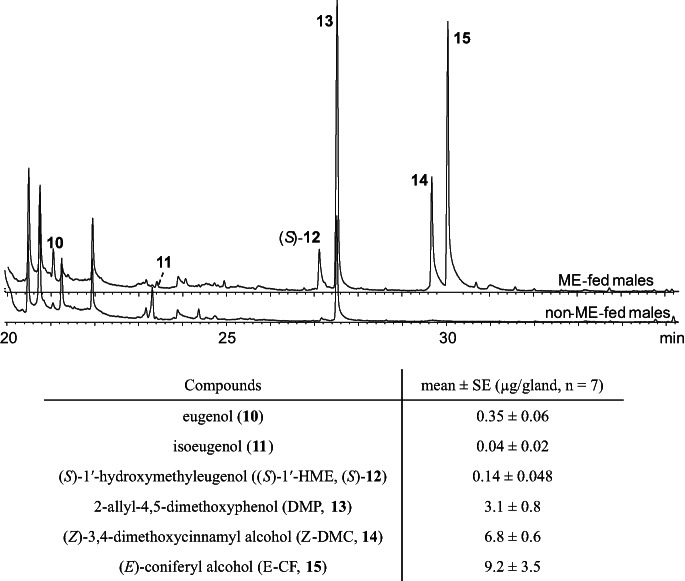
Chromatograms show detected compounds and their composition changes in rectal gland extract before (bottom) and after ME feeding (top), and quantification of identified ME metabolites from a single rectal gland of ME-fed 20-DAE male (bottom). Quantification was based on calibration curves from authentic standards

One day after feeding on ME, six ME metabolites were detected in the rectal glands of mature males (Fig. 3). Comparison with authentic standards identified these compounds as eugenol (**10**), isoeugenol (**11**), 1ʹ-HME (**12**), DMP (**13**), Z-DMC (**14**), and E-CF (**15**). No residual ME was detected at 1-day post ME-feeding. The stereochemistry of C-1’ of 1ʹ-HME (**12**) was predominantly *S* (80% ee). On a quantitative basis, the major constituents were DMP (**13**), Z-DMC (**14**), and E-CF (**15**), present in an approximate ratio of 1:2:3, whereas eugenol (**10**), isoeugenol (**11**), and 1ʹ-HME (**12**) were present only in trace amounts.

### Y-tube Olfactometer Bioassay

**Fig. 4:**
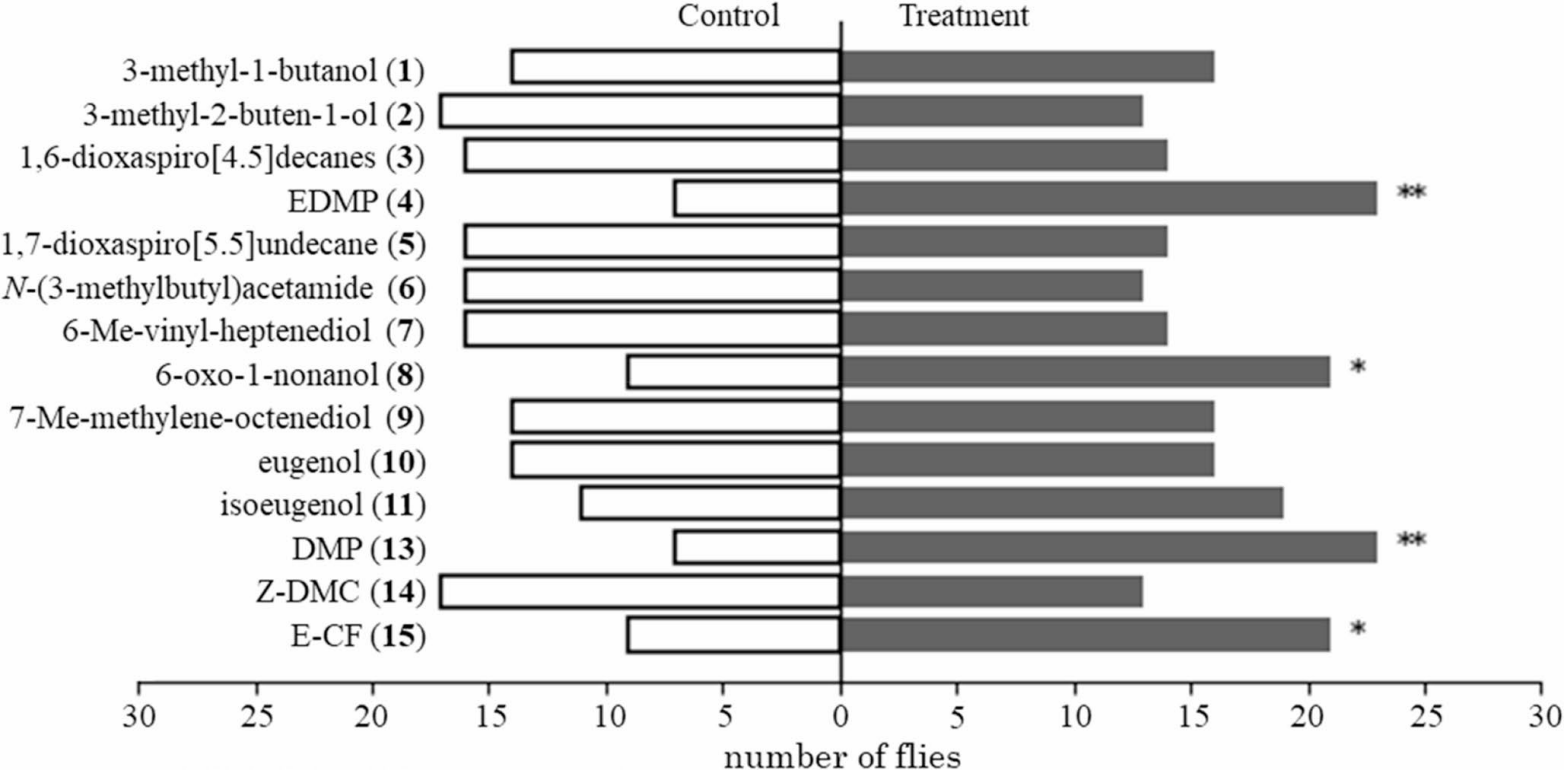
Mature virgin females *B. umbrosa* attraction to synthetic chemicals identified from males’ endogenous rectal glands and ME metabolites in dual-choice assays during courtship period at dusk. Ethanol solution was used as control. A total of 30 flies were tested. “*” indicates significant differences at *p* < 0.05 and “**” indicates *p* < 0.01 according to Yates’ corrected Chi-square test

**Fig. 5:**
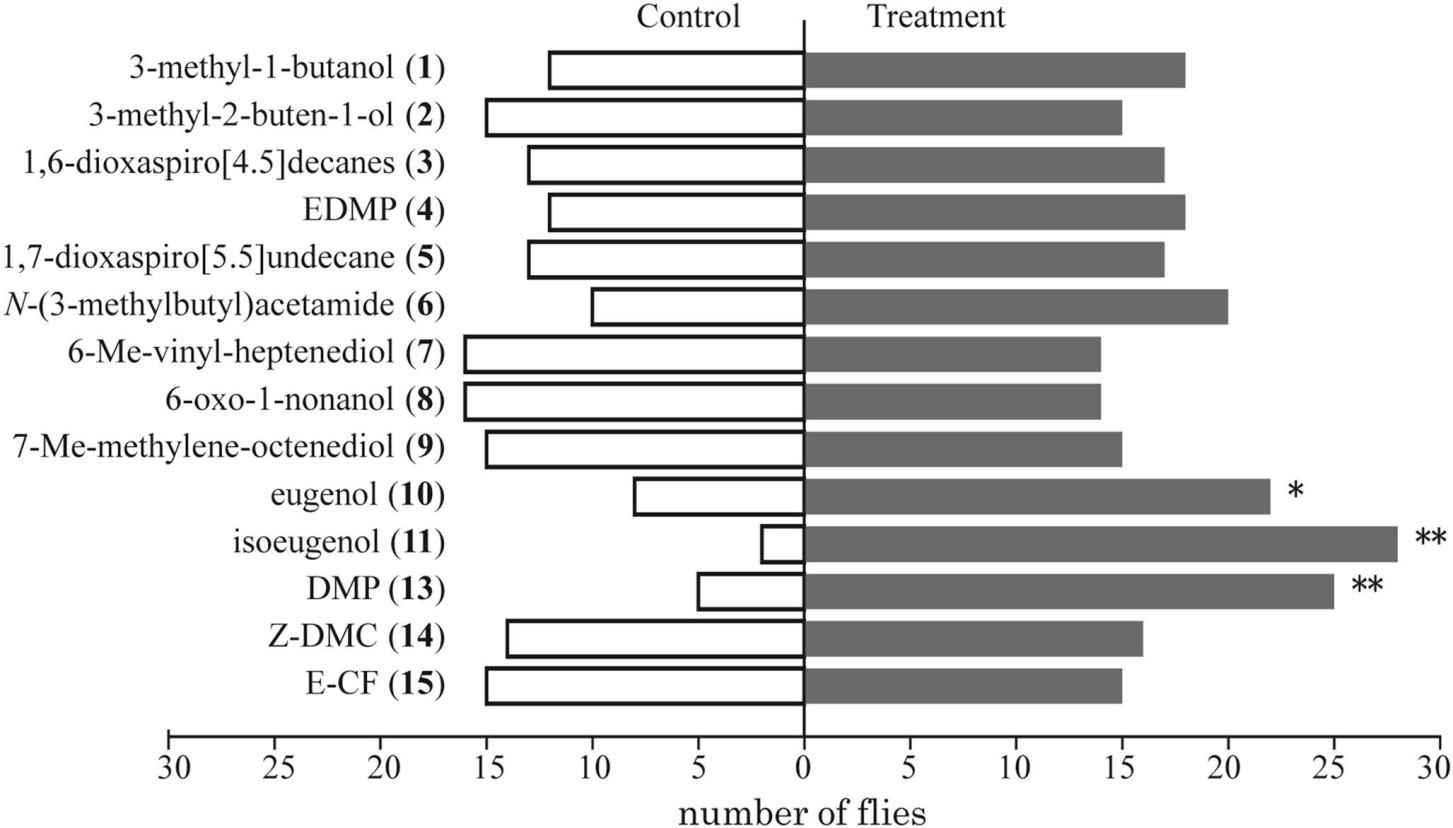
Mature virgin males *B. umbrosa* attraction to synthetic chemicals identified from males’ endogenous rectal glands and ME metabolites in dual-choice assasys during courtship period at dusk. Ethanol solution was used as control. A total of 30 flies were tested. “*” indicates significant differences at *p* < 0.05 and “**” indicates *p* < 0.01 according to Yates’ corrected Chi-square test

Of all the nine endogenous volatile compounds assayed during the courtship period, only two compounds, EDMP (**4**) and 6-oxo-1-nonanol (**8**), were attractive to the mature virgin *B. umbrosa* females (Fig. 4). Compound **8** is more attractive to the female flies than compound **4** (*p* = 0.0062 and 0.045, respectively). Of the five ME metabolites tested, only DMP (**13**) and E-CF (**15**) were significantly attractive to mature females, with DMP (**13**) being more attractive to the females than E-CF (**15**) (Fig. 4). However, none of the endogenous compounds was attractive to the mature males when tested during the courtship period. Nevertheless, three ME metabolites—eugenol (**10**), isoeugenol (**11**), and DMP (**13**)—significantly attracted males (*p* = 1.06 × 10^− 2^, 2.07 × 10^− 6^ and 2.61 × 10^− 4^, respectively), with isoeugenol and DMP being more attractive than eugenol (Fig. 5).

## Discussion

The male glandular chemistry of the *Artocarpus* fruit fly changes in composition and quantity with sexual maturity attainment. Among the nine endogenous rectal compounds produced by mature males, none are attractive to the males, while only two compounds, EDMP and keto alcohol **8**, were attractive to females during the courtship period, thus acting as sex pheromones for the *Artocarpus* fruit fly. EDMP elicited a stronger attraction response in virgin females than the keto alcohol **8**. Pyrazines with diverse substitution patterns are widely recognized as key semiochemicals involved in aggregation, sex, or alarm signaling across a broad range of biological taxa, both intra- and inter-specifically (Khashaveh et al. 2025). Notably, EDMP also functions as the recruitment pheromone in several *Pogonomyrmex* ant species (Cross et al. 1979; Hölldobler et al. 2001). Within Tephritidae, males of *B. dorsalis* produce 2,3,5-trimethylpyrazine (TMP) and 2,3,5,6-tetramethylpyrazine (TTMP), both of which attract virgin females (Perkins et al. 1990; Ren et al. 2021). These pyrazines were subsequently shown to be synthesized not by the insect but by *Bacillus* spp. inhabiting the male rectal glands (Ren et al. 2021). Considering the recurring involvement of microbial partners in pyrazine biosynthesis across diverse insect groups (Khashaveh et al. 2025), a bacterial origin for EDMP in *B. umbrosa* represents a plausible and testable hypothesis.

In *B. carambolae*, both keto alcohol **8** and acetamide **6** were clearly attractive to virgin females (Wee and Tan 2005a). In contrast, virgin females of *B. umbrosa* responded only to keto alcohol **8**, while acetamide **6** elicited no attraction, although there was a significant 70-fold increase in quantity with sexual maturity. Interestingly, *B. umbrosa* males produce acetamide **6** as early as 6-DAE, whereas *B. carambolae* begins producing the same compound from 19-DAE concomitant with sexual maturity attainment (Wee and Tan 2005a). However, since we did not test these compounds in all possible blends, the possibility that these biologically ‘inactive’ compounds could have synergistic effects on the attractive compounds could not be entirely ruled out.

Spiroacetals often play important roles in the semiochemistry of *Bactrocera* species (Baker et al. 1980; Baker and Bacon 1985; Haniotakis et al. 1986). However, the two spiroacetals **3** and **5** detected here did not elicit attraction in either females or males, although the quantity of the former increased by 30-fold at maturity. Four alcohols, **1**, **2**, **7**, and **9**, were produced only after sexual maturation; however, none appeared to contribute significantly to intraspecific communication in *B. umbrosa* as evidenced by the behavioral assays. Future study may investigate the actual biological roles of these compounds since the production of endogenous chemicals entails a significant cost to the flies that might otherwise be used for growth (Pasteels et al. 1983).

From a biosynthetic perspective, diols **7** and **9** are particularly noteworthy. To our knowledge, this study represents the first report of diols **7** and **9** occurring in insects. Both compounds have previously been reported as *β*-myrcene metabolites when *β*-myrcene was experimentally supplied to microorganisms, including *Aspergillus niger* and *Pleurotus ostreatus*, as well as to the cutworm *Spodoptera litura* (Yamazaki et al. 1988; Miyazawa and Murata 2000; Krings et al. 2008). *β*-Myrcene is one of the most abundant acyclic monoterpenes in nature and represents a xenobiotic compound for microorganisms and insects. In these earlier studies, *β*-myrcene was biotransformed into several oxygenated derivatives, including α-acaridiol and the diols **7** and **9**. Recently, diol **9** was reported as a natural product from the plant *Artemisia argyi* (Huang et al. 2024). These diols are most plausibly formed from *β*-myrcene via epoxidation followed by hydrolytic ring opening, as proposed previously for microbial systems (Krings et al. 2008). The predominance of a single enantiomer for each compound further suggests the involvement of enzyme-mediated processes in *B. umbrosa*.

It is known that *Bactrocera* males that are attracted to and consumed ME would produce and accumulate one to three ME metabolites in their rectal glands, depending on species (Nishida et al. 1988a, b; Wee and Tan 2007a; Tan et al. 2011). In the case of *B. umbrosa*, ME-fed males produce as many as six ME metabolites. DMP, Z-DMC, E-CF and Z-CF represent common oxidized ME derivatives in some *Bactrocera* species (Nishida et al. 1988a, b; Wee and Tan 2007a; Tan et al. 2011). Two of them—DMP and E-CF— were being produced in *B. umbrosa* and exerted attractive effects on virgin females in this study, consistent with previous findings in *B. dorsalis* (Tan and Nishida 1996, 1998; Hee and Tan 1998; Khoo et al. 2000). In *B. dorsalis*, E-CF was shown to be more attractive to females than DMP while DMP is most attractive to the males during courtship period (Tan 1996; Hee and Tan 1998; Khoo et al. 2000). These showed that E-CF acts as a better sex pheromone than DMP, and DMP acts as an aggregation pheromone in *B. dorsalis*. E-CF was also shown as a sex pheromone that enhanced mating success of *B. carambolae* males (Wee et al. 2007a, 2007b). However, in the case of *B. umbrosa*, DMP is not only attractive to the males, as reported in other *Bactrocera* species, but it is also shown to be more attractive to virgin females than E-CF, in contrast to other *Bactrocera* species. Hence, this suggests that DMP plays a more significant role than other ME-metabolites as a sex and aggregation pheromone in the *Artocarpus* fruit fly.

Two additional ME metabolites, eugenol and isoeugenol, were also identified from the rectal glands of ME-fed males. The present results indicate that eugenol and isoeugenol are both attractive to males, with isoeugenol being more attractive than eugenol. Nevertheless, their ecological relevance in the chemical ecology of the *Artocarpus* fruit fly remains unclear, given the extremely small quantities produced in the rectal glands after feeding on ME. Isoeugenol has been shown to be a highly effective, species-specific male lure for *B. curvipennis* (Royer et al. 2019). In the field, isoeugenol-baited traps captured low numbers of *B. umbrosa*, while eugenol never captured any *B. umbrosa* (Wee unpublished data). However, to date, no documented evidence supports the pheromonal role for these compounds in *Bactrocera* species.

Another ME-metabolite, 1ʹ-HME, was detected in the rectal gland of *B. umbrosa*, which, to our knowledge, is the first time this compound has been detected in insects. This compound, a proximate carcinogen, is a well-known mammalian ME metabolite formed via P450-dependent oxidation (Solheim and Scheline 1976; Gardner et al. 1997). The predominance of a single enantiomer and its unique occurrence in insects suggest the involvement of a species-specific, enzyme-mediated metabolic process in *B. umbrosa*. However, since the structure was determined only after the completion of behavioral experiments, the biological function of this compound remains to be investigated.

Given its status as a host specialist that is largely restricted to *Artocarpus* fruits and exhibits limited dispersal capability (Hardy 1973; Tan and Jaal 1986; Allwood et al. 1999; Lauciello et al. 2024), B. *umbrosa* is likely to maintain its locally structured populations. Such ecological specialization may constrain pheromone evolution and promote maintenance of stable endogenous blends (Symonds et al. 2008, 2009; Roelofs and Rooney 2003), suggesting that *B. umbrosa* retains a consistent endogenous signal, primarily through EDMP and keto alcohol **8**, for reliable mate recognition within its narrow habitat. Future studies should investigate the ecological significance of other endogenous compounds, particularly the possible function of keto alcohol **8** as an allomone since the same compound was also reported to deter predation in *B. carambolae* (Wee and Tan 2005b).

Given the fact that *B. umbrosa* males respond to ME at a very young age and well before commencement of any sexual activities (Wee et al. 2018), and with more than 450 ME-containing plant species widely distributed in the tropics (Tan and Nishida 2012), it is not surprising that the males could get access to ME and transiently amplify male attractiveness or competitive ability. The ME-derived metabolites thus function as facultative, condition-dependent enhancers of the species’ chemical communication. The use of plant-derived compounds to strengthen or modulate pheromonal signals is well established in insect chemical ecology (Reddy and Guerrero 2004; Mbaluto et al. 2020). Accordingly, the opportunistic incorporation of ME metabolites allows *B. umbrosa* to boost the attractiveness of sexual signals when ME is available, while relying on a robust endogenous pheromone blend in its absence, resulting in a flexible yet evolutionarily stable communication strategy. These findings contribute to a better understanding of the pheromone-mediated communication system of the *Artocarpus* fruit fly. This enhanced knowledge provides an important foundation for developing future pheromone-based monitoring strategies and control methods against this pest species.

## Supplementary Information

Below is the link to the electronic supplementary material.

Supplementary Material 1

## Data Availability

Raw data will be made available upon request.
