# Supplementary material for: Endogenous Glandular Chemistry and Methyl Eugenol–derived Metabolites in the Pheromone Communication of Bactrocera umbrosa
Source: J Chem Ecol. 2026 Jun 13;52(3):53. doi: 10.1007/s10886-026-01726-2 (PMC13264548; doi:10.1007/s10886-026-01726-2)
Supplement: Supplementary file 1 — Supplementary Material 1 [file 10886_2026_1726_MOESM1_ESM.pdf]

SUPPORTING INFORMATION

Endogenous Glandular Chemistry and Methyl Eugenol–Derived Metabolites in the Pheromone Communication of *Bactrocera umbrosa*

Tatsuya Kiuchi,[1] Suk-Ling Wee,[2] Stefan Schulz[1,*]

Addresses

[1] Institute of Organic Chemistry, Technische Universität Braunschweig, Hagenring 30, Braunschweig 38106, Germany
[2] Centre for Insect Systematics, Department of Biological Sciences and Biotechnology, Faculty of Science and Technology, Universiti Kebangsaan Malaysia, 43600 Bangi, Selangor Darul Ehsan, Malaysia

Table of Contents

1 GC-MS Information of Natural Samples..4
Fig. S1 EI-MS (70 eV) of (R)-6-methyl-2-vinylhept-5-ene-1,2-diol ((*R*)-**7**)..4
Fig. S2 EI-MS (70 eV) of 6-oxononan-1-ol (**8**)..4
Fig. S3 EI-MS (70 eV) of (*S*)-7-methyl-3-methyleneoct-6-ene-1,2-diol ((*S*)-**9**)..5
Fig. S4 EI-MS (70 eV) of (*S*)-1-(3,4-dimethoxyphenyl)prop-2-en-1-ol ((*S*)-**12**)..5
Fig. S5 EI-MS (70 eV) of 2-allyl-4,5-dimethoxyphenol (DMP, **13**). 5
2. Analytical Information of Two Monoterpenoids..5
Fig. S6 IR spectrum of (*R*)-6-Methyl-2-vinylhept-5-ene-1,2-diol ((*R*)-**7**). 6
Fig. S7 IR spectrum of (*S*)-7-Methyl-3-methyleneoct-6-ene-1,2-diol ((*S*)-**9**)..7
Fig. S8 GC–MS chromatograms of the rectal gland extracts from 20-DAE male *B. umbrosa* before (top) and after (bottom) hydrogenation..7
Fig. S9 EI-MS (70 eV) of hydrogenated (*R*)-6-methyl-2-vinylhept-5-ene-1,2-diol ((*R*)-**7**)..8
Fig. S**10** EI-MS (70 eV) of hydrogenated (S)-7-Methyl-3-methyleneoct-6-ene-1,2-diol ((*S*)-**9**)..8
Fig. S11 Synthesized compounds for structure elucidation: 1-(hydroxymethyl)-4-(prop-1-en-2-yl)cyclohexan-1-ol (**S1**), 2-(hydroxymethyl)-5-(prop-1-en-2-yl)cyclohexan-1-ol (**S2**), 1-(hydroxymethyl)-4-(propan-2-ylidene)cyclohexan-1-ol (**S3**), 2-(4-methylpent-3-en-1-yl)but-2-ene-1,4-diol (α-acaridiol, **S4**), 6-methyl-2-vinylhept-5-ene-1,2-diol (**7**) and 7-methyl-3-methyleneoct-6-ene-1,2-diol (**9**)..9
Fig. S12 EI-MS (70 eV) of *cis*-1-(hydroxymethyl)-4-(prop-1-en-2-yl)cyclohexan-1-ol (*cis*-**S1**)..9
Fig. S13 EI-MS (70 eV) of *trans*-1-(hydroxymethyl)-4-(prop-1-en-2-yl)cyclohexan-1-ol (*tasns*-**S1**). 9
Fig. S14 EI-MS (70 eV) of 2-(hydroxymethyl)-5-(prop-1-en-2-yl)cyclohexan-1-ol (**S2-1**)..9
Fig. S15 EI-MS (70 eV) of 2-(hydroxymethyl)-5-(prop-1-en-2-yl)cyclohexan-1-ol (**S2-2**)..10
Fig. S16 EI-MS (70 eV) of 2-(hydroxymethyl)-5-(prop-1-en-2-yl)cyclohexan-1-ol (**S2-3**)..10
Fig. S17 EI-MS (70 eV) of 1-(hydroxymethyl)-4-(propan-2-ylidene)cyclohexan-1-ol (**S3**). 10
Fig. S18 EI-MS (70 eV) of (*E*)-2-(4-methylpent-3-en-1-yl)but-2-ene-1,4-diol (α-(*E*)-acaridiol, (*E*)-**S4**). 11
Fig. S19 Chiral GC Comparison between synthetic (*S*)-**7** and rectal gland extracts from male *B. umbrosa* using a HYDRODEX β-6TBDM phase (Macherey & Nagel). The temperature program started at 50 °C, increased at 5 °C/min to 80 °C, held for 180 min, and then ramped at 20 °C/min to the final temperature of 320 °C..11
Fig. S20 Chiral GC Comparison between synthetic (*S*)-**9** and rectal gland extracts from male *B. umbrosa* using a Beta-Dex™ 225 chiral capillary column (SUPELCO). The temperature program started at 100 °C, was held for 120 min, and then ramped at 20 °C/min to the final temperature of 250 °C. 11
3. Quantificaiton..12
Fig. S21 Calibration curves showing the relationship between peak area ratios (components/internal standard) and concentration. 1-Decanol (1.0 mM) was used as the internal standard. For compounds that co-eluted with other components, quantification was performed using peak areas of specific m/z ions, normalized to the internal standard. 14
Fig. S22 Quantification of endogenous compounds from single rectal glands of a 6-DAE male *B. umbrosa* (n = 5)..14
Fig. S23 Quantification of endogenous compounds from single rectal glands of a 20-DAE male *B. umbrosa* (n = 13)..15
Fig. S24 Quantification of endogenous compounds from single rectal glands of a ME-fed 20-DAE male *B. umbrosa* 15
(n = 7)..15
4 Experimental Procedures..16
Synthesis of (2*S*, 5*SR*)-2-methyl-1,6-dioxaspiro[4.5]decane (**3**)..16
Fig. S25 Chiral GC comparison between synthetic (*S*)-**3** and rectal gland extracts from male *B. umbrosa* using a HYDRODEX β-6TBDM phase. 17
Synthesis of 3-ethyl-2,5-dimethylpyrazine (**4**) 18
Synthesis of 1,7-dioxaspiro[5.5]undecane (**5**) 18
Synthesis of *N*-isopentylacetamide (**6**) 18
Synthesis of 6-methyl-2-vinylhept-5-ene-1,2-diol (**7**) 18
Synthesis of (*S*)-6-methyl-2-vinylhept-5-ene-1,2-diol ((*S*)-**7**) 20
Synthesis of 6-oxononan-1-ol (**8**) 20
Synthesis of 7-methyl-3-methyleneoct-6-ene-1,2-diol (**9**)..21
Synthesis of (*S*)-7-methyl-3-methyleneoct-6-ene-1,2-diol ((*S*)-**9**)..22
Synthesis of (*S*)-1′-hydroxymethyleugenol ((*S*)-1′-HME, (*S*)-**12**) 22
Fig. S26 Chiral GC comparison between synthetic (*S*)-**12** and rectal gland extracts from a ME-fed male *B. umbrosa* using HYDRODEX β-6TBDM phase (Macherey & Nagel)..23
Synthesis of 2-allyl-4,5-dimethoxyphenol (DMP, **13**)..23
Synthesis of (*Z*)-3,4-dimethoxycinnamyl alcohol (Z-DMC, **14**)..24
Synthesis of (*E*)-coniferyl alcohol (E-CF, **15**) 24
5 NMR Spectra..25
Fig. S27 ^{1}H NMR (300 MHz, $CDCl_3$) and ^{13}C NMR (75.5 MHz, $CDCl_3$) spectra of (3-bromopropoxy)trimethylsilane (**S6**)..25
Fig. S28 ^{1}H NMR (300 MHz, $CDCl_3$) and ^{13}C NMR (75.5 MHz, $CDCl_3$) spectra of (3-iodopropoxy)trimethylsilane (**S7**)..26
Fig. S29 ^{1}H NMR (300 MHz, $CDCl_3$) and ^{13}C NMR (75.5 MHz, $CDCl_3$) spectra of 1,1-dimethyl-2-(propan-2-ylidene)hydrazine (**S9**). 27
Fig. S30 ^{1}H NMR (300 MHz, $CDCl_3$) and ^{13}C NMR (75.5 MHz, $CDCl_3$) spectra of 1,1-dimethyl-2-(propan-2-ylidene)hydrazine (*S*)-**S10**). 28
Fig. S31 ^{1}H NMR (300 MHz, $CDCl_3$) and ^{13}C NMR (75.5 MHz, $CDCl_3$) spectra of (2*S*, 5*SR*)-2-methyl-1,6-dioxaspiro[4.5]decane (**3**). 29
Fig. S32 ^{1}H NMR (300 MHz, $CDCl_3$) and ^{13}C NMR (75.5 MHz, $CDCl_3$) spectra of 3-ethyl-2,5-dimethylpyrazine (**4**). 30
Fig. S33 ^{1}H NMR (300 MHz, $CDCl_3$) and ^{13}C NMR (75.5 MHz, $CDCl_3$) spectra of 1,7-dioxaspiro[5.5]undecane (**5**). 31
Fig. S34 ^{1}H NMR (300 MHz, $CDCl_3$) and ^{13}C NMR (75.5 MHz, $CDCl_3$) spectra of *N*-isopentylacetamide (**6**). 32

Fig. S35 ^{1}H NMR (300 MHz, $CDCl_3$) and ^{13}C NMR (75.5 MHz, $CDCl_3$) spectra of tert-butyldimethyl(oxiran-2-ylmethoxy)silane (**S17**). 33
Fig. S36 ^{1}H NMR (300 MHz, $CDCl_3$) and ^{13}C NMR (75.5 MHz, $CDCl_3$) spectra of 1-((tert-butyldimethylsilyl)oxy)-6-methylhept-5-en-2-ol (**S19**) 34
Fig. S37 ^{1}H NMR (300 MHz, $CDCl_3$) and ^{13}C NMR (75.5 MHz, $CDCl_3$) spectra of 1-((tert-butyldimethylsilyl)oxy)-6-methylhept-5-en-2-one (**S20**). 35
Fig. S38 ^{1}H NMR (300 MHz, $CDCl_3$) and ^{13}C NMR (75.5 MHz, $CDCl_3$) spectra of 3-(((tert-butyldimethylsilyl)oxy)methyl)-7-methylocta-1,6-dien-3-ol (**S21**). 36
Fig. S39 ^{1}H NMR (300 MHz, $CDCl_3$) and ^{13}C NMR (75.5 MHz, $CDCl_3$) spectra of 6-methyl-2-vinylhept-5-ene-1,2-diol (**7**). 37
Fig. S40 ^{1}H NMR (300 MHz, $CDCl_3$) and ^{13}C NMR (75.5 MHz, $CDCl_3$) spectra of 4-(4-methylpent-3-en-1-yl)-4-vinyl-1,3-dioxolan-2-one (**S22**). 38
Fig. S41 ^{1}H NMR (300 MHz, $CDCl_3$) and ^{13}C NMR (75.5 MHz, $CDCl_3$) spectra of (*S*)-6-methyl-2-vinylhept-5-ene-1,2-diol ((*S*)-**7**). .. 39
Fig. S42 ^{1}H NMR (300 MHz, $CDCl_3$) and ^{13}C NMR (75.5 MHz, $CDCl_3$) spectra of 6-((tert-butyldiphenylsilyl)oxy)-*N*-methoxy-*N*-methylhexanamide (**S24**). 40
Fig. S43 ^{1}H NMR (300 MHz, $CDCl_3$) and ^{13}C NMR (75.5 MHz, $CDCl_3$) spectra of 9-((tert-butyldiphenylsilyl)oxy)nonan-4-one (**S25**). 41
Fig. S44 ^{1}H NMR (300 MHz, $CDCl_3$) and ^{13}C NMR (75.5 MHz, $CDCl_3$) spectra of 6-oxo-nonan-1-ol (**8**). 42
Fig. S45 ^{1}H NMR (300 MHz, $CDCl_3$) and ^{13}C NMR (75.5 MHz, $CDCl_3$) spectra of (3-methyl-3-(4-methylpent-3-en-1-yl)oxiran-2-yl)methanol (**S27**). 43
Fig. S46 ^{1}H NMR (300 MHz, $CDCl_3$) and ^{13}C NMR (75.5 MHz, $CDCl_3$) spectra of 7-methyl-3-methyleneoct-6-ene-1,2-diol (**9**). 44
Fig. S47 ^{1}H NMR (300 MHz, $CDCl_3$) and ^{13}C NMR (75.5 MHz, $CDCl_3$) spectra of ((2*R*,3*R*)-3-methyl-3-(4-methylpent-3-en-1-yl)oxiran-2-yl)methanol ((2*R*,3*R*)-**S27**). 45
Fig. S48 ^{1}H NMR (300 MHz, $CDCl_3$) and ^{13}C NMR (75.5 MHz, $CDCl_3$) spectra of (*S*)-7-methyl-3-methyleneoct-6-ene-1,2-diol ((*S*)-**9**). 46
Fig. S49 ^{1}H NMR (300 MHz, $CDCl_3$) and ^{13}C NMR (75.5 MHz, $CDCl_3$) spectra of 1-(3,4-dimethoxyphenyl)prop-2-en-1-ol (**12**). 47
Fig. S50 ^{1}H NMR (300 MHz, $CDCl_3$) and ^{13}C NMR (75.5 MHz, $CDCl_3$) spectra of (*S*)-1-(3,4-dimethoxyphenyl)prop-2-en-1-ol ((*S*)-**12**). 48
Fig. S51 ^{1}H NMR (300 MHz, $CDCl_3$) and ^{13}C NMR (75.5 MHz, $CDCl_3$) spectra of ethyl 3,4-dimethoxyphenol (**S29**). 49
Fig. S52 ^{1}H NMR (300 MHz, $CDCl_3$) and ^{13}C NMR (75.5 MHz, $CDCl_3$) spectra of 4-(allyloxy)-1,2-dimethoxybenzene (**S30**). 50
Fig. S53 ^{1}H NMR (300 MHz, $CDCl_3$) and ^{13}C NMR (75.5 MHz, $CDCl_3$) spectra of 2-allyl-4,5-dimethoxyphenol (**13**). 51
Fig. S54 ^{1}H NMR (300 MHz, $CDCl_3$) and ^{13}C NMR (75.5 MHz, $CDCl_3$) spectra of ethyl (*Z*)-3-(3,4-dimethoxyphenyl)acrylate (**S31**). 52
Fig. S55 ^{1}H NMR (300 MHz, $CDCl_3$) and ^{13}C NMR (75.5 MHz, $CDCl_3$) spectra of (*Z*)-3-(3,4-dimethoxyphenyl)prop-2-en-1-ol (**14**). 53
Fig. S56 ^{1}H NMR (300 MHz, $CDCl_3$) and ^{13}C NMR (75.5 MHz, $CDCl_3$) spectra of (*E*)-3-(4-hydroxy-3-methoxyphenyl)acrylate (**S33**). 54
Fig. S57 ^{1}H NMR (300 MHz, $CDCl_3$) and ^{13}C NMR (75.5 MHz, $CDCl_3$) spectra of (*E*)-4-(3-hydroxyprop-1-en-1-yl)-2-methoxyphenol (**15**). 55
6 References 56

1 GC-MS Information of Natural Samples

3-Methyl-1-butanol (**1**). GC linear retention index on HP-5MS: 804. EI-MS (70 eV): *m/z* (%) = 55 (100), 70 (79), 41 (59), 42 (58), 43 (50), 39 (29), 57 (24), 45 (14), 56 (12), 69 (7).

3-Methyl-2-buten-1-ol (**2**). GC linear retention index on HP-5MS: 821. EI-MS (70 eV): *m/z* (%) = 86 ([M]$^+$, 22), 71 (100), 41 (43), 43 (34), 53 (26), 39 (25), 67 (14), 68 (14), 55 (6), 40 (5).

(2*R*,5*SR*)-2-Methyl-1,6-dioxaspiro[4.5]decanes (**3**). GC linear retention index on HP-5MS: 1049, 1071. EI-MS (70 eV): *m/z* (%) = 156 ([M]$^+$, 3), 101 (100), 98 (50), 55 (37), 83 (37), 100 (27), 56 (25), 43 (23), 41 (18), 112 (15), 111 (14).

3-Ethyl-2,5-dimethylpyrazine (**4**). GC linear retention index on HP-5MS: 1081. EI-MS (70 eV): *m/z* (%) = 136 ([M]$^+$, 75), 135 (100), 42 (23), 39 (16), 108 (16), 107 (13), 56 (12), 40 (7), 137 (6), 53 (6).

1,7-Dioxaspiro[5.5]undecane (**5**). GC linear retention index on HP-5MS: 1132. EI-MS (70 eV): *m/z* (%) = 156 ([M]$^+$, 8), 101 (100), 98 (94), 100 (56), 55 (45), 83 (33), 41 (26), 43 (20), 56 (19), 111 (16), 42 (13).

N-(3-Methylbutyl)acetamide (**6**). GC linear retention index on HP-5MS: 1140. EI-MS (70 eV): *m/z* (%) = 129 ([M]$^+$, 10), 73 (100), 43 (92), 72 (86), 44 (53), 60 (37), 86 (34), 41 (24), 55 (21), 114 (19), 42 (17).

(*R*)-6-Methyl-2-vinylhept-5-ene-1,2-diol ((*R*)-**7**). GC linear retention index on HP-5MS: 1314. EI-MS (70 eV): *m/z* (%) = 170 ([M]$^+$, 1), 69 (100), 41 (55), 55 (48), 121 (31), 83 (24), 43 (17), 93 (16), 79 (15), 70 (14), 67 (14).

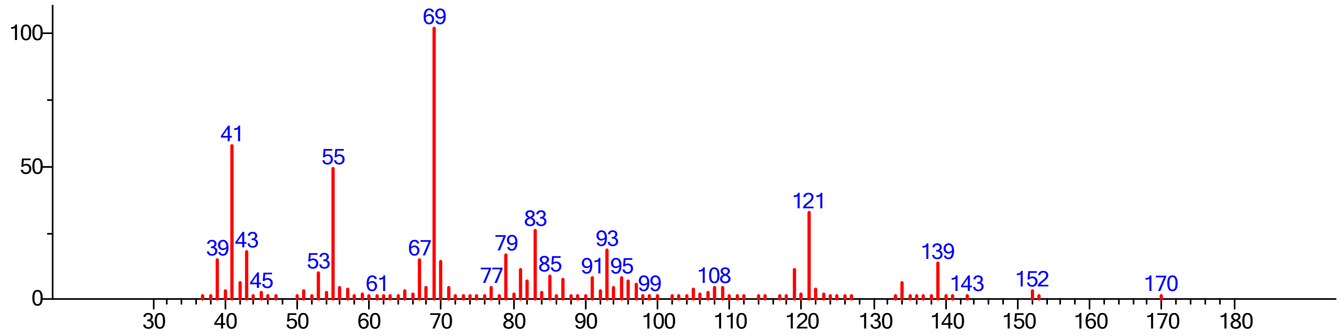

Fig. S1 EI-MS (70 eV) of (R)-6-methyl-2-vinylhept-5-ene-1,2-diol ((*R*)-**7**).

6-Oxononan-1-ol (**8**). GC linear retention index on HP-5MS: 1346. EI-MS (70 eV): *m/z* (%) = 158 ([M]$^+$, 1), 71 (100), 69 (99), 43 (96), 41 (66), 58 (61), 86 (48), 55 (46), 97 (41), 73 (19), 79 (18).

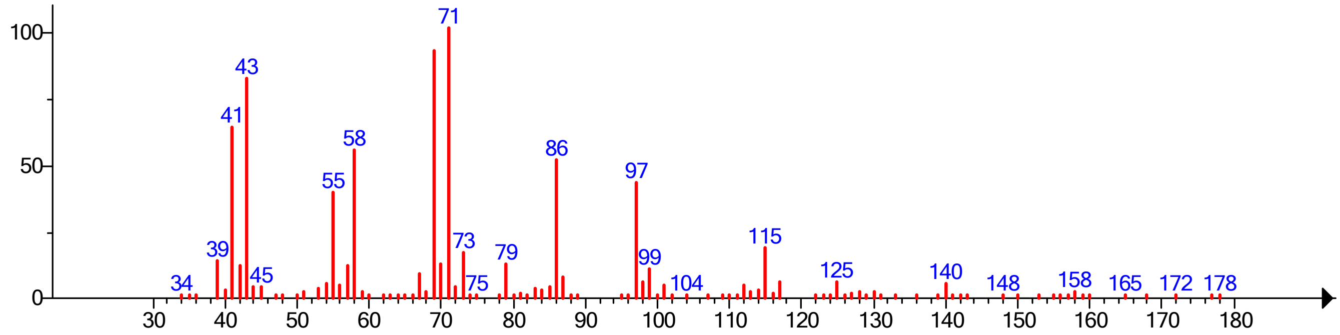

Fig. S2 EI-MS (70 eV) of 6-oxononan-1-ol (**8**).

(*S*)-7-Methyl-3-methyleneoct-6-ene-1,2-diol ((*S*)-**9**). GC linear retention index on HP-5MS: 1391. EI-MS (70 eV): *m/z* (%) = 170 ([M]$^+$, <1), 69 (100), 41 (66), 109 (23), 101 (19), 55 (18), 43 (18), 67 (17), 39 (15), 70 (13), 95 (13).

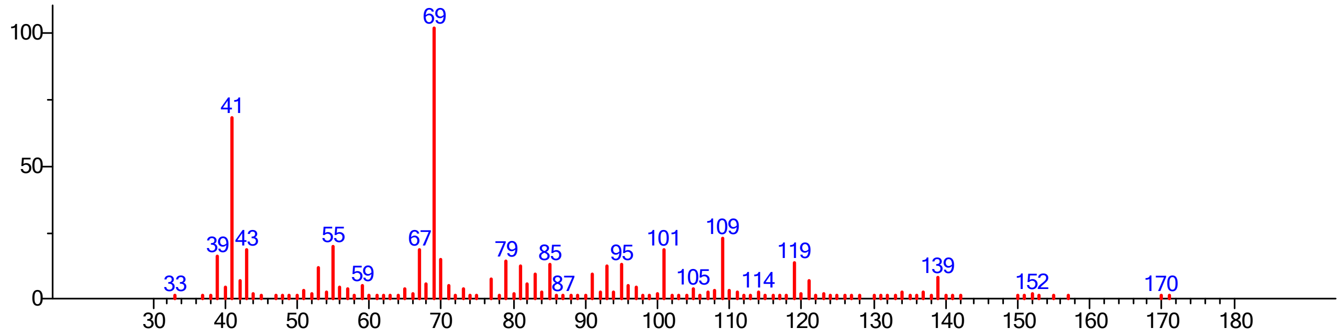

Fig. S3 EI-MS (70 eV) of (*S*)-7-methyl-3-methyleneoct-6-ene-1,2-diol ((*S*)-**9**).

Eugenol (**10**). GC linear retention index on HP-5MS: 1356. EI-MS (70 eV): *m/z* (%) = 164 ([M]$^+$, 100), 149 (32), 103 (29), 77 (28), 131 (26), 91 (25), 137 (19), 104 (17), 133 (17), 55 (15).

Isoeugenol (**11**). GC linear retention index on HP-5MS: 1449. EI-MS (70 eV): *m/z* (%) = 164 ([M]$^+$, 100), 149 (31), 77 (25), 103 (23), 91 (22), 131 (19), 55 (13), 133 (13), 121 (12), 104 (11).

(*S*)-1′-hydroxymethyleugenol ((*S*)-1′-HME, (*S*)-**12**). GC linear retention index on HP-5MS: 1608. EI-MS (70 eV): *m/z* (%) = 194 (100), 139 (91), 55 (58), 163 (58), 151 (34), 165 (28), 77 (25), 138 (21), 91 (21), 79 (20).

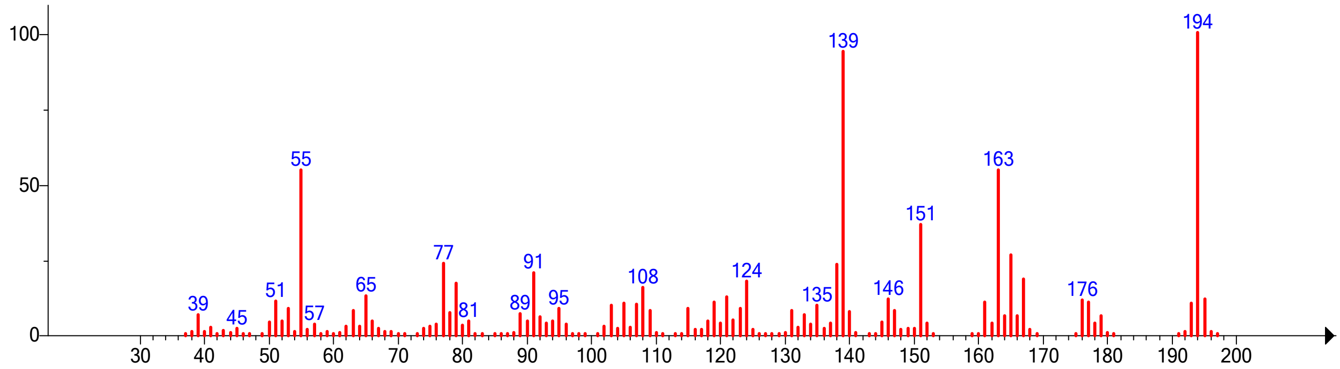

Fig. S4 EI-MS (70 eV) of (*S*)-1-(3,4-dimethoxyphenyl)prop-2-en-1-ol ((*S*)-**12**).

2-Allyl-4,5-dimethoxyphenol (DMP, **13**). GC linear retention index on HP-5MS: 1626. EI-MS (70 eV): *m/z* (%) = 194 ([M]$^+$, 100), 179 (85), 123 (29), 69 (22), 91 (20), 151 (14), 77 (14), 195 (12), 79 (11), 163 (9).

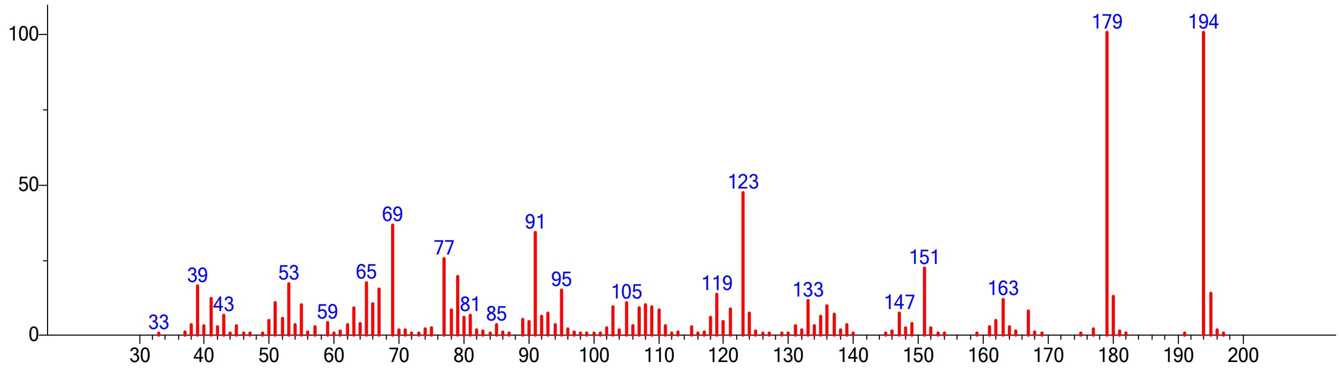

Fig. S5 EI-MS (70 eV) of 2-allyl-4,5-dimethoxyphenol (DMP, **13**).

(*Z*)-3,4-Dimethoxycinnamyl alcohol (Z-DMC, **14**). GC linear retention index on HP-5MS: 1724. EI-MS (70 eV): *m/z* (%) = 194 ([M]$^+$, 67), 151 (100), 138 (51), 91 (29), 77 (22), 119 (14), 165 (13), 55 (12), 79 (12), 152 (11).

(*E*)-Coniferyl alcohol (E-CF, **15**). GC linear retention index on HP-5MS: 1751. EI-MS (70 eV): *m/z* (%) = 180 ([M]$^+$,76), 137 (100), 124 (51), 91 (34), 119 (26), 77 (17), 109 (17), 138 (16), 103 (15), 147 (13).

2. Analytical Information of Two Monoterpenoids

HREIMS coupled to GC

(*R*)-6-Methyl-2-vinylhept-5-ene-1,2-diol ((*R*)-**7**). HREIMS *m/z* calcd. for $C_{10}H_{18}O_2$: 170.13068 [M]$^+$, found: 170.13033.

(*S*)-7-Methyl-3-methyleneoct-6-ene-1,2-diol ((*S*)-**9**). HREIMS *m/z* calcd. for $C_{10}H_{18}O_2$:170.13068 $[M]^+$, found: 170.13013.

GC-IR

(*R*)-6-Methyl-2-vinylhept-5-ene-1,2-diol ((*R*)-**7**). FT-IR: ν/cm^{-1} = 3324, 2968, 2925, 2858, 1450, 1378, 1308, 1262, 1197, 1086, 1047, 996, 924, 843, 805.
(*S*)-7-Methyl-3-methyleneoct-6-ene-1,2-diol ((*S*)-**9**). FT-IR: ν/cm^{-1} = 3315, 2970, 2916, 2854, 1652, 1447, 1379, 1326, 1092, 1047, 1032, 988, 905, 832.

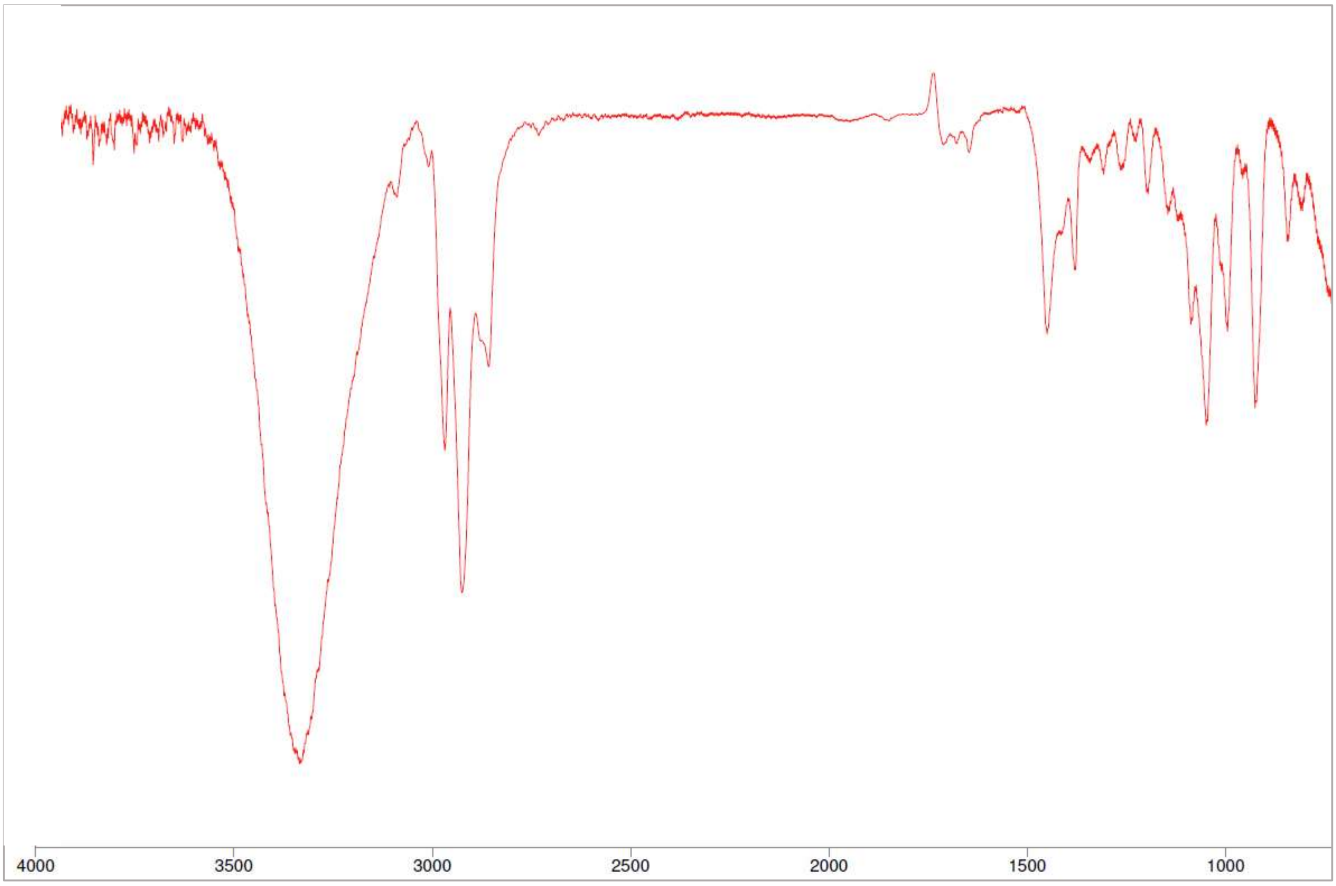

Fig. S6 IR spectrum of (*R*)-6-Methyl-2-vinylhept-5-ene-1,2-diol ((*R*)-**7**).

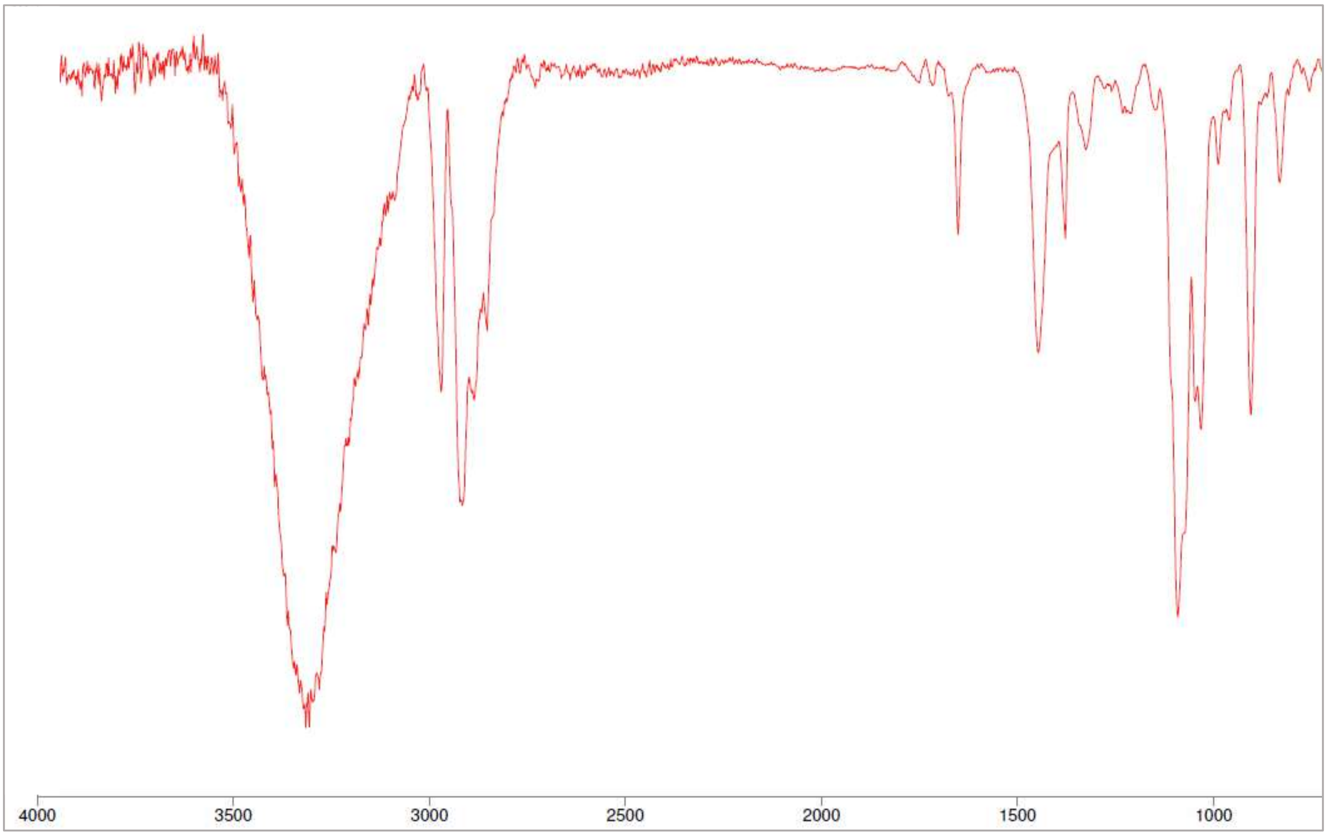

Fig. S7 IR spectrum of (*S*)-7-Methyl-3-methyleneoct-6-ene-1,2-diol ((*S*)-**9**).

Hydrogenation

Hydrogenated (*R*)-6-methyl-2-vinylhept-5-ene-1,2-diol ((*R*)-**7**). EI-MS (70 eV): *m/z* (%) = 69 (100), 89 (53), 43 (39), 143 (28), 57 (25), 41 (24), 83 (23), 55 (22), 109 (20), 71 (17).

Hydrogenated (*S*)-7-methyl-3-methyleneoct-6-ene-1,2-diol ((*S*)-**9**). EI-MS (70 eV): *m/z* (%) = 69 (100), 43 (39), 57 (30), 55 (29), 143 (28), 41 (27), 83 (26), 71 (13), 61 (12), 125 (10).

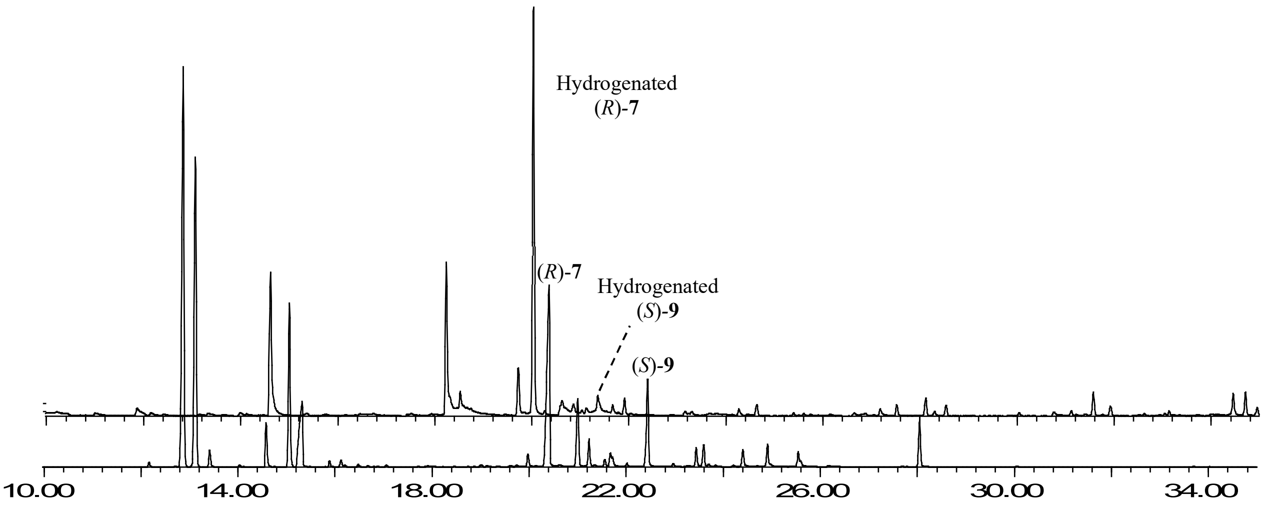

Fig. S8 GC–MS chromatograms of the rectal gland extracts from 20-DAE male *B. umbrosa* before (top) and after (bottom) hydrogenation.

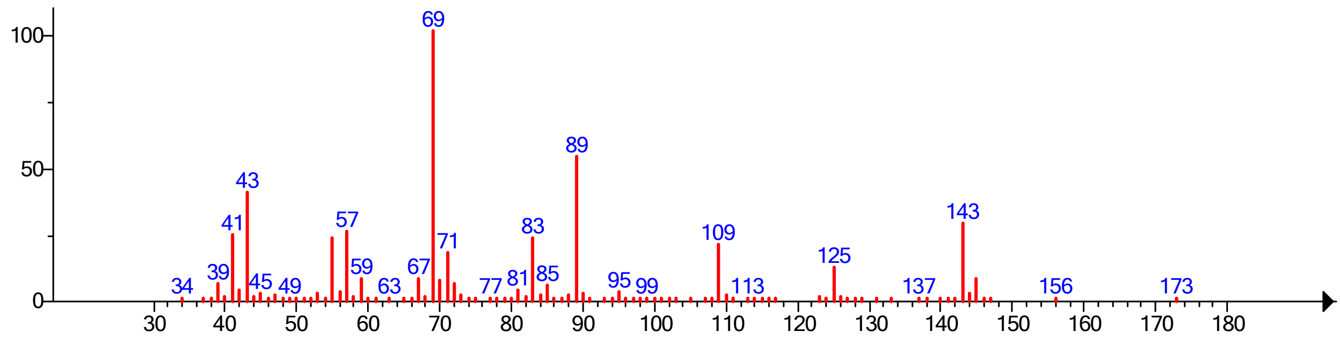

Fig. S9 EI-MS (70 eV) of hydrogenated (*R*)-6-methyl-2-vinylhept-5-ene-1,2-diol ((*R*)-**7**).

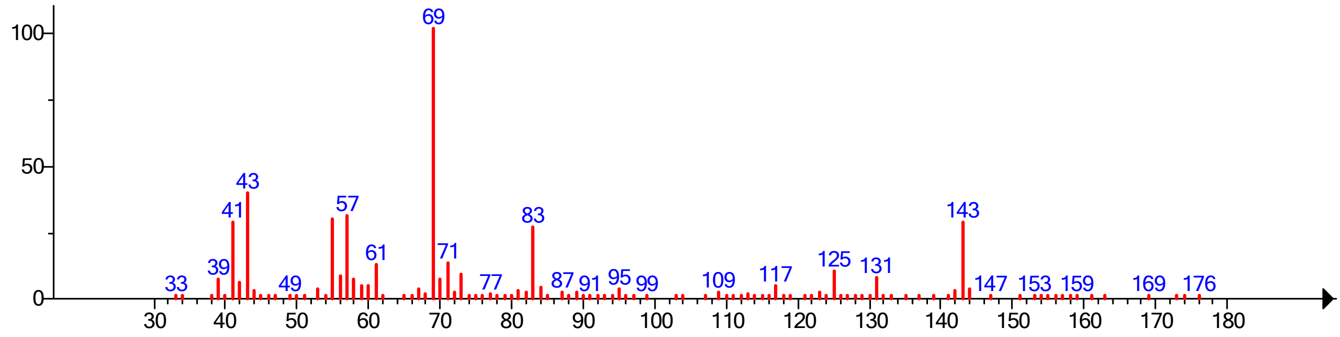

Fig. S**10** EI-MS (70 eV) of hydrogenated (S)-7-Methyl-3-methyleneoct-6-ene-1,2-diol ((*S*)-**9**).

Synthesis of Putative Compounds

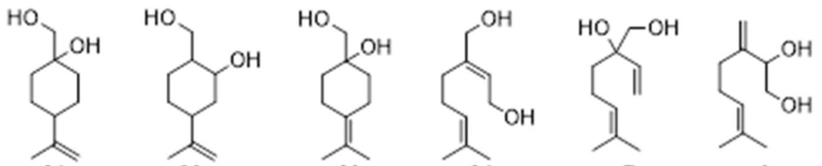

S1 S2 S3 S4 7 9

Fig. S11 Synthesized compounds for structure elucidation: 1-(hydroxymethyl)-4-(prop-1-en-2-yl)cyclohexan-1-ol (**S1**), 2-(hydroxymethyl)-5-(prop-1-en-2-yl)cyclohexan-1-ol (**S2**), 1-(hydroxymethyl)-4-(propan-2-ylidene)cyclohexan-1-ol (**S3**), 2-(4-methylpent-3-en-1-yl)but-2-ene-1,4-diol (α-acaridiol, **S4**), 6-methyl-2-vinylhept-5-ene-1,2-diol (**7**) and 7-methyl-3-methyleneoct-6-ene-1,2-diol (**9**).

cis-1-(hydroxymethyl)-4-(prop-1-en-2-yl)cyclohexan-1-ol (*cis*-**S1**). GC linear retention index on HP-5MS: 1403. EI-MS (70 eV): *m/z* (%) = 69 (100), 41 (92), 55 (87), 83 (84), 43 (69), 139 (66), 93 (58), 67 (57), 79 (57), 39 (46).

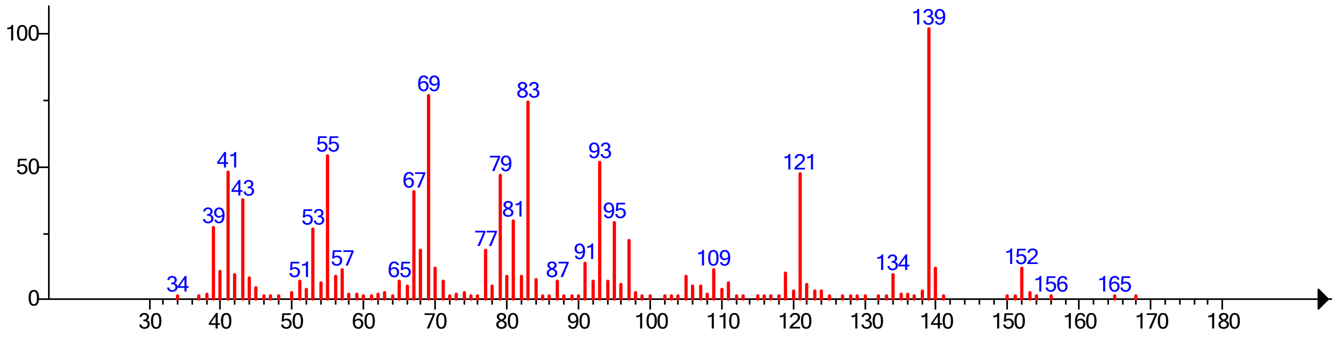

Fig. S12 EI-MS (70 eV) of *cis*-1-(hydroxymethyl)-4-(prop-1-en-2-yl)cyclohexan-1-ol (*cis*-**S1**).

trans-1-(hydroxymethyl)-4-(prop-1-en-2-yl)cyclohexan-1-ol (*trans*-**S1**). GC linear retention index on HP-5MS: 1407. EI-MS (70 eV): *m/z* (%) = 69 (100), 55 (87), 41 (81), 83 (80), 43 (64), 93 (53), 67 (53), 79 (51), 139 (51), 39 (47).

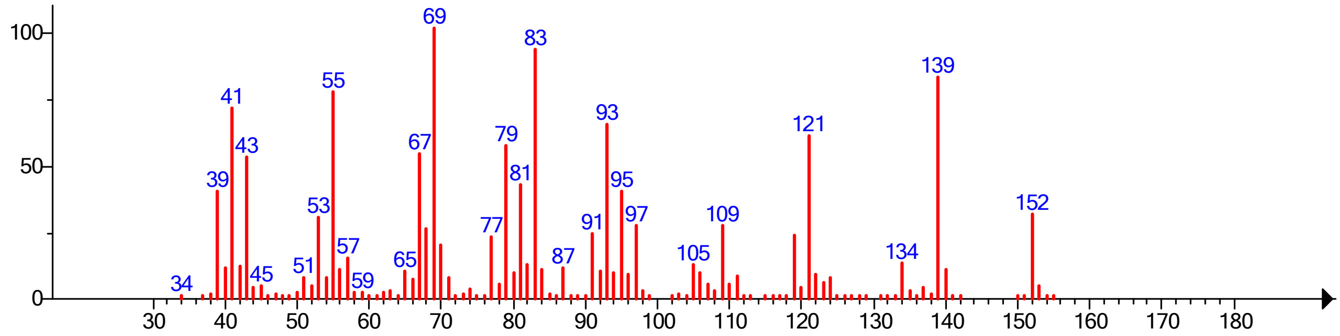

Fig. S13 EI-MS (70 eV) of *trans*-1-(hydroxymethyl)-4-(prop-1-en-2-yl)cyclohexan-1-ol (*tasns*-**S1**).

Three major diastereomers (**S2-1**, **S2-2**, **S2-3**) were obtained as 2-(hydroxymethyl)-5-(prop-1-en-2-yl)cyclohexan-1-ol
S2-1. GC linear retention index on HP-5MS: 1493. EI-MS (70 eV): *m/z* (%) = 170 ($[M]^+$, <1), 119 (100), 121 (69), 79 (69), 41 (60), 67 (57), 93 (56), 91 (55), 55 (50), 134 (44), 69 (43).

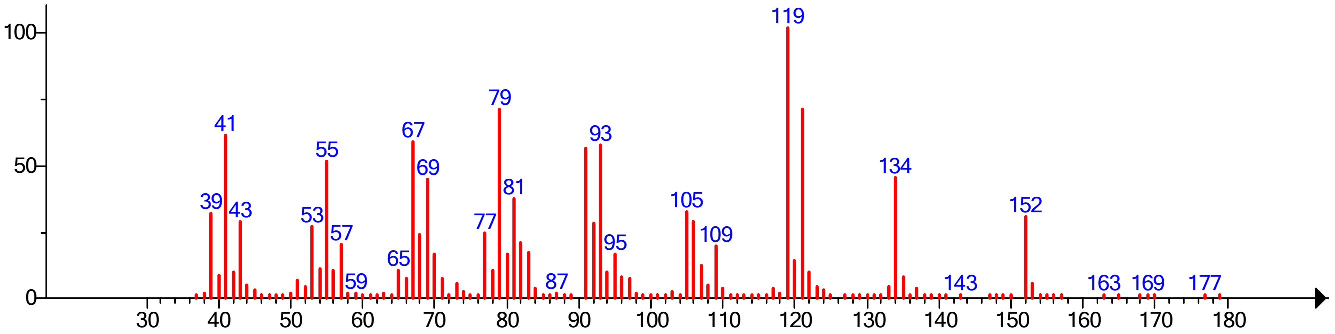

Fig. S14 EI-MS (70 eV) of 2-(hydroxymethyl)-5-(prop-1-en-2-yl)cyclohexan-1-ol (**S2-1**).

S2-2. GC linear retention index on HP-5MS: 1512. EI-MS (70 eV): *m/z* (%) = 170 ($[M]^+$, <1), 121 (100), 67 (76), 69 (73), 79 (73), 41 (72), 55 (66), 81 (59), 93 (52), 119 (45), 91 (41).

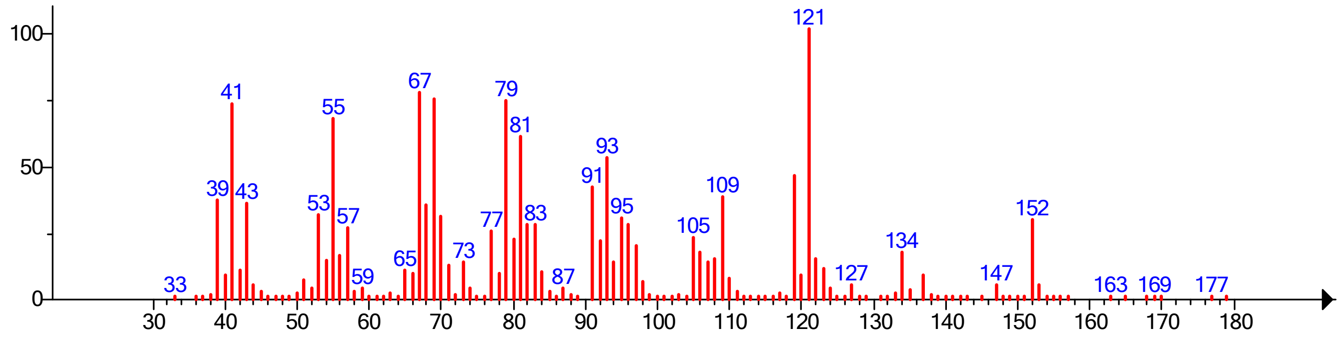

Fig. S15 EI-MS (70 eV) of 2-(hydroxymethyl)-5-(prop-1-en-2-yl)cyclohexan-1-ol (**S2-2**).

S2-3. GC linear retention index on HP-5MS: 1530. EI-MS (70 eV): *m/z* (%) = 170 ($[M]^+$, 2), 79 (100), 69 (95), 67 (90), 41 (88), 55 (80), 81 (75), 93 (75), 119 (60), 121 (58), 91 (52).

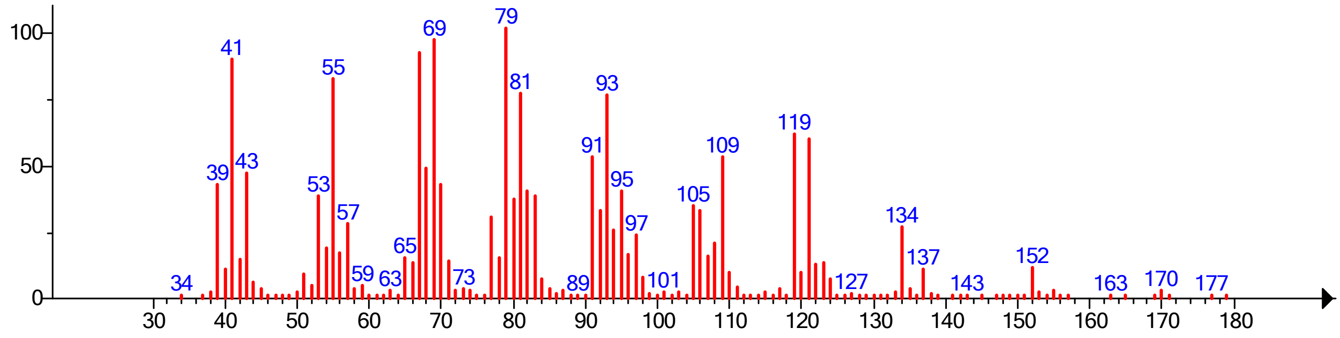

Fig. S16 EI-MS (70 eV) of 2-(hydroxymethyl)-5-(prop-1-en-2-yl)cyclohexan-1-ol (**S2-3**).

1-(hydroxymethyl)-4-(propan-2-ylidene)cyclohexan-1-ol (**S3**). GC linear retention index on HP-5MS: 1451. EI-MS (70 eV): *m/z* (%) = 170 ($[M]^+$, 2), 121 (100), 43 (28), 93 (21), 79 (21), 152 (21), 81 (19), 67 (19), 41 (18), 55 (18), 139 (13).

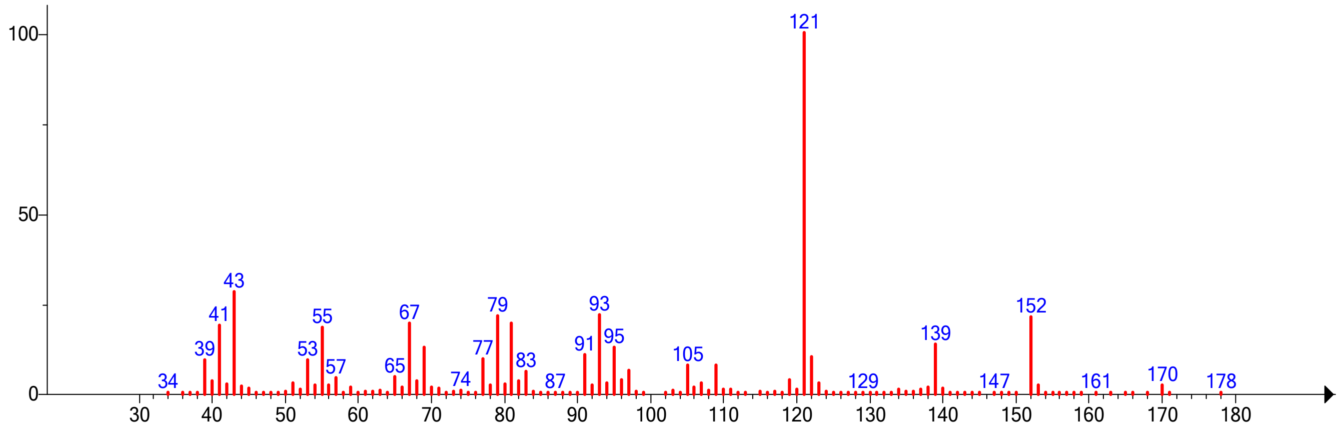

Fig. S17 EI-MS (70 eV) of 1-(hydroxymethyl)-4-(propan-2-ylidene)cyclohexan-1-ol (**S3**).

(*E*)-2-(4-methylpent-3-en-1-yl)but-2-ene-1,4-diol (α-(*E*)-acaridiol, (*E*)-**S4**). GC linear retention index on HP-5MS: 1490. EI-MS (70 eV): *m/z* (%) = 170 ($[M]^+$, <1), 69 (100), 41 (60), 55 (19), 67 (17), 83 (16), 39 (14), 81 (14), 70 (12), 53 (12), 109 (11).

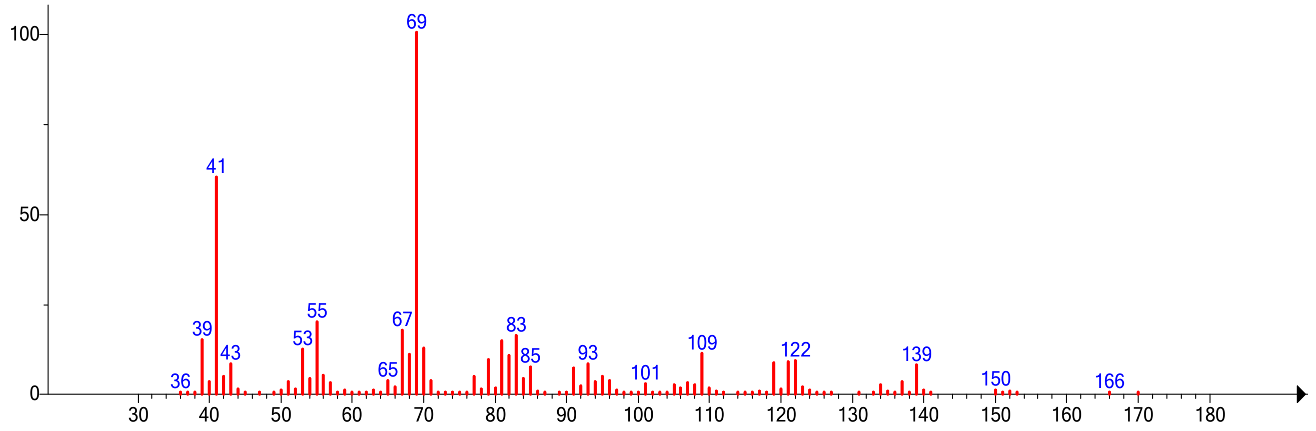

Fig. S18 EI-MS (70 eV) of (*E*)-2-(4-methylpent-3-en-1-yl)but-2-ene-1,4-diol (α-(*E*)-acaridiol, (*E*)-**S4**).

Chiral GC analyses

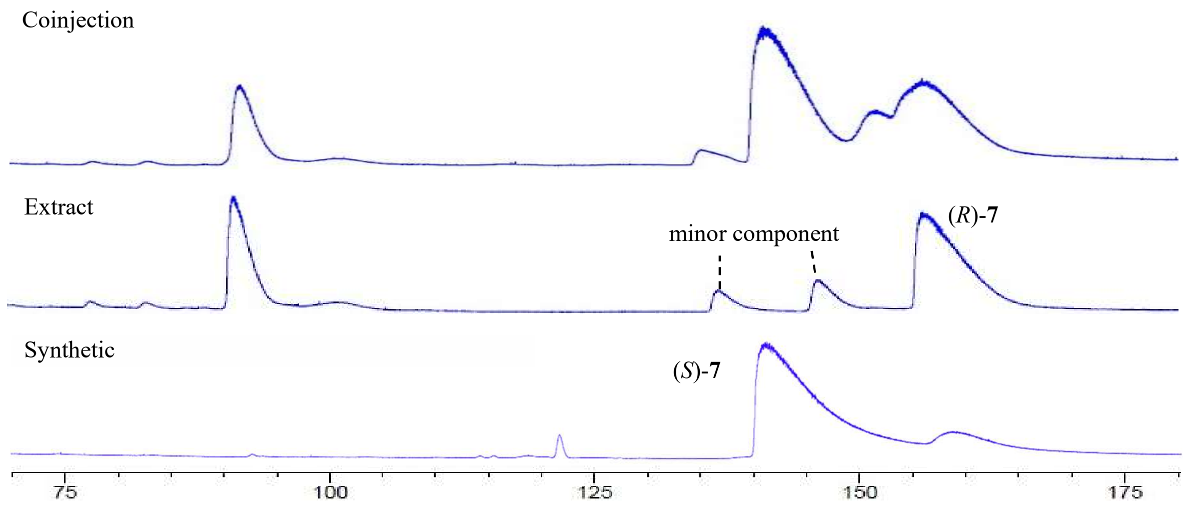

Fig. S19 Chiral GC Comparison between synthetic (*S*)-**7** and rectal gland extracts from male *B. umbrosa* using a HYDRODEX β-6TBDM phase (Macherey & Nagel). The temperature program started at 50 °C, increased at 5 °C/min to 80 °C, held for 180 min, and then ramped at 20 °C/min to the final temperature of 320 °C.

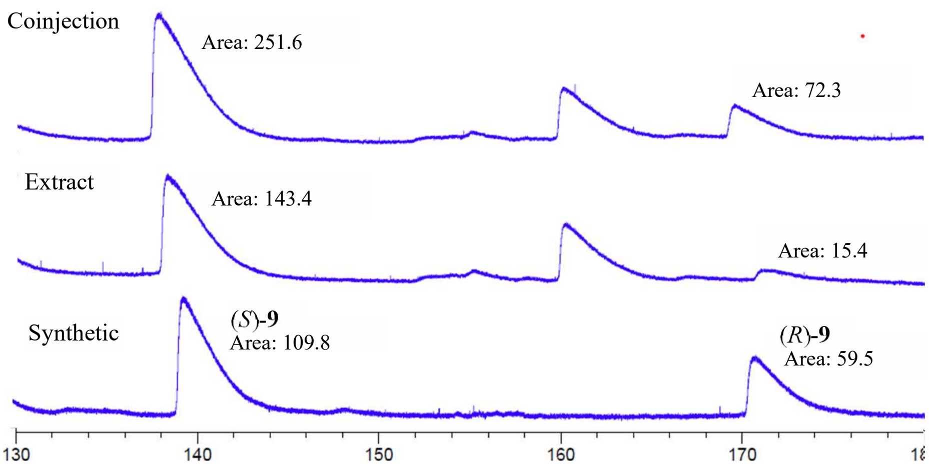

Fig. S20 Chiral GC Comparison between synthetic (*S*)-**9** and rectal gland extracts from male *B. umbrosa* using a Beta-Dex^{TM} 225 chiral capillary column (SUPELCO). The temperature program started at 100 °C, was held for 120 min, and then ramped at 20 °C/min to the final temperature of 250 °C.

3. Quantificaiton

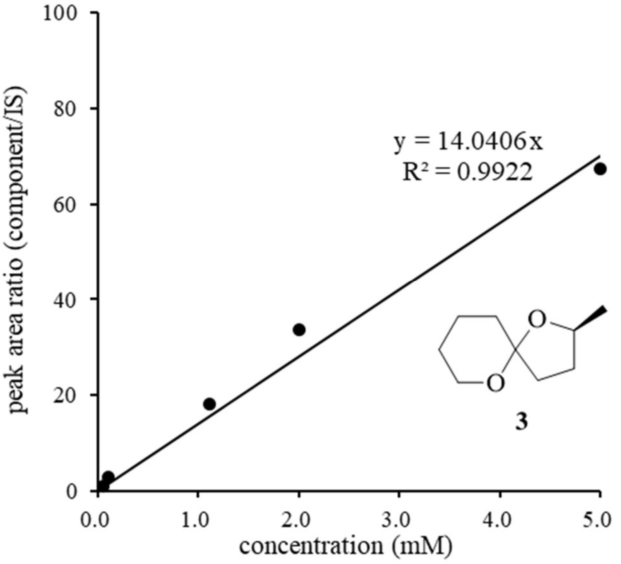

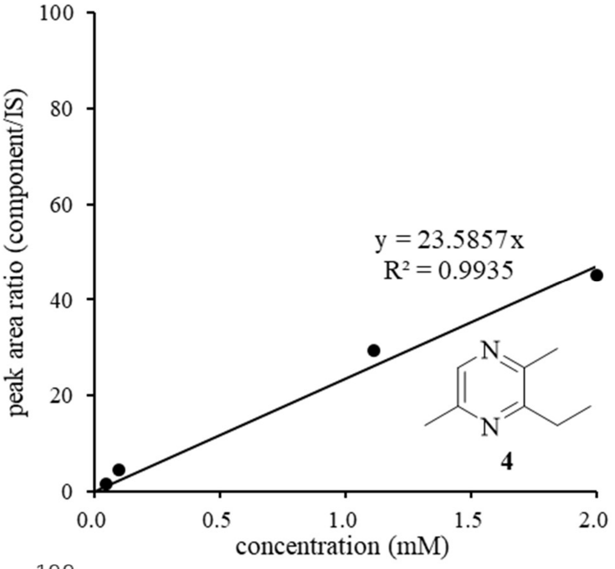

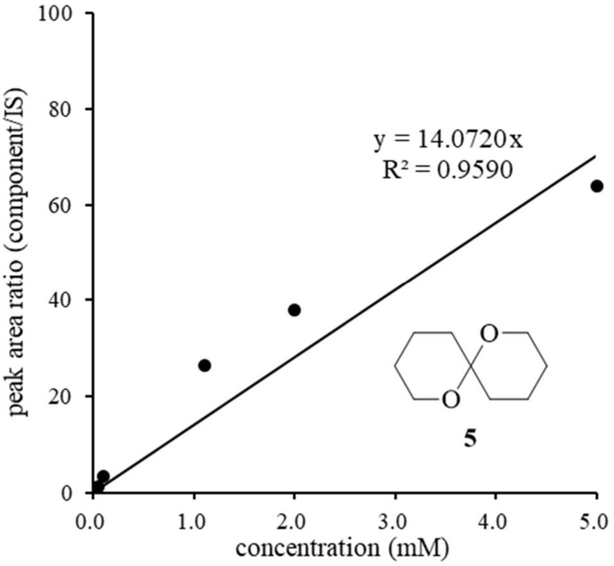

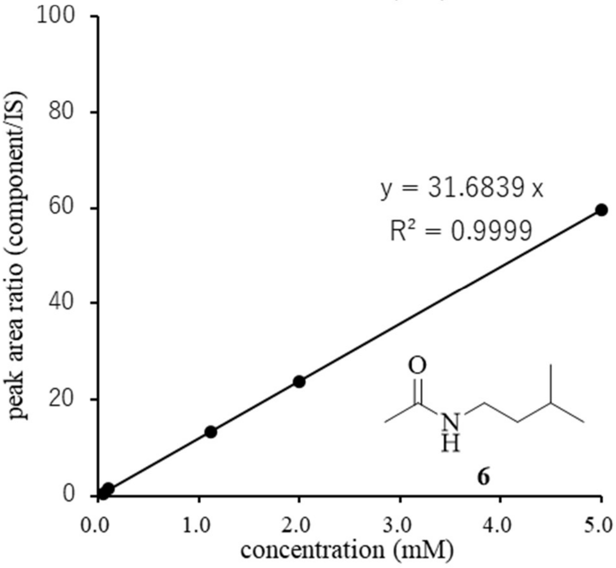

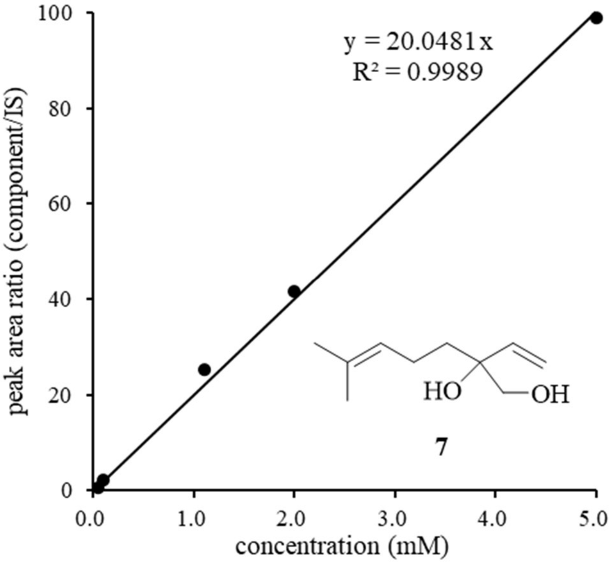

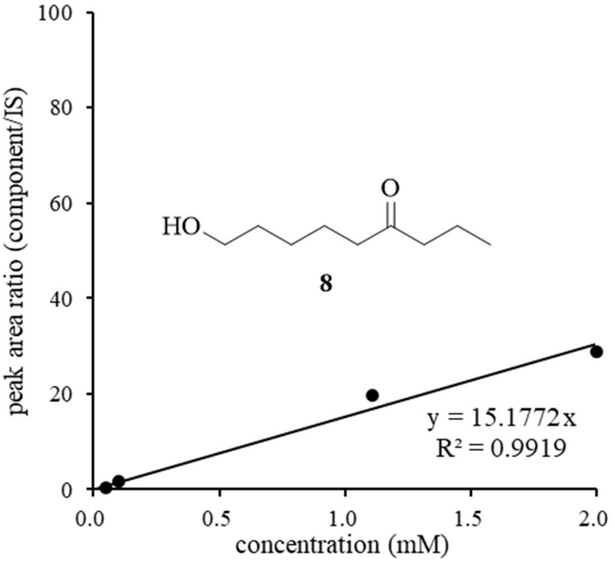

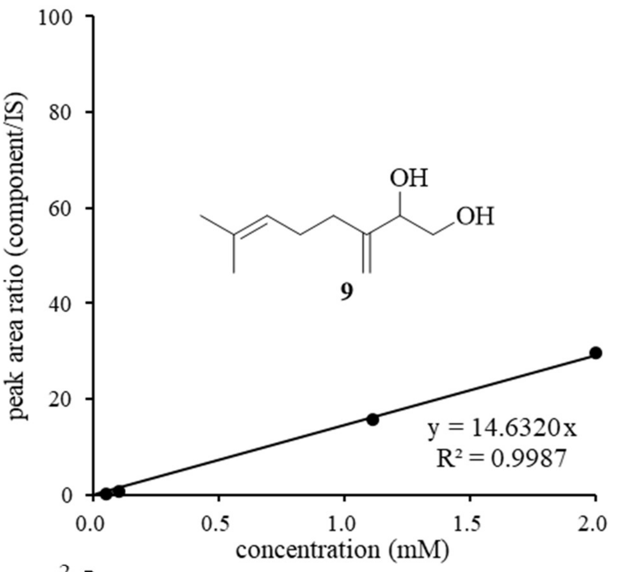

peak area ratio (component/IS)
y = 14.6320x
R² = 0.9987
OH
OH
9
concentration (mM)

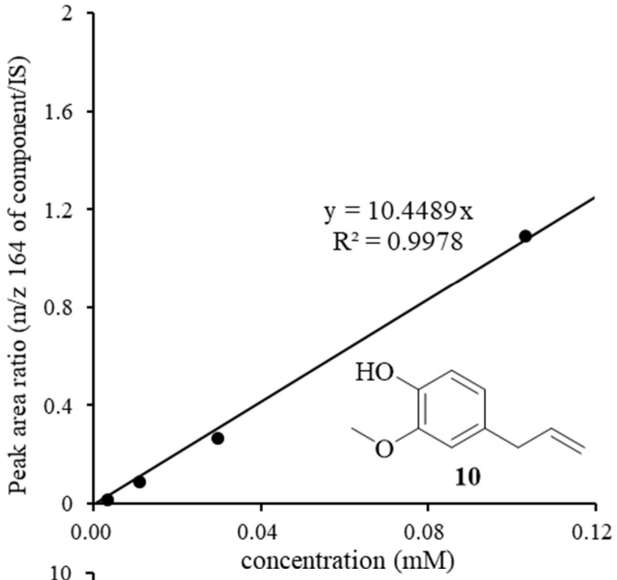

Peak area ratio (m/z 164 of component/IS)
y = 10.4489x
R² = 0.9978
HO
O
10
concentration (mM)

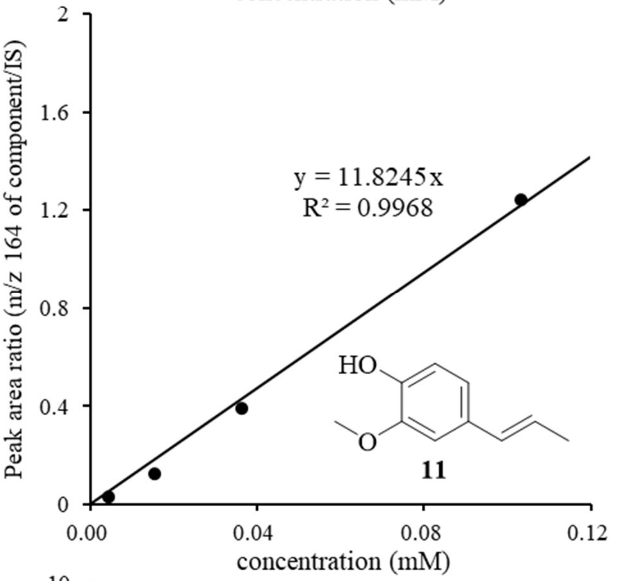

Peak area ratio (m/z 164 of component/IS)
y = 11.8245x
R² = 0.9968
HO
O
11
concentration (mM)

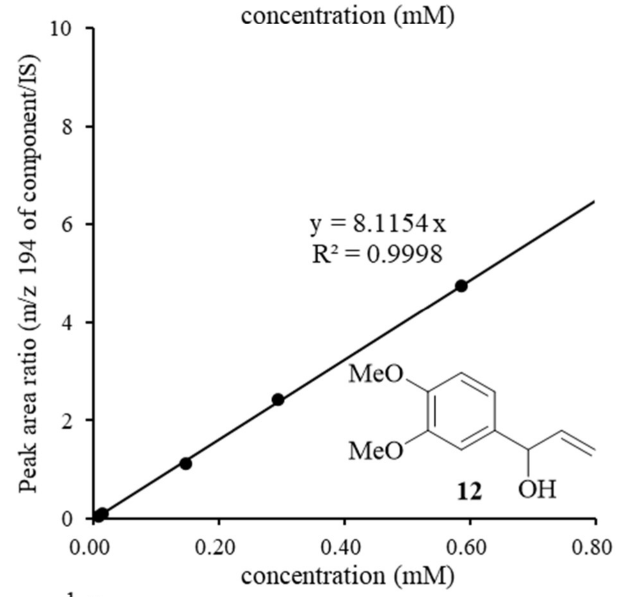

Peak area ratio (m/z 194 of component/IS)
y = 8.1154x
R² = 0.9998
MeO
MeO
OH
12
concentration (mM)

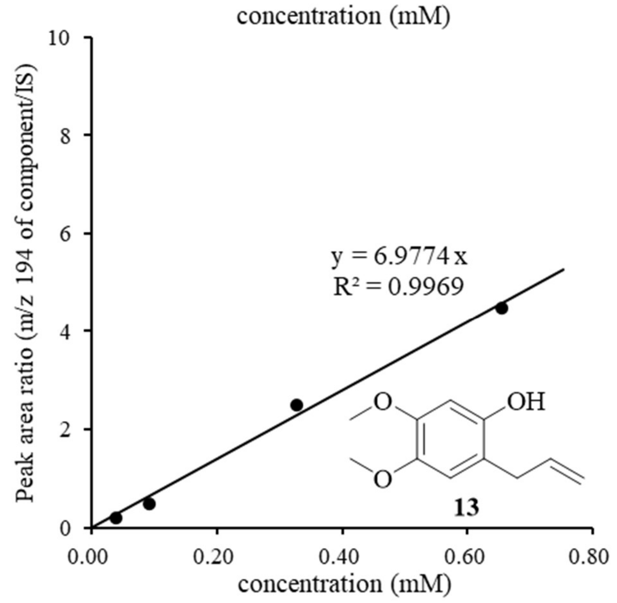

Peak area ratio (m/z 194 of component/IS)
y = 6.9774x
R² = 0.9969
O
OH
O
13
concentration (mM)

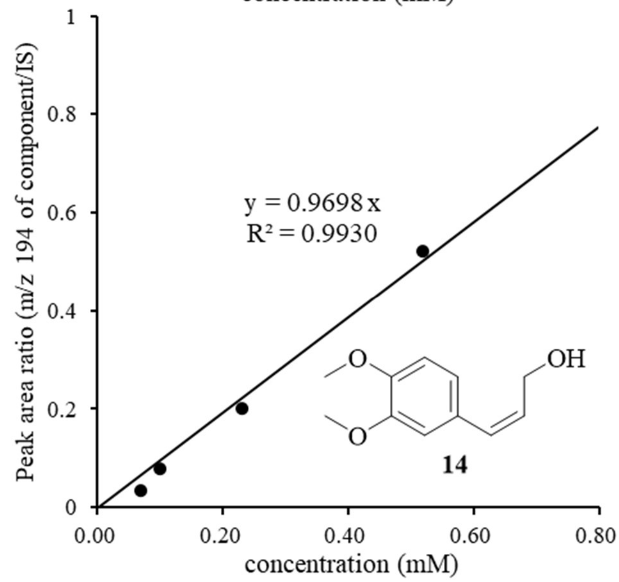

Peak area ratio (m/z 194 of component/IS)
y = 0.9698x
R² = 0.9930
O
OH
O
14
concentration (mM)

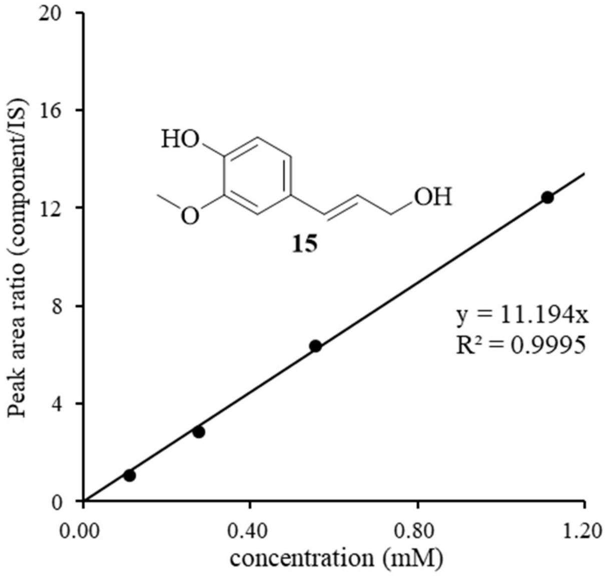

Fig. S21 Calibration curves showing the relationship between peak area ratios (components/internal standard) and concentration. 1-Decanol (1.0 mM) was used as the internal standard. For compounds that co-eluted with other components, quantification was performed using peak areas of specific m/z ions, normalized to the internal standard.

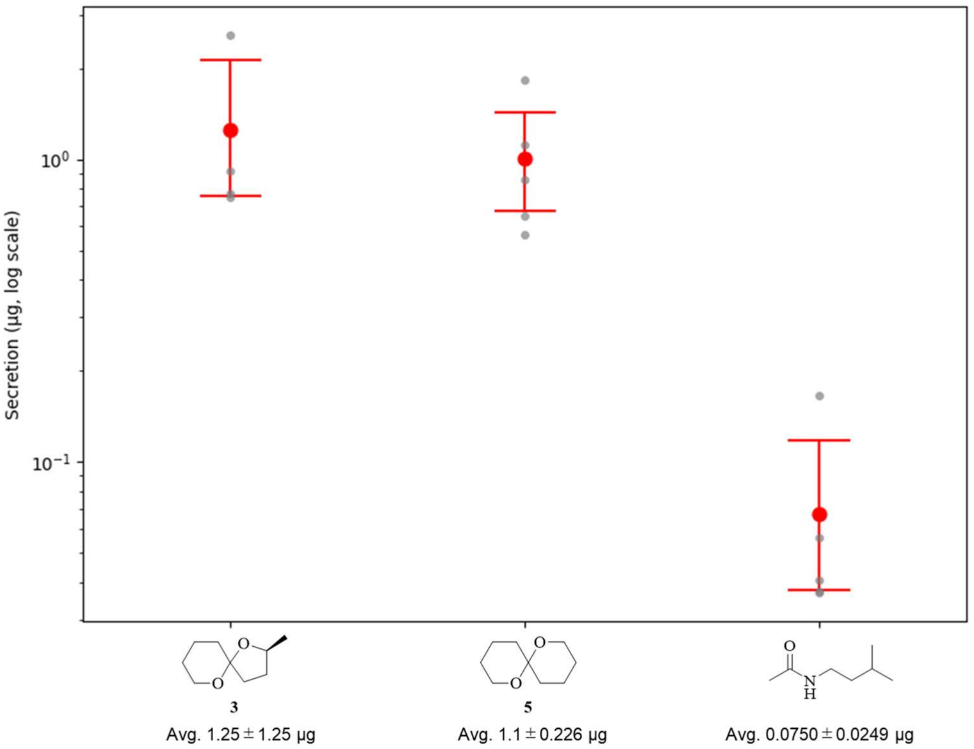

Fig. S22 Quantification of endogenous compounds from single rectal glands of a 6-DAE male *B. umbrosa* (n = 5).

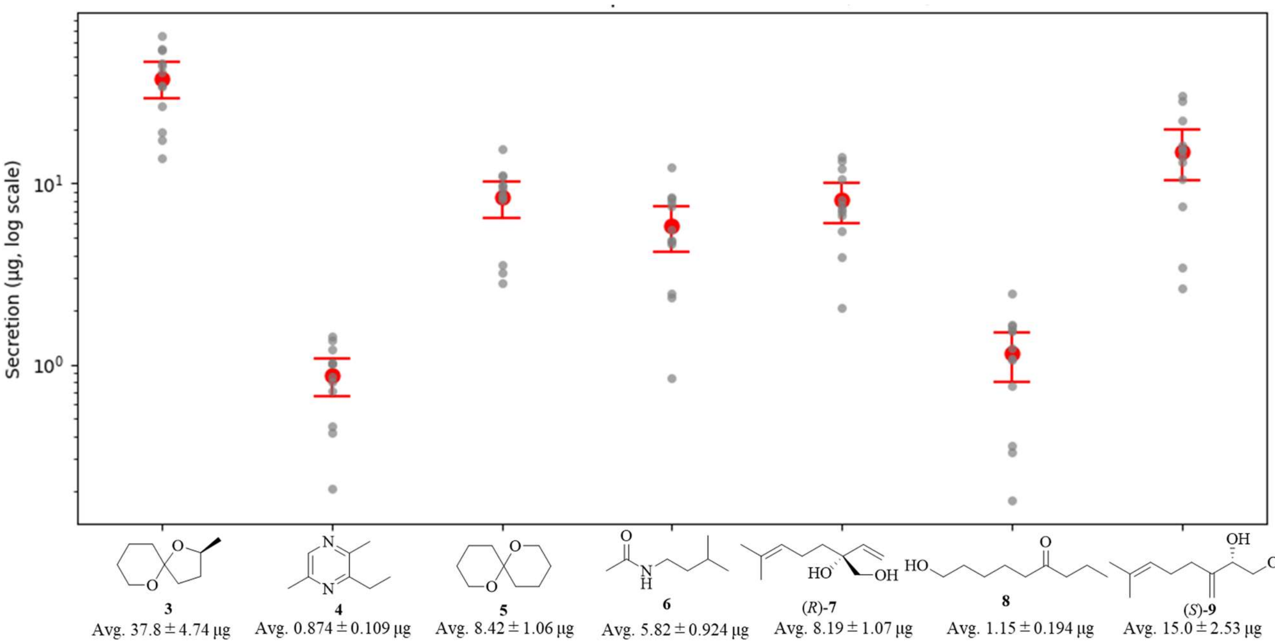

Fig. S23 Quantification of endogenous compounds from single rectal glands of a 20-DAE male *B. umbrosa* (n = 13).

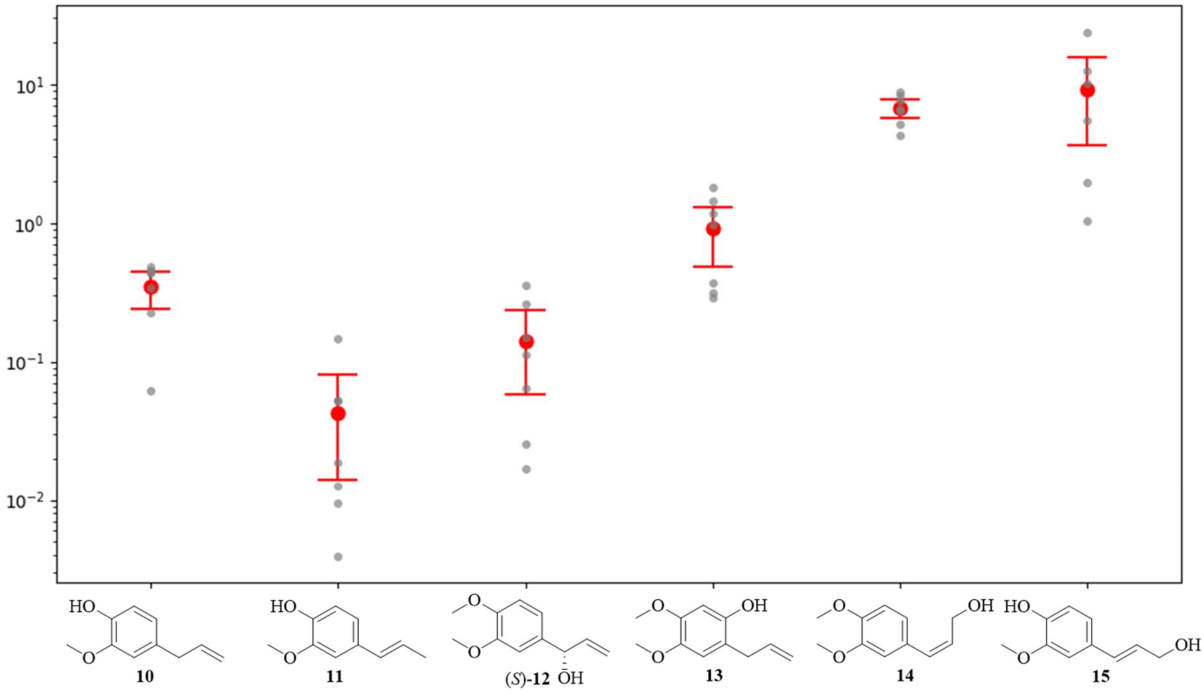

Fig. S24 Quantification of endogenous compounds from single rectal glands of a ME-fed 20-DAE male *B. umbrosa* (n = 7).

4 Experimental Procedures

General remarks on synthesis and analysis

Commercially available starting materials and solvents were used without further purification unless stated otherwise. Technical-grade solvents were distilled before use. All aqueous solutions were saturated unless specified otherwise. All reactions involving water-sensitive reagents were carried out in dried glassware with dry solvents and magnetic stirring under a nitrogen atmosphere. TLC was performed on Polygram® SIL G/UV$_{254}$ plates (Macherey & Nagel) with detection by immersion in 10% w/v phosphomolybdic acid in ethanol, followed by heating. Flash chromatography was performed on silica gel M60 (0.04–0.063 mm, 230–400 mesh ASTM) under pressure. NMR spectra were obtained with the following instruments: Avance II 300 (^{1}H 300 MHz, ^{13}C 75.5 MHz), Avance III HD 300 N (^{1}H 300 MHz, ^{13}C 75.5 MHz), Bruker DRX-400 (^{1}H 400 MHz, ^{13}C 101 MHz), or AV II-600 (^{1}H 600 MHz, ^{13}C 151 MHz). Chemical shifts were reported in ppm relative to tetramethylsilane as an internal standard (δ = 0). Multiplicities of the protons are described as singlets (s), doublets (d), triplets (t), quartets (q), quintets (quint), sextets (sext), septets (sept), or multiplets (m). GC–MS analyses were performed using an Agilent Technologies 8860 gas chromatograph connected to an Agilent Technologies 5977B Series MSD. Mass spectrometry was performed in electron ionization mode (EI) with 70 eV. An HP-5 MS column (Agilent Technologies, 30 m length, 0.25 mm diameter, 0.25 µm film thickness) with helium (Westfalen AG, 99.999%) at a flow rate 1.2 mL/min as the carrier gas was used. The temperature program started at 50 °C, held for 5 min, then increased at a rate of 20 °C/min to 320 °C. For chiral compounds, enantiomeric compositions were determined by chiral GC using HYDRODEX β-6TBDM (Macherey & Nagel, 30 m × 0.25 mm i.d., film thickness 0.40 µm) and Beta-Dex™ 225 (SUPELCO, 30 m × 0.25 mm i.d., film thickness 0.25 µm) chiral stationary phase columns (Fig. S19, S20, and S25). Oven temperature programs varied depending on the compound analyzed and are specified in the corresponding figure legends or experimental sections. IR spectra were recorded on a Bruker Tensor 27 (diamond ATR) instrument or a Dani Instruments DiscovIR detector coupled to an Agilent Technologies 7890B gas chromatograph. The column and temperature programs were identical to those used in the GC/MS analyses mentioned above. An Exactive GC orbitrap mass spectrometer (Thermo Fisher Scientific, Bremen, Germany) was used for high-resolution MS. For chemical ionization in positive mode (CIP), methane (99.995 %) was used as CI-gas at a flow rate of 1.5 mL/min. For electrospray ionization (ESI) analyses, an *LTQ Orbitrap Velos* mass spectrometer (Thermo Fisher Scientific) operated in positive ion mode was used. Spectra were acquired in direct infusion mode with a spray voltage of 2.3–2.8 kV. The tetradecyltrimethylammonium bromide cation (*m/z* 256.29988) served as an internal standard. The scan range was *m/z* 130–2000, with an acquisition time of 1.6 s and a resolution of 100,000 FWHM at *m/z* 400.

Synthesis of (2*S*, 5*SR*)-2-methyl-1,6-dioxaspiro[4.5]decane (**3**)

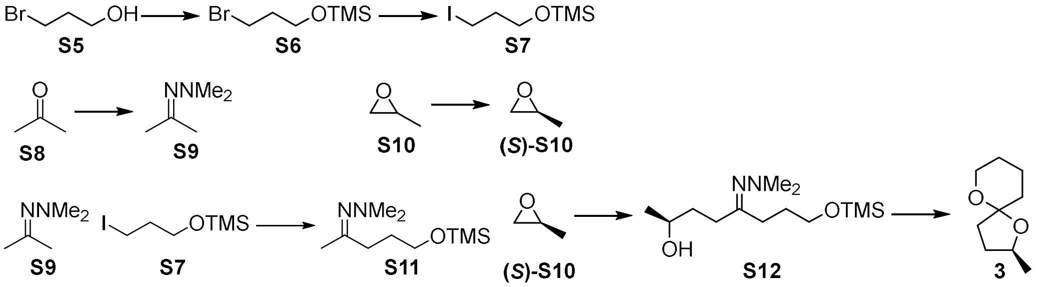

Preparation of (3-bromopropoxy)trimethylsilane (S6)

According to a procedure of Perkins et al. [1], trimethylsilyl chloride (30.0 g, 276 mmol, 1.4 equiv) was added to a stirred solution of 3-bromopropan-1-ol (25.6 g, 184 mmol, 1.0 equiv) and 2,6-lutidine (21.7 g, 276 mmol, 1.5 equiv) in carbon tetrachloride (335 mL) at 0 °C. The resulting mixture was allowed to warm to room temperature and stirred for 3 h. The mixture was filtered through a pad of basic alumina and Celite (1:1) to remove precipitates, washed with Et_2O (200 mL), and the filtrate was concentrated under reduced pressure. The residue was purified by distillation (65 °C at 600 mbar to 70 °C at 20 mbar) to afford (3-bromopropoxy)trimethylsilane as a colorless liquid (34.6 g, 164 mmol, 89%). ^{1}H NMR (300 MHz, $CDCl_3$): δ (ppm) = 3.71 (t, J = 5.8 Hz, 2H), 3.50 (t, J = 6.5 Hz, 2H), 2.04 (tt, J = 6.5, 5.8 Hz, 2H), 0.12 (s, 9H). ^{13}C NMR, DEPT (75.5 MHz, $CDCl_3$): δ (ppm) = 60.01 (CH_2), 35.50 (CH_2), 30.72 (CH_2), –0.40 (3C, CH_3). EI-MS (70 eV): *m/z* (%) = 139 (100), 137 (96), 73 (73), 169 (62), 115 (60), 167 (60), 197 (55), 195 (55), 45 (32), 103 (22). The characterization data for this compound were consistent with the reported data [2].

Preparation of (3-iodopropoxy)trimethylsilane (S7)

According to a procedure of Perkins et al. [1], (3-bromopropoxy)trimethylsilane (34.6 g, 164 mmol) was added to a stirred solution of sodium iodide (30.4 g, 203 mmol, 1.1 equiv) in anhydrous acetone (184 mL) at 0 °C. The resulting mixture was allowed to warm to room temperature and stirred for 3 h. The mixture was filtered through a pad of basic alumina and Celite (1:1) to remove precipitates, washed with Et_2O (100 mL), and the filtrate was concentrated under reduced pressure. The residue was purified by distillation (60 °C at 300 mbar to 80 °C at 1.0 mbar) to afford (3-iodopropoxy)trimethylsilane as a colorless liquid (41.4 g, 160 mmol, 98%). R_f = 0.22 (pentane/ Et_2O 2:1). ^{1}H NMR (300 MHz, $CDCl_3$): δ (ppm) = 3.64 (t, J = 5.8 Hz, 2H), 3.27 (t, J = 6.7 Hz, 2H), 2.09–1.90 (m, 2H), 0.13 (s, 9H). ^{13}C NMR, DEPT (75.5 MHz, $CDCl_3$): δ (ppm) = 62.09 (CH_2), 36.23 (CH_2), 3.77 (CH_2), –20.24 (3C, CH_3). EI-MS (70 eV): *m/z* (%) = 243 (100), 185 (89), 73 (69), 215 (49), 115 (48), 45 (24), 103 (22), 59 (17), 43 (16), 75 (15). The characterization data for this compound were consistent with the reported data [1].

Preparation of 1,1-dimethyl-2-(propan-2-ylidene)hydrazine (S9)

According to a procedure of Géant et al. [3], 1,1-dimethylhydrazine (24.0 g, 400 mmol, 1.00 equiv) (and magnesium sulfate (24.2 g) were added to anhydrous acetone (24.2 g, 416 mmol, 1.04 equiv), and the resulting mixture was heated to reflux for 15 h. The reaction mixture was cooled to room temperature, filtered and concentrated carefully under reduced pressure to afforded 1,1-dimethyl-2-(propan-2-ylidene)hydrazine as a colorless liquid (20.0 g, 200 mmol, 50%). R_f = 0.22 (pentane/ Et_2O 2:1). ^{1}H NMR (300 MHz, $CDCl_3$): δ (ppm) = 2.41 (s, 6H), 1.95 (s, 3H), 1.91 (s, 3H). ^{13}C NMR, DEPT (75.5 MHz, $CDCl_3$): δ (ppm) = 165.15 (C_q), 47.16 (2C, CH_3), 25.29 (CH_3), 18.25 (CH_3). The characterization data for this compound were consistent with the reported data [3].

Preparation of (*S*)-2-methyloxirane ((*S*)-S10)

According to a procedure of Schaus et al. [4], acetic acid (1.32 g, 22.0 mmol, 2.2 mol%) was added to a stirred solution of 2-methyloxirane (58.0 g, 1000 mmol, 1.0 equiv) and (1*S*,2*S*)-(+)-1,2-cyclohexanediamino-*N*,*N*'-bis(3,5-di-t-butylsalicylidene)cobalt(III) ((*S*,*S*)-(salen)Co(OAc)) complex (3.00 g, 5.00 mmol, 0.5 mol%) in H_2O (9.91 g, 550 mmol, 0.55 equiv) at room temperature, and the resulting mixture was stirred for 15 h. The reaction mixture was purified by distillation (38–70 °C) to afford (*S*)-2-methyloxirane as a colorless liquid (24.8 g, 428 mmol, 43%). R_f = 0.22 (pentane/ Et_2O 2:1). $[\alpha]_D^{25}$ = –9.52 (neat) (lit. [9] $[\alpha]_D^{23}$ = –11.6 (neat)). ^{1}H NMR (300 MHz, $CDCl_3$): δ (ppm) = 2.98 (qdd, J = 5.2, 4.5, 2.7 Hz, 1H), 2.74 (dd, J = 4.5 Hz, 1H), 2.42 (dd, J = 5.0, 2.7 Hz, 1H), 1.30 (d, J = 5.2 Hz, 3H). ^{13}C NMR, DEPT (75.5 MHz, $CDCl_3$): δ (ppm) = 48.40 (CH), 48.18 (CH_2), 18.13 (CH_3). The characterization data for this compound were consistent with the reported data [4].

Preparation of 1,1-dimethyl-2-(5-((trimethylsilyl)oxy)pentan-2-ylidene)hydrazine (S11)

Following a modified procedure of Perkins et al. [1], ^{n}BuLi (1.6 M in hexane, 201 mL, 321 mmol, 2.0 equiv) was added dropwise to a stirred solution of 1,1-dimethyl-2-(propan-2-ylidene)hydrazine (20.9 g, 209 mmol, 1.3 equiv) in anhydrous THF (200 mL) at 0 °C. The resulting mixture was stirred for 30 min at the same temperature, allowed to warm to room temperature, and stirred for an additional 10 min. This mixture was cooled again to 0 °C, and (3-iodopropoxy)trimethylsilane (41.4 g, 160 mmol, 1.0 equiv) was added to the reaction mixture. The resulting mixture was stirred for 1 h at the same temperature, allowed to warm to room temperature and stirred for 15 h. This mixture was filtered through a pad of basic alumina and Celite (1:1) to remove precipitates, washed with ^{n}Pn/Et_2O (1:1, 200 mL), and the filtrate was concentrated under reduced pressure. The residue was purified by distillation (70 °C at 100 mbar to 120 °C at 1.0 mbar) to afford the crude of 1,1-dimethyl-2-(5-((trimethylsilyl)oxy)pentan-2-ylidene)hydrazine as a colorless liquid (18.8 g, 81.2 mmol).

Preparation of (2*S*, 5*SR*)-2-methyl-1,6-dioxaspiro[4.5]decane (3)

Following a modified procedure of Perkins et al. [1], LDA was prepared from ^{i}Pr$_2$NH (13.4 g, 132 mmol, 1.6 equiv) and ^{n}BuLi (1.6 M in hexane, 93 mL, 149 mmol, 1.8 equiv) in anhydrous THF (250 mL) at –78 °C under a nitrogen atmosphere. 1,1-dimethyl-2-(5-((trimethylsilyl)oxy)pentan-2-ylidene)hydrazine (18.8 g, 81.2 mmol, 1.0 equiv) in anhydrous THF (140 mL) was added to the stirred solution of the freshly prepared LDA at –78 °C, and the resulting mixture was allowed to warm to 0 °C and stirred for 3 h. The reaction mixture was cooled again to –78 °C, and (*S*)-2-methyloxirane (14.1 g, 244 mmol, 3.0 equiv) was added dropwise to the mixture. The mixture was stirred for 15 h and then allowed to warm to room temperature. At the same temperature, acetic acid (16.1 g, 244 mmol, 3.0 equiv) was added to the stirred mixture, followed by magnesium sulfate (131 g) and Amberlite IR120 (99.0 g). The resulting mixture was heated to reflux for 2 h, filtered through a paper filter, and washed with THF (50 mL). The filtrate was concentrated under reduced pressure, and the residue was purified by distillation (75 °C at 500 mbar to 110 °C at 1.0 mbar) to afford the mixture of (2*S*, 5*SR*)-2-methyl-1,6-dioxaspiro[4.5]decane as a colorless liquid (5.76 g, 36.9 mmol, 23% over 3 steps, 85% *ee*). R_f = 0.22 (DCM/MeOH 95:5). ^{1}H NMR (300 MHz, $CDCl_3$): δ (ppm) = 4.30–4.10 (m, 2H), 3.98–3.79 (m, 2H), 3.68–3.49 (m, 2H), 2.21–1.75 (m, 8H), 1.75–1.54 (m,8H), 1.53–1.33 (m, 4H), 1.30 (d, J = 6.2 Hz, 3H), 1.21 (d, J = 5.8 Hz, 3H). ^{13}C NMR, DEPT (75.5 MHz, $CDCl_3$): δ (ppm) = 105.95 (C_q), 105.73 (C_q), 76.85 (CH), 74.11 (CH), 61.70 (CH_2), 61.56 (CH_2), 39.11 (CH_2), 37.88 (CH_2), 34.32 (CH_2), 34.18 (CH_2), 31.82 (CH_2), 31.51 (CH_2), 25.50 (CH_2), 25.41 (CH_2), 23.31 (CH_2), 21.39 (CH_3), 20.47 (CH_3), 20.41 (CH_2). EI-MS (R_I = 1007, 70 eV): *m/z* (%) = 156 (4), 101 (100), 55 (73), 43 (59), 98 (55), 41 (54), 83 (50), 56 (43), 39 (35), 100 (28), 42 (25). EI-MS (R_I = 1016, 70 eV): *m/z* (%) = 156 (4), 101 (100), 55 (64), 98 (48), 41 (46), 83 (46), 43 (45), 56 (38), 39 (29), 100 (29), 42 (21). The enantiomer composition was determined by GC on a HYDRODEX β-6TBDM phase (Macherey & Nagel). The temperature program started at 50 °C, increased at 0.5 °C/min to 70 °C, held for 10 min, and then ramped at 40 °C/min to the final temperature of 230 °C.The characterization data for this compound were consistent with the reported data [1].

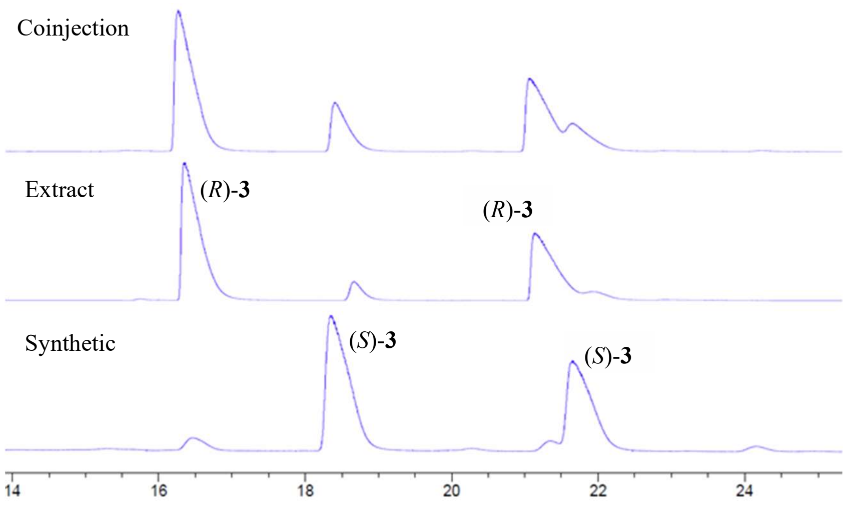

Fig. S25 Chiral GC comparison between synthetic (*S*)-**3** and rectal gland extracts from male *B. umbrosa* using a HYDRODEX β-6TBDM phase.

Synthesis of 3-ethyl-2,5-dimethylpyrazine (4)

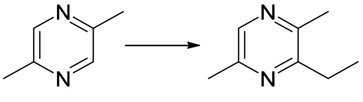

S13 **4**

According to a procedure of Nicoli et al. [5], ethylmagnesium bromide was prepared from ethyl bromide (12.0 g, 109 mmol, 1.2 equiv) and magnesium (2.67 g, 109 mmol, 1.2 equiv) in Et_2O (84 mL) and cooled in an ice bath. The mixture of 2,5-dimethylpyrazine (12.0 g, 84.2 mmol, 1.0 equiv) and dichloro(1,3-bis(diphenylphosphino)propane)nickel (456 mg, 0.842 mmol, 1.0 mol%) in Et_2O (320 mL) was added to the stirred solution of the freshly prepared Grignard reagent. The mixture was allowed to warm to room temperature over 1 h and stirred for an additional 14 h. The reaction was quenched with water (100 mL). The phases were separated, and the aqueous phase was extracted with Et_2O (3 × 40 mL). The combined organic phases were washed with saturated NaCl solution (50 mL) and dried over $MgSO_4$, filtered, and the solvent was removed under reduced pressure. The crude product was purified by column chromatography (SiO_2, pentane/Et_2O 100:0 to 80:20) to afford 3-ethyl-2,5-dimethylpyrazine as a yellow oil (6.17 g, 45.3 mmol, 54%). R_f = 0.42 (pentane/Et_2O 1:3). ^1H NMR (300 MHz, $CDCl_3$): δ (ppm) = 8.14 (s, 1H), 2.79 (q, J = 7.6 Hz, 2H,), 2.51 (s, 3H), 2.48 (s, 3H), 1.26 (t, J = 7.6 Hz, 3H), ^{13}C NMR (75.5 MHz, $CDCl_3$): δ (ppm) =155.85 (C_{Ar}), 150.22 (C_{Ar}), 148.48 (C_{Ar}), 140.8 (CH_{Ar}), 28.41 (CH_2), 21.21 (2C, CH_3), 12.76 (CH_3). EI-MS (70 eV): *m/z* (%) = 136 ([M]$^+$, 75), 135 (100), 42 (20), 108 (15), 39 (15), 107 (13), 56 (11), 121 (6), 137 (6), 40 (5). The characterization data for this compound were consistent with the reported data [5].

Synthesis of 1,7-dioxaspiro[5.5]undecane (5)

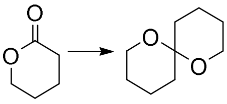

S14 **5**

According to a procedure of Nabi et al. [6], titanium tetrachloride (246 g, 1.30 mol, 1.3 equiv) was added dropwise to a stirred solution of δ-valerolactone (100 g, 0.999 mol, 1.0 equiv) and triethylamine (152 g, 1.50 mol, 1.5 equiv) in DCM (500 mL) at –78 °C. The resulting mixture was allowed to warm to room temperature and stirred for 2 h. This mixture was poured into ice water (1.0 L), and the phases were separated. The aqueous layer was extracted with DCM (3 × 500 mL), and the combined organic layer was dried over $MgSO_4$, filtered, and concentrated under reduced pressure. The residue was dissolved in anhydrous THF (200 mL), and 1 M NaOH solution was added to the mixture in an ice bath to adjust the pH to 12. The resulting mixture was heated to 60 °C and stirred for 2 h. 1 M HCl solution was added to the reaction to adjust the pH to 5.0. The resulting mixture was extracted with DCM (3 × 100 mL), and the combined organic phases were dried over $MgSO_4$, filtered, and concentrated under reduced pressure. The residue was purified by neutral Al_2O_3 column chromatography (pentane/Et_2O 100:0 to 97:3) to afford 1,7-dioxaspiro[5.5]undecane as a colorless liquid (22.0 g, 0.140 mol, 28%). R_f = 0.42 (DCM/MeOH 95:5). ^1H NMR (300 MHz, $CDCl_3$): δ (ppm) = 3.77–3.46 (m, 4H), 1.96–1.68 (m, 2H), 1.68–1.39 (m, 10H). ^{13}C NMR, DEPT (75.5 MHz, $CDCl_3$): δ (ppm) = 94.98 (C_q), 60.35 (CH_2), 35.73 (CH_2), 25.30 (CH_2), 18.52 (CH_2). EI-MS (70 eV): *m/z* (%) = 156 ([M]$^+$, 8), 98 (100), 101 (98), 55 (60), 100 (55), 41 (41), 83 (34), 43 (28), 56 (25), 42 (19), 39 (18). The characterization data for this compound were consistent with the reported data [6].

Synthesis of *N*-isopentylacetamide (**6**)

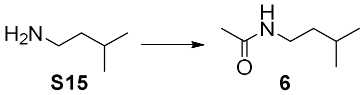

S15 **6**

Following a modified procedure of Milan et al. [7], triethylamine (2.32 g, 22.9 mmol, 1.1 equiv) and acyl chloride (1.63 g, 20.8 mmol, 1.0 equiv) were added to a stirred solution of 3-methylbutan-1-amine (2.00 g, 22.9 mmol, 1.1 equiv) in DCM (100 mL) at 0 °C, and the mixture was stirred at room temperature for 15 h. Then, saturated $NaHCO_3$ solution (30 mL) was added to quench the reaction at 0 °C. The resulting mixture was extracted with DCM (3 × 20 mL). The combined organic phases were washed with 1 M HCl solution (20 mL) and dried over $MgSO_4$, filtered, and the solvent was removed under reduced pressure. The residue was purified by silica gel column chromatography (pentane/Et_2O 9:1 to 4:6) to afford *N*-isopentylacetamide as a colorless liquid (2.67 g, 20.7 mmol, 100%). R_f = 0.29 (DCM/MeOH 95:5). ^1H NMR (300 MHz, $CDCl_3$): δ (ppm) = 5.75 (s, 1H), 3.22 (dt, J = 7.8, 5.8 Hz, 2H), 1.94 (s, 3H), 1.59 (hept, J = 6.7 Hz, 1H), 1.36 (dt, J = 7.8, 6.7 Hz, 1H), 0.88 (d, J = 6.7 Hz, 6H). ^{13}C NMR, DEPT (76 MHz, $CDCl_3$): δ (ppm) = 170.14 (C(O)), 38.54 (CH_2), 38.04 (CH_2), 25.93 (CH), 23.39 (CH_3), 22.53 (2C, CH_3). EI-MS (70 eV): *m/z* (%) = 129 ([M]$^+$, 10), 73 (100), 72 (73), 43 (60), 30 (59), 44 (42), 60 (35), 86 (29), 114 (19), 55 (19), 41 (15). The characterization data for this compound were consistent with the reported data [7].

Synthesis of 6-methyl-2-vinylhept-5-ene-1,2-diol (**7**)

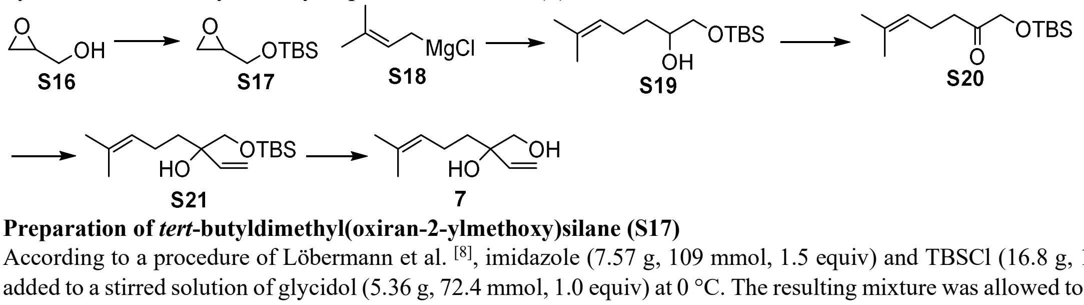

Preparation of *tert*-butyldimethyl(oxiran-2-ylmethoxy)silane (S17)

According to a procedure of Löbermann et al. [8], imidazole (7.57 g, 109 mmol, 1.5 equiv) and TBSCl (16.8 g, 109 mmol, 1.5 equiv) were added to a stirred solution of glycidol (5.36 g, 72.4 mmol, 1.0 equiv) at 0 °C. The resulting mixture was allowed to warm to room temperature and stirred for 16 h. Then, the mixture was filtered through Celite, and the solvent was removed under reduced pressure. The crude product was purified by column chromatography (SiO_2, pentane/Et_2O 100:0 to 98:2) to afford *tert*-butyldimethyl(oxiran-2-ylmethoxy)silane as a

colorless liquid (13.6 g, 72.2 mol, 100%). R_f = 0.59 (pentane/Et_2O 9:1). ^1H NMR (300 MHz, $CDCl_3$): δ (ppm) =3.85 (dd, J = 12.0, 3.1 Hz, 1H), 3.66 (dd, J = 12.0, 4.6 Hz, 1H), 3.16–3.02 (m, 1H), 2.77 (dd, J = 4.8, 4.8 Hz, 1H), 2.64 (dd, J = 4.8, 2.5 Hz, 1H), 0.91 (s, 9H), 0.08 (s, 6H). ^{13}C NMR, DEPT (75.5 MHz, $CDCl_3$): δ (ppm) = 63.90 (CH_2), 52.57 (CH), 44.62 (CH_2), 26.02 (3C, CH_3), 18.52 (C_q), –5.16 (2C, CH_3). EI-MS (70 eV): *m/z* (%) = 188 ([*M*]$^+$,＜1), 101 (100), 59 (31), 75 (20), 131 (19), 73 (10), 102 (9), 45 (7), 41 (5), 57 (5), 103 (4). The characterization data for this compound were consistent with the reported data [8].

Preparation of 1-((*tert*-butyldimethylsilyl)oxy)-6-methylhept-5-en-2-ol (S19)

According to a procedure of Löbermann et al. [8], 8 vol% solution of prenyl chloride (22.6 g, 0.217 mol, 1.0 equiv) in anhydrous THF (50 mL) was carefully added to a mixture of magnesium (7.96 g, 0.325 mol, 1.2 equiv) and a small crystal of iodine in dry THF (217 mL) at room temperature. The mixture was cooled to –15 °C when the reaction started. The remaining prenyl chloride solution was added over 2 h, and the resulting mixture was allowed to warm to room temperature to provide grey suspension containing precipitates. CuI (4.12 g, 21.6 mmol, 10 mol%) was added to a stirred solution of *tert*-butyldimethyl(oxiran-2-ylmethoxy)silane (44.8 g, 0.238 mol, 1.1 equiv) in anhydrous THF (40 mL). The mixture was cooled to –60 °C, and the freshly prepared prenylmagnesium chloride solution was then added over 1 h. The reaction mixture was warmed to –20 °C and stirred for an additional 15 h. Crushed ice was added to quench the reaction, and the mixture was allowed to warm to room temperature. Then, saturated NH_4Cl solution (50 mL) was added, and the aqueous phase was extracted with EtOAc (4 × 50 mL). The combined organic phases were washed with saturated $NaHCO_3$ solution (50 mL) and saturated NaCl solution (50 mL), then dried over $MgSO_4$, filtered, and the solvent was removed under reduced pressure. The crude product was purified by column chromatography (SiO_2, pentane/Et_2O 100:0 to 98:2) to afford 1-((*tert*-butyldimethylsilyl)oxy)-6-methylhept-5-en-2-ol as a colorless liquid (39.9 g, 154 mmol, 71%). R_f = 0.40 (pentane/Et_2O 9:1). ^1H NMR (300 MHz, $CDCl_3$): δ (ppm) = 5.11 (t, J = 7.3 Hz, 1H), 3.69–3.62 (m, 1H), 3.62–3.59 (m, 1H), 3.40 (dd, J = 10.3, 8.0 Hz, 1H), 2.39 (s, OH, 1H), 2.23–1.95 (m, J = 7.3 Hz, 2H), 1.69 (s, 3H), 1.62 (s, 3H), 1.54–1.33 (m, 2H), 0.90 (s, 9H), 0.07 (s, 6H). ^{13}C NMR, DEPT (75.5 MHz, $CDCl_3$): δ (ppm) = 132.23 (C_q), 124.30 (CH), 71.68 (CH), 67.48 (CH_2), 33.13 (CH_2), 26.14 (3C, CH_3), 25.96 (CH_3), 24.38 (CH_2), 18.56 (C_q), 17.92 (CH_3), –5.09 (CH_3), –5.14 (CH_3). EI-MS (70 eV): *m/z* (%) = 258 ([*M*]$^+$,＜1), 75 (100), 109 (67), 67 (59), 69 (49), 73 (37), 41 (36), 105 (22), 201 (17), 55 (16), 81 (14). The characterization data for this compound were consistent with the reported data [8].

Preparation of 1-((*tert*-butyldimethylsilyl)oxy)-6-methylhept-5-en-2-one (S20)

According to a procedure of Löbermann et al. [8], DMSO (40.8 g, 522 mmol, 5.4 equiv) in anhydrous DCM (120 mL) was added dropwise to a stirred solution of oxalyl chloride (31.9 g, 251 mmol, 2.6 equiv) in anhydrous DCM (50 mL) at –78 °C. The resulting mixture was stirred for 15 min at the same temperature, and then 1-((*ter*t-butyldimethylsilyl)oxy)-6-methylhept-5-en-2-ol (25.0 g, 96.7 mmol, 1.0 equiv) was added. After 1 h at –78 °C, triethylamine (97.9 g, 967 mmol, 10 equiv) was added to the reaction mixture, and it was allowed to warm to room temperature over 1 h. Then, saturated $NaHCO_3$ solution (50 mL) was added to quench the reaction, and the phases were separated. The aqueous layer was extracted with DCM (3 × 50 mL), and the combined organic phases were washed with saturated $NaHCO_3$ solution (30 mL) and brine (30 mL). The organic layer was dried over $MgSO_4$ and concentrated under reduced pressure. The residue was purified by silica gel column chromatography (pentane/Et_2O 100:0 to 99:1) to afford 1-((*tert*-butyldimethylsilyl)oxy)-6-methylhept-5-en-2-one as a colorless liquid (24.6 g, 96.5 mmol, 100%). R_f = 0.71 (pentane/Et_2O 9:1). ^1H NMR (300 MHz, $CDCl_3$): δ (ppm) = 5.07 (ddt, J = 8.6, 5.7, 1.4 Hz, 1H), 4.16 (s, 2H), 2.51 (t, J = 7.5 Hz, 2H), 2.38–2.15 (m, 2H), 1.67 (dtd, J = 1.4, 0.4, 0.4 Hz, 3H), 1.61 (dtd, J = 1.4, 0.8, 0.4 Hz, 3H), 0.84 (s, 9H) 0.08 (s, 6H). ^{13}C NMR, DEPT (75.5 MHz, $CDCl_3$): δ (ppm) = 210.98 (C(O)), 132.92 (C_q), 122.91 (CH), 69.57 (CH_2), 38.61 (CH_2), 25.94 (3C, CH_3), 25.83 (CH_3), 22.23 (CH_2), 18.47 (C_q), 17.81 (CH_3), –5.36 (2C, CH_3). EI-MS (70 eV): *m/z* (%) = 256 ([*M*]$^+$,＜1), 69 (100), 199 (58), 41 (37), 73 (35), 107 (29), 115 (28), 75 (25), 129 (20), 131 (19), 59 (10). The characterization data for this compound were consistent with the reported data [8].

Preparation of 3-(((*tert*-butyldimethylsilyl)oxy)methyl)-7-methylocta-1,6-dien-3-ol (S21)

According to a procedure of Löbermann et al. [8], a vinylMgBr solution (1.0 M in THF, 9.36 mL, 9.36 mmol, 1.2 equiv) was added dropwise to a stirred solution of 1-((*tert*-butyldimethylsilyl)oxy)-6-methylhept-5-en-2-one (2.00 g, 7.80 mmol, 1.0 equiv) in THF (35 mL) and Et_2O (19 mL) at –78 °C. After 3 h at the same temperature, the reaction mixture was allowed to warm to 0 °C, and saturated NH_4Cl (30 mL) solution was added to quench the reaction. The phases were separated, and the aqueous layer was extracted with Et_2O (3 × 10 mL). The combined organic phases were dried over $MgSO_4$ and concentrated under reduced pressure to afford 3-(((*tert*-butyldimethylsilyl)oxy)methyl)-7-methylocta-1,6-dien-3-ol as a colorless liquid (1.93 g, 6.80 mmol, 87%). R_f = 0.66 (pentane/Et_2O 9:1). ^1H NMR (300 MHz, $CDCl_3$): δ (ppm) = 5.78 (dd, J = 17.3, 10.8 Hz, 1H), 5.30 (dd, J = 17.3, 1.7 Hz, 1H), 5.16 (dd, J = 10.8, 1.7 Hz, 1H), 5.09 (ddt, J = 8.6, 5.7, 1.4 Hz, 1H), 3.46 (s, 2H), 2.45 (s, OH, 1H), 2.14–1.91 (m, 2H), 1.55–1.67 (overlapped, 1H), 1.69 (d, J = 1.4 Hz, 3H), 1.59 (d, J = 1.4 Hz, 3H), 1.45 (ddd, J = 13.6, 11.0, 5.7 Hz, 1H), 0.89 (s, 9H), 0.05 (s, 3H), 0.05 (s, 3H). ^{13}C NMR, DEPT (75.5 MHz, $CDCl_3$): δ (ppm) = 141.15 (CH), 131.71 (C_q), 124.67 (CH), 114.33 (CH_2), 75.42 (C_q), 69.55 (CH_2), 37.17 (CH_2), 26.00 (3C, CH_3), 25.84 (CH_3), 22.25 (CH_2), 18.44 (C_q), 17.79 (CH_3), –5.26 (CH_3), –5.31 (CH_3). EI-MS (70 eV): *m/z* (%) = 284([*M*]$^+$, ＜1), 75 (100), 69 (88), 73 (67), 93 (48), 41 (44), 107 (31), 135 (28), 55 (27), 89 (26), 79 (24). The characterization data for this compound were consistent with the reported data [8].

Preparation of 6-methyl-2-vinylhept-5-ene-1,2-diol (7)

According to a procedure of Löbermann et al. [8], tetrabutylammonium fluoride (1.0 M in THF, 0.46 mL, 0.458 mmol, 3.0 equiv) was added dropwise to a stirred solution of 9-((tert-butyldiphenylsilyl)oxy)nonan-4-one (44.3 mg, 0.156 mmol, 1.0 equiv) in THF (0.4 mL) at 0 °C. The mixture was allowed to warm to room temperature and stirred for 30 min. Then, saturated $NaHCO_3$ solution (0.5 mL) was added to quench the reaction. The resulting mixture was extracted with EtOAc (3 × 1 mL) , and the combined organic phases were dried over $MgSO_4$, filtered, and concentrated under reduced pressure. The residue was purified by silica gel column chromatography (pentane/Et_2O 1:1 to 1:2) to afford 6-methyl-2-vinylhept-5-ene-1,2-diol as a colorless liquid (21.1 mg, 0.124 mmol, 80%). R_f = 0.48 (pentane/ Et_2O 1:3). ^1H NMR (300 MHz, $CDCl_3$): δ (ppm) = 5.81 (dd, J = 17.4, 10.8 Hz, 1H), 5.47–5.29 (dd, J = 17.4, 1.4 Hz, 1H), 5.27 (dd, J = 10.8, 1.4 Hz, 1H), 5.12 (ddt, J = 8.6, 5.7, 1.4 Hz, 1H), 3.54–3.45 (m, 2H), 2.27 (s, OH, 1H), 2.04 (m, 2H), 1.82 (s, OH, 1H), 1.69–1.61 (overlapped, 1H), 1.68 (s, 3H), 1.60 (s, 3H), 1.51 (ddd, J = 13.7, 10.1, 5.7 Hz, 1H). ^{13}C NMR, DEPT (75.5 MHz, $CDCl_3$): δ (ppm) = 140.92 (CH), 132.56 (C_q), 124.38

(CH), 115.59 (CH_2), 76.48 (C_q), 69.14 (CH_2), 37.00 (CH_2), 25.94 (CH_3), 22.25 (CH_2), 17.98 (CH_3). EI-MS (70 eV): *m/z* (%) = 170 ([*M*]$^+$, < 1), 69 (100), 41 (66), 55 (52), 121 (31), 83 (25), 43 (20), 39 (18), 93 (18), 79 (16), 67 (15). The characterization data for this compound were consistent with the reported data [8].

Synthesis of (*S*)-6-methyl-2-vinylhept-5-ene-1,2-diol ((*S*)-7)

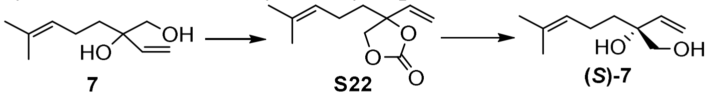

Preparation of 4-(4-methylpent-3-en-1-yl)-4-vinyl-1,3-dioxolan-2-one (S22)

According to a procedure of Khan et al. [9], pyridine (0.743 g, 9.40 mmol, 4.0 equiv) was added to a stirred solution of 6-methyl-2-vinylhept-5-ene-1,2-diol (400 mg, 2.35 mmol, 1.0 equiv) in anhydrous DCM (12mL) at 0 °C under a nitrogen atmosphere. To the mixture was added triphosgene (280 mg, 1.17 mmol, 0.50 equiv) in anhydrous DCM (12 mL) dropwise, and the resulting mixture was stirred for 2 h at room temperature under N_2 atmosphere. Then, saturated NH_4Cl (10 mL) was added to quench the reaction. The resulting mixture was extracted with DCM (3 × 10 mL), and the combined organic phases were dried over $MgSO_4$, filtered, and concentrated under reduced pressure. The residue was purified by silica gel column chromatography (pentane/Et_2O 100:0 to 90:10) to afford 4-(4-methylpent-3-en-1-yl)-4-vinyl-1,3-dioxolan-2-one as a colorless liquid (389 mg, 1.98 mmol, 85%). R_f = 0.61 (pentane/ Et_2O 1:1). ^{1}H NMR (300 MHz, $CDCl_3$): δ (ppm) = 5.86 (dd, *J* = 17.3, 11.0 Hz, 1H), 5.44 (dd, *J* = 17.3, 0.5 Hz, 1H), 5.35 (dd, *J* = 11.0, 0.5 Hz, 1H), 5.12–4.96 (m, 1H), 4.24 (d, *J* = 1.5 Hz, 2H), 2.22–1.95 (m, 2H), 1.87–1.83 (m, 1H), 1.84–1.79 (m, 1H), 1.74–1.65 (d, *J* = 1.1 Hz, 3H), 1.59 (d, *J* = 1.1 Hz, 3H). ^{13}C NMR, DEPT (75.5 MHz, $CDCl_3$): δ (ppm) = 154.57 (C(O)), 136.14 (CH), 133.41 (C_q), 122.29 (CH), 116.67 (CH_2), 84.95 (C_q), 73.46 (CH_2), 38.17 (CH_2), 25.78 (CH_3), 21.98 (CH_2), 17.83 (CH_3). EI-MS (70 eV): *m/z* (%) = 196 ([*M*]$^+$, < 1), 68 (100), 41 (99), 55 (66), 69 (56), 67 (53), 39 (37), 119 (37), 91 (28), 92 (28), 93 (27). The characterization data for this compound were consistent with the reported data [9].

Preparation of (*S*)-6-methyl-2-vinylhept-5-ene-1,2-diol ((*S*)-7)

Following a modified procedure of Khan et al.[9], in an oven dried screw-cap reaction tube equipped with a magnetic stir bar, tris(dibenzylideneacetone)dipalladium(0) chloroform adduct (0.58 mg, 0.56 µmol, 2.5 mol%), (*S*,*S*,*S*)-(+)-(3,5-dioxa-4-phosphacyclohepta[2,1-a:3,4-a']dinaphthalen-4-yl)bis(1-phenylethyl)amine (1.2 mg, 2.2 µmol, 10 mol%) and 4-(4-methylpent-3-en-1-yl)-4-vinyl-1,3-dioxolan-2-one (4.4 mg, 22.4 µmol) were added. The reaction tube was sealed with a rubber septum, then the atmosphere was replaced by nitrogen. BEt_3 (1.0 M solution in THF, 2.2 µmol, 10 mol%), distilled H_2O (4.0 µL, 0.224 mmol, 10 equiv), and anhydrous THF (0.1 mL) were added sequentially via syringe. The resulting mixture was stirred at room temperature for 24 h. The reaction mixture was cooled to room temperature, and the solvent was removed under reduced pressure. The residue was purified by silica gel column chromatography (pentane/Et_2O 3:1 to 2:1) to afford (*S*)-6-methyl-2-vinylhept-5-ene-1,2-diol as a colorless liquid (0.9 mg, 5.3 µmol, 24%, 76% *ee*). ^{1}H NMR (300 MHz, $CDCl_3$): δ (ppm) = 5.80 (dd, *J* = 17.3, 10.8 Hz, 1H), 5.35 (dd, *J* = 17.3, 1.4 Hz, 1H), 5.26 (dd, *J* = 10.8, 1.4 Hz, 1H), 5.11 (dddt, *J* = 8.8, 5.7, 2.8, 1.4 Hz, 2H), 3.48 (d, *J* = 1.3 Hz, 3H), 1.94–2.17 (m, 4H), 1.72–1.66 (overlapped, 1H), 1.67 (s, 3H), 1.60 (s, 3H), 1.51 (ddd, *J* = 13.7, 10.1, 5.7 Hz, 1H). ^{13}C NMR, DEPT (75.5 MHz, $CDCl_3$): δ (ppm) = 140.80 (CH), 132.40 (C_q), 124.27 (CH), 115.42 (CH_2), 76.38 (C_q), 69.00 (CH_2), 36.89 (CH_2), 25.82 (CH_3), 22.13 (CH_2), 17.85 (CH_3). The enantiomer composition was determined by GC on HYDRODEX β-6TBDM phase (Macherey & Nagel). The temperature program started at 50 °C, increased at 5 °C/min to 80 °C, held for 180 min, and then ramped at 20 °C/min to the final temperature of 320 °C. The characterization data for this compound were consistent with the reported data [9].

Synthesis of 6-oxononan-1-ol (8)

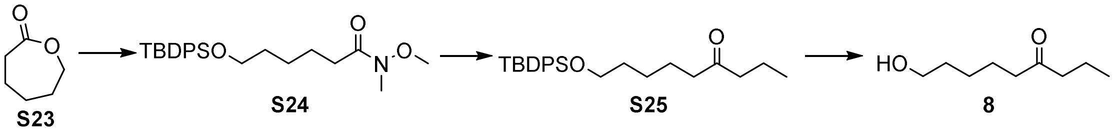

Preparation of 6-((tert-butyldiphenylsilyl)oxy)-*N*-methoxy-*N*-methylhexanamide (S24)

According to a procedure of Kudashev et al. [10], isopropylmagnesium chloride (2.0 M in THF, 135 mL, 270 mmol, 2.2 equiv) was added dropwise to a stirred solution of ε-caprolactone (15.0 g, 123 mmol, 1.0 equiv) and N,O-dimethylhydroxylamine hydrochloride (13.2 g, 135 mmol, 1.1 equiv) in anhydrous THF (200 mL) at 0 °C. The resulting mixture was allowed to warm to room temperature. After 1 h, it was cooled to 0 °C, and saturated NH_4Cl solution (100 mL) was added, followed by water. The resulting mixture was extracted with DCM (3 × 50 mL). The combined organic phases were washed with saturated NaCl solution (50 mL) and dried over $MgSO_4$, filtered, and the solvent was removed under reduced pressure to provide 6-hydroxy-*N*-methoxy-*N*-methylhexanamide, which was used without purification in the next reaction.

The crude product was dissolved in anhydrous DCM (300 mL) and stirred at 0 °C. Imidazole (11.7 g, 172 mmol, 1.3 equiv) and DMAP (1.61 g, 13.2 mmol, 0.1 equiv) were added to the solution, followed by dropwise addition of TBDPSCl (37.1 g, 135 mmol, 1.02 equiv). The reaction was allowed to warm to room temperature over 1 h and stirred for an additional 15 h. Then, water (100 mL) was added to quench the reaction. The phases were separated, and the aqueous phase was extracted with DCM (3 × 50 mL). The combined organic phases were washed with saturated NaCl solution (50 mL) and dried over $MgSO_4$, filtered, and the solvent was removed under reduced pressure. The crude product was purified by column chromatography (SiO_2, pentane/Et_2O 100:0 to 93:7) to afford 6-((*tert*-butyldiphenylsilyl)oxy)-*N*-methoxy-*N*-methylhexanamide as a colorless liquid (34.6 g, 83.6 mmol, 63% over two steps). R_f = 0.66 (pentane/Et_2O 1:1). ^{1}H NMR (300 MHz, $CDCl_3$): δ (ppm) = 7.77–7.62 (m, 4H), 7.49–7.32 (m, 6H), 3.68 (t, *J* = 6.4 Hz, 2H), 3.67 (s, 3H), 3.18 (s, 3H), 2.41 (t, *J* = 7.6 Hz, 2H), 1.74–1.51 (m, 4H), 1.48–1.37 (m, 2H), 1.05 (s, 9H). ^{13}C NMR, DEPT (76 MHz, $CDCl_3$): δ (ppm) = 135.67 (4C, CH_{Ar}), 134.21 (2C, C_{Ar}), 129.61 (2C, CH_{Ar}), 127.70 (4C, CH_{Ar}), 63.91 (CH_2), 61.30 (CH_3), 32.49 (CH_2), 32.02 (CH_2), 26.98 (3C, CH_3), 25.78 (CH_2), 24.57 (CH_2), 19.33 (C_q). FT-IR: ν/cm^{-1} = 2937, 2861, 1666, 1461, 1424, 1381, 1180, 1100, 1000, 818, 739, 698, 611, 496. EI-MS (70 eV): *m/z* (%) =

356 (100), 135 (17), 183 (16), 213 (12), 61 (11), 357 (30), 199 (16), 181 (12), 41 (12), 197 (10). HRESIMS *m/z* calcd. for $C_{24}H_{38}OSiNa$: 436.22784 [*M*]+, found: 436.22813.

Preparation of 9-((*tert*-butyldiphenylsilyl)oxy)nonan-4-one (S25)

Following a modified procedure of Kudashev et al. [10], A solution of *n*-propylmagnesium bromide, freshly prepared from the respective *n*-propyl bromide (357 mg, 2.90 mmol, 2.0 equiv) and magnesium (105 mg, 4.35 mmol, 3.0 equiv) in Et_2O (6.0 mL), was added dropwise to a stirred mixture of 6-((*tert*-butyldiphenylsilyl)oxy)-*N*-methoxy-*N*-methylhexanamide (600 mg, 1.45 mmol, 1.0 equiv) in anhydrous THF (4.2 mL) at 0 °C. The mixture was stirred for 1.5 h, and then saturated NH_4Cl solution (5.0 mL) was added to quench the reaction. The phases were separated, and the aqueous layer was extracted with Et_2O (3 × 5 mL). The combined organic phases were dried over $MgSO_4$, filtered, and the solvent was removed under reduced pressure. The crude product was purified by column chromatography (SiO_2, pentane/Et_2O 100:0 to 97:3) to afford 9-((*tert*-butyldiphenylsilyl)oxy)nonan-4-one as a colorless liquid (523 mg, 1.32 mmol, 91%). R_f = 0.49 (pentane/Et_2O 9:1). ^{1}H NMR (300 MHz, $CDCl_3$): δ (ppm) =7.73–7.63 (m, 4H), 7.50–7.32 (m, 6H), 3.66 (t, *J* = 6.4 Hz, 2H), 2.38 (t, *J* = 7.4 Hz, 2H), 2.35 (t, *J* = 7.4 Hz, 2H), 1.68–1.50 (m, 6H), 1.42–1.28 (m, 2H), 1.05 (s, 9H), 0.91 (t, *J* = 7.4 Hz, 3H). ^{13}C NMR (75.5 MHz, $CDCl_3$): δ (ppm) = 211.53 (C(O)), 135.70 (4C, CH_{Ar}), 134.21 (2C C_q), 129.65 (2C, CH_{Ar}), 127.72 (4C, CH_{Ar}), 63.85 (CH_2), 44.86 (CH_2), 42.93 (CH_2), 32.48 (CH_2), 27.00 (3C, CH_3), 25.64 (CH_2), 23.76 (CH_2), 19.35 (C_q), 17.44 (CH_2), 13.92 (CH_3). FT-IR: ν/cm^{-1} = 2937, 2863, 1713, 1464, 1424, 1374, 1188, 1099, 1005, 818, 738, 698, 611, 496. EI-MS (70 eV): *m/z* (%) = 199 (100), 139 (28), 200 (19), 71 (18), 81 (14), 339 (49), 43 (24), 181 (18), 41 (15), 340 (14). HRESIMS *m/z* calcd. for $C_{25}H_{36}O_2SiNa$: 419.23768 [*M*+Na]+, found: 419.23796.

Preparation of 6-oxononan-1-ol (8)

Tetrabutylammonium fluoride (1.0 M in THF, 1.7 mL, 1.70 mmol, 1.5 equiv) was added dropwise to 9-((*tert*-butyldiphenylsilyl)oxy)nonan-4-one (449 mg, 1.13 mmol, 1.0 equiv) at 0 °C. The mixture was allowed to warm to room temperature and stirred for 2 h. Then, water (1.0 mL) was added to quench the reaction. The resulting mixture was extracted with Et_2O (3 × 2 mL), and the combined organic phases were dried over $MgSO_4$, filtered, and concentrated under reduced pressure. The residue was purified by silica gel column chromatography (pentane/Et_2O 9:1 to 1:2) to afford 6-oxo-nonan-1-ol as a colorless liquid (164 mg, 1.04 mmol, 92%). R_f = 0.22 (pentane/Et_2O 2:1). ^{1}H NMR (300 MHz, $CDCl_3$): δ (ppm) = 3.62 (t, *J* = 6.5 Hz, 2H), 2.39 (t, *J* = 7.3 Hz, 2H), 2.35 (d, *J* = 7.3 Hz, 2H), 1.70 (br s, 1H), 1.67–1.48 (m, 6H), 1.41–1.22 (m, 2H), 0.89 (t, *J* = 7.4 Hz, 3H). ^{13}C NMR (75.5 MHz, $CDCl_3$): δ (ppm) = 211.61 (C(O)), 62.69 (CH_2), 44.88 (CH_2), 42.75 (CH_2), 32.53 (CH_2), 25.46 (CH_2), 23.52 (CH_2), 17.41 (CH_2), 13.86 (CH_3). EI-MS (70 eV): *m/z* (%) = 158 ([*M*]+, 2), 43 (100), 71 (93), 58 (56), 86 (40), 39 (20), 69 (95), 41 (72), 55 (41), 97 (39), 73 (17). The characterization data for this compound were consistent with the reported data [1].

Synthesis of 7-methyl-3-methyleneoct-6-ene-1,2-diol (9)

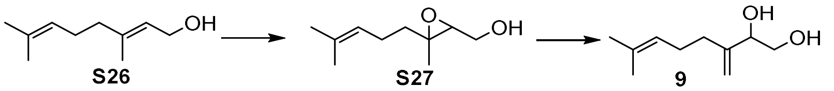

S26 S27 9

Preparation of (3-methyl-3-(4-methylpent-3-en-1-yl)oxiran-2-yl)methanol (S27)

According to a procedure of Davis et al. [11], ^{t}BuO$_2$H (5–6 M in decane, 2.1 mL, 6.48 mmol, 1.8 equiv) was added dropwise to a stirred solution of geraniol (1.00 g, 6.48 mmol, 1.0 equiv) and vanadyl acetylacetonate (17.2 mg, 0.0648 mmol, 1.0 mol%) in benzene (20 mL) at 0 °C over 15 min. The resulting mixture was allowed to warm to room temperature and stirred for 15 h. This mixture was washed with saturated Na_2SO_3 solution (10 mL), and the organic layer was dried over $MgSO_4$, filtered, and concentrated under reduced pressure. The residue was purified by silica gel column chromatography (pentane/Et_2O 100:0 to 60:40) to afford (3-methyl-3-(4-methylpent-3-en-1-yl)oxiran-2-yl)methanol as a colorless liquid (807 mg, 4.74 mmol, 74%). R_f = 0.22 (pentane/ Et_2O 2:1). ^{1}H NMR (300 MHz, $CDCl_3$): δ (ppm) = 5.08 (tdt, *J* = 7.1, 2.9, 1.4 Hz, 1H), 3.82 (ddd, *J* = 11.6, 6.9, 4.3 Hz, 1H), 3.68 (ddd, *J* = 11.6, 6.6, 4.4 Hz, 1H), 2.97 (dd, *J* = 6.6, 4.3 Hz, 1H), 2.07 (q, *J* = 7.7 Hz, 2H), 1.76 (br s, 1H), 1.73–1.64 (m, 1H), 1.61 (s, 3H), 1.55–1.40 (m, 1H), 1.30 (s, 3H). ^{13}C NMR, DEPT (75.5 MHz, $CDCl_3$): δ (ppm) = 132.31 (C_q), 123.47 (CH), 63.01 (CH), 61.62 (CH_2), 61.30 (C_q), 38.63 (CH_2), 25.82 (CH_3), 23.83 (CH_2), 17.80 (CH_3), 16.90 (CH_3). EI-MS (70 eV): *m/z* (%) = 170 ([*M*]+, < 1), 41 (100), 82 (80), 69 (79), 43 (60), 67 (47), 109 (36), 55 (36), 39 (24), 81 (20), 83 (19). The characterization data for this compound were consistent with the reported data [11].

Preparation of 7-methyl-3-methyleneoct-6-ene-1,2-diol (9)

LDA was prepared from ^{i}Pr$_2$NH (10.5 g, 104 mmol, 2.2 equiv) and ^{n}BuLi (1.6 M in hexane, 59 mL, 113 mmol, 2.4 equiv) in anhydrous THF (16 mL) at –78 °C under a nitrogen atmosphere. (3-Mhethyl-3-(4-methylpent-3-en-1-yl)oxiran-2-yl)methanol (8.00 g, 47.0 mmol, 1.0 equiv) in anhydrous THF (80 mL) was added to the stirred solution of the freshly prepared LDA at –78 °C, and the resulting mixture was stirred for an additional 20 min at the same temperature. The reaction mixture was warmed to –10 °C and stirred for 4 h. The reaction was quenched at 0 °C with saturated NH_4Cl solution (40 mL). The resulting mixture was extracted with DCM (3 × 20 mL), and the combined organic phases were dried over $MgSO_4$, filtered, and concentrated under reduced pressure. The residue was purified by silica gel column chromatography (pentane/Et_2O 100:0 to 70:30) to afford 7-methyl-3-methyleneoct-6-ene-1,2-diol as a colorless liquid (4.29 g, 25.2 mmol, 54%). R_f = 0.38 (pentane/Et_2O 1:3). ^{1}H NMR (300 MHz, $CDCl_3$): δ (ppm) = 5.29–5.10 (m, 2H), 5.14–5.05 (tdt, *J* = 7.1, 2.9, 1.4 Hz, 1H), 5.03–4.90 (m, 1H), 4.19 (dddd, *J* = 7.3, 3.4, 1.2, 0.7 Hz, 1H), 3.69 (dd, *J* = 11.3, 3.4 Hz, 1H), 3.53 (dd, *J* = 11.3, 7.3 Hz, 1H), 2.23 (br s, 2H), 2.21–2.11 (m, 2H), 2.11–1.92 (m, 2H), 1.71–1.65 (m, 3H), 1.65–1.57 (m, 3H). ^{13}C NMR, DEPT (75.5 MHz, $CDCl_3$): δ (ppm) = 148.39 (C_q), 132.34 (C_q), 123.75 (CH_2), 111.02 (CH), 75.21 (CH_2), 65.73 (CH), 32.69 (CH_3), 26.72 (CH_3), 25.81 (CH_2), 15.39 (CH_2). EI-MS (70 eV): *m/z* (%) = 170 ([*M*]+, 1), 43 (100), 41 (74), 39 (52), 81 (49), 55 (46), 59 (42), 67 (35), 69 (34), 97 (30), 53 (30). The characterization data for this compound were consistent with the reported data [12].

Synthesis of (*S*)-7-methyl-3-methyleneoct-6-ene-1,2-diol ((*S*)-9)

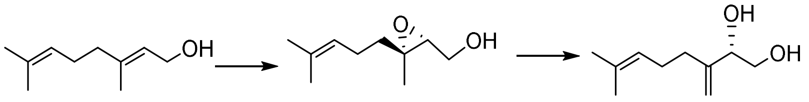

S26 **(2*R*,3*R*)-S27** **(*S*)-9**

Preparation of ((2*R*,3*R*)-3-methyl-3-(4-methylpent-3-en-1-yl)oxiran-2-yl)methanol ((2*R*, 3*R*)-S27)

Following a modified procedure of Chen et al. [13], Ti(O^{i}Pr)$_4$ (921 mg, 3.24 mmol, 1.0 equiv) and ^{t}BuO$_2$H (5–6 M in dodecane, 1.2 mL, 6.48 mmol, 2.0 equiv) were added to a stirred solution of D-(-)-tartrate (802 mg, 3.89 mmol, 1.2 equiv) in anhydrous DCM (5.0 mL) at –10 °C, and the resulting mixture was stirred for an additional 20 min. The reaction mixture was cooled to –25 °C, and then geraniol (500 mg, 3.24 mmol, 1.0 equiv) was added. After 1 h at –25 °C, the mixture was allowed to warm to 0 °C, and water (2.0 mL) was added. The mixture was warmed again to room temperature and stirred for 30 min. 3.0 M NaOH solution (2.0 mL) was added to the reaction, and the resulting mixture was stirred for an additional 30 min. 3.0 M citric acid solution (2.0 mL) was added to the stirred mixture, and the resulting solution was stirred again for 30 min. The layers were separated, and the aqueous phase was extracted with DCM (3 × 2 mL). The combined organic phases were dried over $MgSO_4$, filtered, and concentrated under reduced pressure. The residue was purified by silica gel column chromatography (pentane/Et_2O 100:0 to 75:25) to afford ((2R,3*R*)-3-methyl-3-(4-methylpent-3-en-1-yl)oxiran-2-yl)methanol as a colorless liquid (408 mg, 2.40 mmol, 74%). ^{1}H NMR (300 MHz, $CDCl_3$): δ (ppm) = 5.06 (dddd, *J* = 7.1, 5.7, 2.8, 1.4 Hz, 1H), 3.80 (ddd, *J* = 12.2, 4.2, 1.5 Hz, 1H), 3.64 (ddd, *J* = 12.2, 6.8, 1.6 Hz, 1H), 3.09–2.84 (m, 1H), 2.46 (br s, 1H), 2.06 (q, *J* = 7.8 Hz, 2H), 1.71–1.64 (m, 1H), 1.66 (s, 3H), 1.58 (s, 3H), 1.54–1.36 (m, 1H), 1.27 (s, 3H). ^{13}C NMR, DEPT (75.5 MHz, $CDCl_3$): δ (ppm) = 132.32 (C_q), 123.43 (CH), 63.20 (CH), 61.51 (CH_2), 61.31 (C_q), 38.60 (CH_2), 25.76 (CH_3), 23.77 (CH_2), 17.74 (CH_3), 16.84 (CH_3). The characterization data for this compound was consistent with the reported data [13].

Preparation of (*S*)-7-methyl-3-methyleneoct-6-ene-1,2-diol ((*S*)-9)

Epoxide opening was performed as described for the preparation of 7-methyl-3-methyleneoct-6-ene-1,2-diol. A solution of ((2*R*,3R)-3-methyl-3-(4-methylpent-3-en-1-yl)oxiran-2-yl)methanol (408 mg, 2.40 mmol, 1.0 equiv) in anhydrous THF (5 mL) was treated with a freshly prepared solution of LDA (5.3 mmol, 2.2 equiv) in THF (1.5 mL) to afford (*S*)-7-methyl-3-methyleneoct-6-ene-1,2-diol as a colorless liquid (184 mg, 1.08 mmol, 45%, 33% *ee*). ^{1}H NMR (300 MHz, $CDCl_3$): δ (ppm) = 5.17–5.13 (m, 1H), 5.14–5.05 (m, 1H), 5.03–4.90 (m, 1H), 4.19 (dddd, *J* = 7.2, 3.4, 1.2, 0.7 Hz, 1H), 3.69 (dd, *J* = 11.3, 3.4 Hz, 1H), 3.53 (dd, *J* = 11.3, 7.3 Hz, 1H), 2.23 (br s, 1H), 2.21–2.11 (m, 2H), 2.11–1.92 (m, 2H), 1.71–1.65 (m, 3H), 1.65–1.57 (m, 3H). ^{13}C NMR, DEPT (75.5 MHz, $CDCl_3$): δ (ppm) = 148.39 (C_q), 132.34 (C_q), 123.75 (CH_2), 111.02 (CH), 75.21 (CH_2), 65.73 (CH), 32.69 (CH_3), 26.72 (CH_3), 25.81 (CH_2), 15.39 (CH_2). The enantiomer composition was determined by GC on a Beta-Dex^{TM} 225 chiral capillary column (SUPELCO). The temperature program started at 100 °C, was held for 120 min, and then ramped at 20 °C/min to the final temperature of 250 °C.

Synthesis of (*S*)-1′-hydroxymethyleugenol ((*S*)-1′-HME, (*S*)-12)

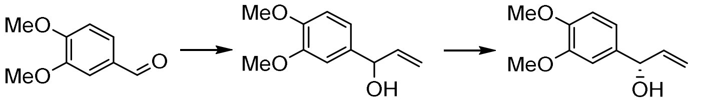

S28 **12** **(*S*)-12**

Preparation of 1′-hydroxymethyleugenol (1′-HME, 12)

Following a modified procedure of Stambaský et al. [14], vinylmagnesium bromide (1.0 M in THF, 3.6 mL, 3.61 mmol, 1.2 equiv) was added dropwise to a stirred solution of 3,4-dimethoxybenzaldehyde (500 mg, 3.01 mmol, 1.0 equiv) in Et_2O (1.5 mL). After 2 h at the same temperature, saturated NH_4Cl solution (1.0 mL) was added to quench the reaction. The phases were separated, and the aqueous layer was extracted with Et_2O (3 × 2 mL). The combined organic phases were dried over $MgSO_4$ and concentrated under reduced pressure. The residue was dissolved in MeOH (300 mL), and K_2CO_3 (33.2 g, 241 mmol, 2.0 equiv) was added to the solution at 0 °C. The resulting mixture was allowed to warm to room temperature and stirred for 30 min. The solvent was removed under reduced pressure. The residue was dissolved in EtOAc (100 mL), and the solution was washed with water (30 mL) and brine (30 mL). The residue was purified by silica gel column chromatography (pentane/Et_2O 90:10 to 80:20) to afford 1-(3,4-dimethoxyphenyl)prop-2-en-1-ol as a colorless liquid (579 mg, 2.98 mmol, 99%). R_f = 0.43 (pentane/Et_2O 1:3). ^{1}H NMR (300 MHz, $CDCl_3$): δ (ppm) = 6.95–6.87 (m, 2H), 6.86–6.78 (d, *J* = 8.1, 1H), 6.04 (ddd, *J* = 17.1, 10.3, 5.9 Hz, 1H), 5.34 (dt, *J* = 17.1, 1.5 Hz, 1H), 5.19 (dt, *J* = 10.3, 1.5 Hz, 1H), 5.14 (d, *J* = 5.9 Hz, 1H), 3.88 (s, 3H), 3.86 (s, 3H), 2.03 (br s, 1H). ^{13}C NMR, DEPT (75.5 MHz, $CDCl_3$): δ (ppm) = 149.21 (C_{Ar}), 148.71 (C_{Ar}), 140.36 (CH), 135.40 (C_{Ar}), 118.76 (CH), 115.02 ($\underline{C}H_2$=CH), 111.13 (CH), 109.59 (CH), 75.19 (CH), 56.05 (CH_3), 55.96 (CH_3). EI-MS (70 eV): *m/z* (%) = 194 ([*M*]$^+$, 100), 139 (93), 55 (54), 163 (54), 151 (36), 165 (26), 77 (23), 138 (23), 91 (20), 167 (18). The characterization data for this compound were consistent with the reported data [14].

Preparation of (*S*)-1′-hydroxymethyleugenol ((*S*)-1-HME, (*S*)-12)

According to a procedure of Stambaský et al. [14], Novozyme 435 (10.1 mg, 13.5 wt%) was added to a stirred solution of 1-(3,4-dimethoxyphenyl)prop-2-en-1-ol (75.0 mg, 0.386 mmol, 1.0 equiv), isopropenyl acetate (160 mg, 1.60 mmol, 4.2 equiv) and 4Å molecular sieves powder (50.2 mg, 67 wt%) in dry toluene (4.0 mL). The resulting mixture was stirred for 18 h at 40 °C. The precipitate was removed by filtration and the filtrate solution was concentrated under reduced pressure. The residue was purified by silica gel column chromatography (pentane/Et_2O 90:10 to 70:30) to afford (*S*)-1-(3,4-dimethoxyphenyl)prop-2-en-1-ol as a colorless liquid (30.3 mg, 0.156 mmol, 81%). ^{1}H NMR (300 MHz, $CDCl_3$): δ (ppm) = 6.94 – 6.91 (m, 1H), 6.91 (ddd, *J* = 8.0, 2.0, 0.6 Hz, 1H), 6.83 (d, *J* = 8.0 Hz, 1H), 6.04 (ddd, *J* = 17.1, 10.3, 5.8 Hz, 1H), 5.34 (dt, *J* = 17.1, 1.4 Hz, 1H), 5.19 (dt, *J* = 10.3, 1.4 Hz, 1H), 5.15 (dd, *J* = 5.8, 0.6 Hz, 1H), 3.88 (s, 3H), 3.87 (s, 3H). ^{13}C NMR, DEPT (75.5 MHz, $CDCl_3$): δ (ppm) = 149.21 (C_{Ar}), 148.72 (C_{Ar}), 140.36 (CH), 135.40 (C_{Ar}), 118.76 (CH), 115.03 ($\underline{C}H_2$=CH),

111.14 (CH), 109.59 (CH), 75.20 (CH), 56.05 (CH_3), 55.96 (CH_3) The enantiomer composition was determined by GC on a HYDRODEX β-6TBDM phase (Macherey & Nagel). The temperature program started at 120 °C, held for 120 min, and then ramped at 20 °C/min to the final temperature of 230 °C.The characterization data for this compound were consistent with the reported data [14].

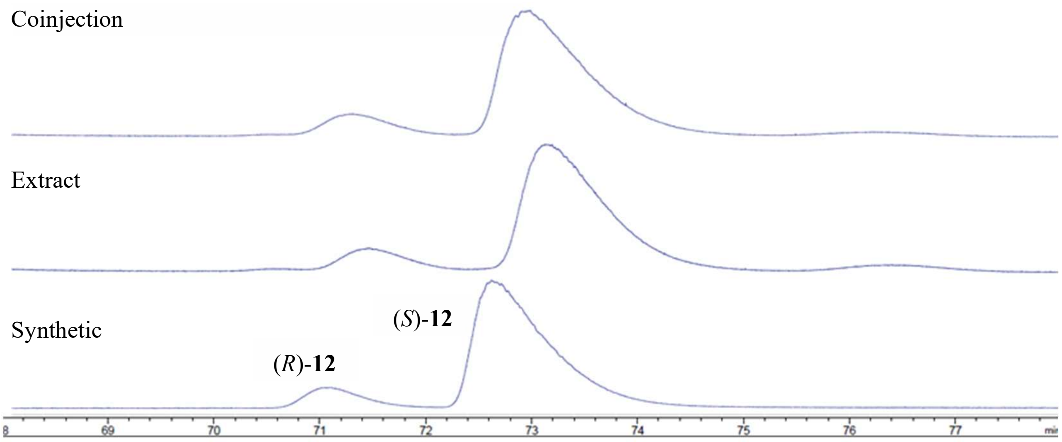

Fig. S26 Chiral GC comparison between synthetic (*S*)-**12** and rectal gland extracts from a ME-fed male *B. umbrosa* using HYDRODEX β-6TBDM phase (Macherey & Nagel).

Synthesis of 2-allyl-4,5-dimethoxyphenol (DMP, 13)

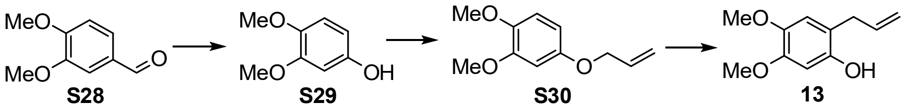

Preparation of 3,4-dimethoxyphenol (S29)

According to a procedure of Häußler and Gütschow [15], meta-chloroperoxybenzoic acid (22.8 g, 132 mmol, 1.1 equiv) was added dropwise to a stirred solution of 3,4-dimethoxybenzaldehyde (20.0 g, 121 mmol, 1.0 equiv) in DCM (200 mL). The resulting mixture was stirred at room temperature for 15 h. The reaction mixture was washed with saturated Na_2SO_3 solution (50 mL) and brine (50 mL). The organic layer was dried over $MgSO_4$, filtered, and concentrated under reduced pressure. The residue was dissolved in MeOH (300 mL), and K_2CO_3 (33.2 g, 241 mmol, 2.0 equiv) was added to the solution at 0 °C. The resulting mixture was allowed to warm to room temperature and stirred for 30 min. The solvent was removed under reduced pressure. The residue was dissolved in EtOAc (100 mL), and the solution was washed with water (30 mL) and brine (30 mL). The organic layer was dried over $MgSO_4$, filtered, and concentrated under reduced pressure to afford 3,4-dimethoxyphenol as a colorless liquid (18.2 g, 118 mmol, 98%). R_f = 0.11 (DCM/MeOH 95:5). ^{1}H NMR (300 MHz, $CDCl_3$): δ (ppm) = 6.73 (d, J = 8.6 Hz, 1H), 6.47 (d, J = 2.8 Hz, 1H), 6.34 (dd, J = 8.6, 2.8 Hz, 1H), 3.85 (s, 3H), 3.82 (s, 3H). ^{13}C NMR, DEPT (75.5 MHz, $CDCl_3$): δ (ppm) = 150.14 (C_{Ar}), 150.09 (C_{Ar}), 143.45 (C_{Ar}), 112.43 (CH_{Ar}), 105.87 (CH_{Ar}), 100.71 (CH_{Ar}), 56.69 (CH_3), 55.99 (CH_3). EI-MS (70 eV): m/z (%) = 154 ([M]$^+$, 91), 111 (100), 139 (87), 93 (37), 69 (31), 65 (30), 55 (30), 53 (24), 68 (21), 39 (20). The characterization data for this compound were consistent with the reported data [15].

Preparation of 4-(allyloxy)-1,2-dimethoxybenzene (S30)

According to a procedure of Benbow and Katoch-Rouse [16], 3,4-dimethoxyphenol (18.0 g, 117 mmol, 1.0 equiv) and allyl bromide (17.0 g, 140 mmol, 1.2 equiv) were added to a stirred solution of K_2CO_3 (48.2 g, 350 mmol, 3.0 equiv) in acetone (117 mL) at 0 °C. The resulting mixture was heated to reflux for 16 h, and then approximately 90% of the acetone was removed under reduced pressure. EtOAc (50 mL) was added to the remaining solution, and the resulting mixture was washed with water (30 mL) and brine (30 mL). The organic layer was dried over $MgSO_4$, filtered, and concentrated under reduced pressure. The residue was purified by silica gel column chromatography (pentane/Et_2O 100:0 to 90:10) to afford 4-(allyloxy)-1,2-dimethoxybenzene as a white solid (22.1 g, 114 mmol, 98%). R_f = 0.70 (pentane/Et_2O 1:3). ^{1}H NMR (300 MHz, $CDCl_3$): δ (ppm) = 6.77 (d, J = 8.7 Hz, 1H), 6.55 (d, J = 2.8 Hz, 1H), 6.41 (dd, J = 8.7, 2.8 Hz, 1H), 6.06 (ddt, J = 17.3, 10.7, 5.4 Hz, 1H), 5.41 (dq, J = 17.3, 1.6 Hz, 1H), 5.28 (dq, J = 10.7, 1.6 Hz, 1H), 4.49 (dt, J = 5.4, 1.6 Hz, 2H), 3.85 (s, 3H), 3.83 (s, 3H). ^{13}C NMR, DEPT (75.5 MHz, $CDCl_3$): δ (ppm) = 153.33 (C_{Ar}), 149.96 (C_{Ar}), 143.71 (C_{Ar}), 133.62 ($\underline{C}H{=}CH_2$), 117.73 ($\underline{C}H_2{=}CH$), 111.85 (CH_{Ar}), 104.15 (CH_{Ar}), 101.24 (CH_{Ar}), 69.53 (CH_2), 56.55 (CH_3), 55.95 (CH_3). EI-MS (70 eV): m/z (%) = 57 (100), 153 (100), 125 (41), 194 (40), 110 (25), 95 (11), 79 (10), 154 (10), 41 (10), 39 (9). The characterization data for this compound were consistent with the reported data [16].

Preparation of 2-allyl-4,5-dimethoxyphenol (13)

According to a procedure of Benbow and Katoch-Rouse [16], a stirred solution of 4-(allyloxy)-1,2-dimethoxybenzene (22.1 g, 114 mmol, 1.0 equiv) in *N*,*N*-diethylaniline (250 mL) was heated to reflux for 4 h under a nitrogen atmosphere. The reaction mixture was cooled to 0 °C and diluted with Et_2O (250 mL). The resulting mixture was washed with 10% HCl solution, and the organic layer was dried over $MgSO_4$, filtered, and concentrated under reduced pressure. The residue was purified by silica gel column chromatography (pentane/Et_2O 100:0 to 90:10) to afford 2-allyl-4,5-dimethoxyphenol as a white solid (17.3 g, 89.1 mmol, 78%). R_f = 0.53 (pentane/Et_2O 1:3). ^{1}H NMR (300 MHz, $CDCl_3$): δ (ppm) = 6.62 (s, 1H), 6.46 (s, 1H), 6.00 (ddt, J = 17.5, 9.7, 6.4 Hz, 2H), 5.19 (m, 1H), 5.14 (m, 1H), 3.82 (s, 3H), 3.82 (s, 3H), 3.34 (dt, J = 6.4, 1.5 Hz, 2H). ^{13}C NMR, DEPT (75.5 MHz, $CDCl_3$): δ (ppm) = 148.69 (C_{Ar}), 148.23 (C_{Ar}), 143.23 (C_{Ar}), 136.68 ($\underline{C}H{=}CH_2$), 116.52 ($\underline{C}H_2{=}CH$), 115.75 (C_{Ar}), 114.02 (CH_{Ar}), 101.41 (CH_{Ar}), 56.73 (CH_3), 56.10 (CH_3), 35.06 (CH_2). EI-MS (70 eV): m/z (%) = 194 ([M]$^+$, 100), 179 (85), 123 (29), 69 (22), 91 (20), 151 (14), 77 (14), 195 (12), 79 (11), 163 (9). The characterization data for this compound were consistent with the reported data [16].

Synthesis of (*Z*)-3,4-dimethoxycinnamyl alcohol (Z-DMC, 14)

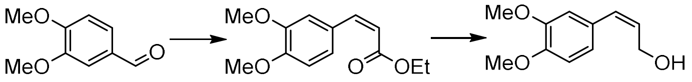

S28 S31 14

Preparation of ethyl (*Z*)-3-(3,4-dimethoxyphenyl)acrylate (S31)

Following a modified procedure of Janicki et al. [17], potassium bis(trimethylsilyl)amide (1.0 M in THF, 15 mL, 15.2 mmol, 1.1 equiv) was added dropwise to a stirred solution of ethyl 2-(bis(2,2,2-trifluoroethyl)phosphoryl)acetate (4.82 g, 14.5 mmol, 1.05 equiv) in anhydrous THF (140 mL) at –78 °C over 20 min. The resulting mixture was stirred for 30 min, and a solution of 3,4-dimethoxybenzaldehyde (2.30 g, 13.8 mmol, 1.0 equiv) in anhydrous THF (70 mL) was added to the mixture at –78 °C. The resulting mixture was stirred for 2 h and then allowed to warm to room temperature and stirred for an additional 1 h. The reaction was quenched at 0 °C with saturated NH_4Cl solution (50 mL). The resulting mixture was extracted with Et_2O (3 × 30 mL), and the combined organic phases were dried over $MgSO_4$, filtered, and concentrated under reduced pressure. The residue was purified by silica gel column chromatography (pentane/Et_2O 100:0 to 90:10) to afford ethyl (*Z*)-3-(3,4-dimethoxyphenyl)acrylate as a white solid (2.00 g, 8.47 mmol, 61%). R_f = 0.78 (pentane/Et_2O 1:3). ^{1}H NMR (300 MHz, $CDCl_3$): δ (ppm) = 7.67 (d, J = 2.1 Hz, 1H), 7.18 (dd, J = 8.3, 2.1 Hz, 1H), 6.83 (d, J = 8.3 Hz, 1H), 6.80 (d, J = 12.8 Hz, 1H), 5.82 (d, J = 12.8 Hz, 1H), 4.18 (q, J = 7.1 Hz, 2H), 3.90 (s, 3H), 3.89 (s, 3H), 1.27 (t, J = 7.1 Hz, 3H). ^{13}C NMR, DEPT (75.5 MHz, $CDCl_3$): δ (ppm) = 166.53 (C(O)), 150.21 (C_{Ar}), 148.39 (C_{Ar}), 143.41 (CH_{Ar} or <u>C</u>H=CH), 127.80 (C_{Ar}), 124.79 (CH_{Ar} or <u>C</u>H=CH), 117.39 (CH_{Ar} or <u>C</u>H=CH), 113.39 (CH_{Ar} or <u>C</u>H=CH), 110.43 (CH_{Ar} or <u>C</u>H=CH), 60.26 (CH_2), 55.99 (CH_3), 55.95 (CH_3), 14.33 (CH_3). FT-IR: □/cm^{-1} = 2947, 2839, 1709, 1608, 1589, 1511, 1455, 1374, 1247, 1170, 1136, 1022, 862, 822, 750, 544, 431. EI-MS (70 eV): *m/z* (%) = 236 ([*M*]$^+$, 100), 191 (52), 164 (20), 237 (14), 77 (11), 147 (8), 119 (8), 133 (8), 163 (7), 91 (7). HREIMS *m/z* calcd. for $C_{13}H_{16}O_4$: 236.10431 [M]$^+$, found: 236.10486.

Preparation of (*Z*)-3,4-dimethoxycinnamyl alcohol (Z-DMC, 14)

Following a modified procedure of Gornati et al. [18], diisobutylaluminium hydride (1.0 M in hexane, 19 mL, 8.42 mmol, 2.2 equiv) was added dropwise to a stirred solution of ethyl (*Z*)-3-(3,4-dimethoxyphenyl)acrylate (2.00 g, 8.42 mmol, 1.0 equiv) in anhydrous DCM (35 mL) at –78 °C over 20 min. EtOH (10 mL) was added to the mixture, and the reaction mixture was allowed to warm to room temperature, followed by the addition of water (13 mL). The resulting mixture was stirred for 30 min at the same temperature. The layers were separated, and the aqueous phase was extracted with DCM (3 × 5.0 mL). The combined organic phases were dried over $MgSO_4$, filtered, and concentrated under reduced pressure. The residue was purified by silica gel column chromatography (pentane/Et_2O 100:0 to 4:1) to afford (*Z*)-3-(3,4-dimethoxyphenyl)prop-2-en-1-ol as a white solid (1.65 g, 8.41 mmol, 100%). R_f = 0.32 (pentane/Et_2O 1:3). ^{1}H NMR (300 MHz, $CDCl_3$): δ (ppm) = 6.88–6.78 (m, 1H), 6.79–6.74 (m, 2H), 6.49 (dt, J = 11.6, 1.6 Hz, 1H), 5.78 (dt, J = 11.6, 6.5 Hz, 1H), 4.42 (dd, J = 6.5, 1.6 Hz, 2H), 3.87 (s, 3H), 3.86 (s, 3H). ^{13}C NMR, DEPT (75.5 MHz, $CDCl_3$): δ (ppm) = 148.74 (C_{Ar}), 148.50 (C_{Ar}), 131.03 (CH_{Ar} or <u>C</u>H=CH), 129.80 (CH_{Ar} or <u>C</u>H=CH) 129.63 (C_{Ar}), 121.60(CH_{Ar} or <u>C</u>H=CH), 112.24 (CH_{Ar} or <u>C</u>H=CH), 111.07 (CH_{Ar} or <u>C</u>H=CH), 59.81 (CH_2), 56.00 (CH_3), 55.96 (CH_3). EI-MS (70 eV): *m/z* (%) = 194 ([*M*]$^+$, 67), 151 (100), 138 (51), 91 (29), 77 (22), 119 (14), 165 (13), 55 (12), 79 (12), 152 (11). The characterization data for this compound were consistent with the reported data [19].

Synthesis of (*E*)-coniferyl alcohol (E-CF, 15)

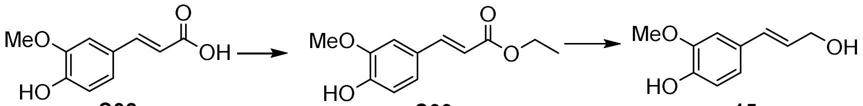

S32 S33 15

Preparation of ethyl (*E*)-3-(4-hydroxy-3-methoxyphenyl)acrylate (S33)

Following a modified procedure of Wang et al. [20], sulfuric acid (2.02 g, 20.6 mmol, 10 mol%) was added dropwise to a stirred solution of (*E*)-3-(4-hydroxy-3-methoxyphenyl)acrylic acid (22.6 g, 117 mmol, 1.0 equiv) in EtOH (150 mL). The resulting mixture was heated to reflux for 12 h, and EtOH was removed under reduced pressure. The water (20 mL) was added to the residue, and the aqueous phase was extracted with DCM (3 × 15 mL). The combined organic phases were washed with saturated $NaHCO_3$ solution (15 mL) and brine (15 mL), dried over $MgSO_4$, filtered, and concentrated under reduced pressure to afford (*E*)-3-(4-hydroxy-3-methoxyphenyl)acrylate as a white solid (25.7 g, 116 mmol, 100%). R_f = 0.52 (pentane/Et_2O 1:3). ^{1}H NMR (300 MHz, $CDCl_3$): δ (ppm) = 7.60 (d, J = 15.9 Hz, 1H), 7.10 – 6.98 (m, 2H), 6.90 (d, J = 8.1 Hz, 1H), 6.28 (d, J = 15.9 Hz, 1H), 6.07 (s, 1H), 4.25 (q, J = 7.1 Hz, 2H), 3.89 (s, 3H), 1.32 (t, J = 7.1 Hz, 2H). ^{13}C NMR, DEPT (75.5 MHz, $CDCl_3$): δ (ppm) = 167.54 (C(O)), 148.15 (C_{Ar}), 147.00 (C_{Ar}), 144.92 (CH_{Ar} or <u>C</u>H=CH), 127.16 (C_{Ar}), 123.19 (CH_{Ar} or <u>C</u>H=CH), 115.74 (CH_{Ar} or <u>C</u>H=CH), 114.96 (CH_{Ar} or <u>C</u>H=CH), 109.55 (CH_{Ar} or <u>C</u>H=CH), 60.58 (CH_2), 56.09 (CH_3), 14.54 (CH_3). EI-MS (70 eV): *m/z* (%) = 222 ([*M*]$^+$, 100), 177 (78), 150 (64), 145 (60), 89 (29), 117 (27), 77 (20), 194 (19), 78 (18), 51 (17) The characterization data for this compound were consistent with the reported data [21].

Preparation of ethyl (*E*)-4-(3-hydroxyprop-1-en-1-yl)-2-methoxyphenol (15)

Following a modified procedure of Wang et al. [20], diisobutylaluminium hydride (1.0 M in hexane, 8.5 mL, 8.46 mmol, 4.0 equiv) was added dropwise to a stirred solution of ethyl (*E*)-3-(4-hydroxy-3-methoxyphenyl)acrylate (500 mg, 2.12 mmol, 1.0 equiv) in anhydrous toluene (2.1 mL) at 0 °C. The resulting mixture was stirred for 1 h, allowed to warm to room temperature and then stirred for an additional 1 h. This mixture was cooled again to 0 °C, and 1 M HCl solution was added to the reaction mixture to adjust the pH to 3.0. The phases were separated, and the aqueous layer was extracted with EtOAc (3 × 5.0 mL), and the combined organic phases were dried over $MgSO_4$, filtered, and concentrated under reduced pressure. The residue was purified by silica gel column chromatography (pentane/Et_2O 1:1) to afford (*E*)-4-(3-hydroxyprop-1-en-1-yl)-2-methoxyphenol as a white solid (336 mg, 1.86 mmol, 88%). R_f = 0.32 (pentane/Et_2O 2:1). ^{1}H NMR (300 MHz, $CDCl_3$): δ (ppm) = 6.92–6.85 (m, 3H), 6.51 (d, J = 15.9 Hz, 1H), 6.28–6.13 (m, 1H), 5.86 (s br, 1H), 4.29 (dd, J = 6.0, 1.4 Hz, 2H), 3.87 (s, 3H), 1.64 (br, 1H). ^{13}C NMR, DEPT (75.5 MHz, $CDCl_3$): δ (ppm) = 146.77 (C_q), 145.67 (C_q), 131.43 (CH), 129.33 (C_q), 126.21 (CH), 120.37 (CH), 114.61 (CH), 108.50 (CH), 63.89 (CH_2), 55.96 (CH_3). EI-MS (70 eV): *m/z* (%) = 180 (76, [*M*]$^+$), 137 (100), 124 (51), 91 (34), 119 (26), 77 (17), 109 (17), 138 (16), 103 (15), 147 (13). The characterization data for this compound were consistent with the reported data [20].

5 NMR Spectra

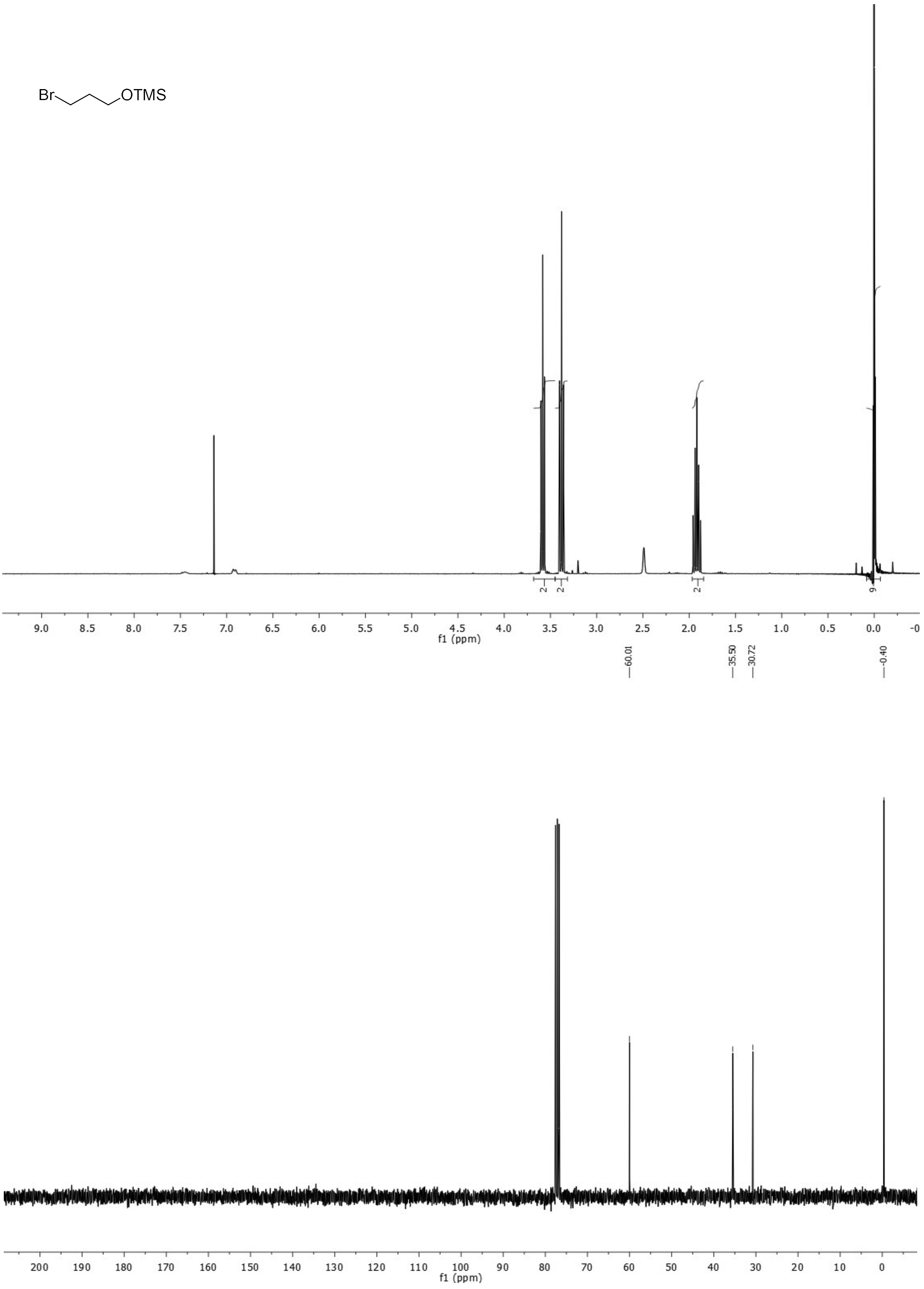

Fig. S27 ^1H NMR (300 MHz, $CDCl_3$) and ^{13}C NMR (75.5 MHz, $CDCl_3$) spectra of (3-bromopropoxy)trimethylsilane (**S6**).

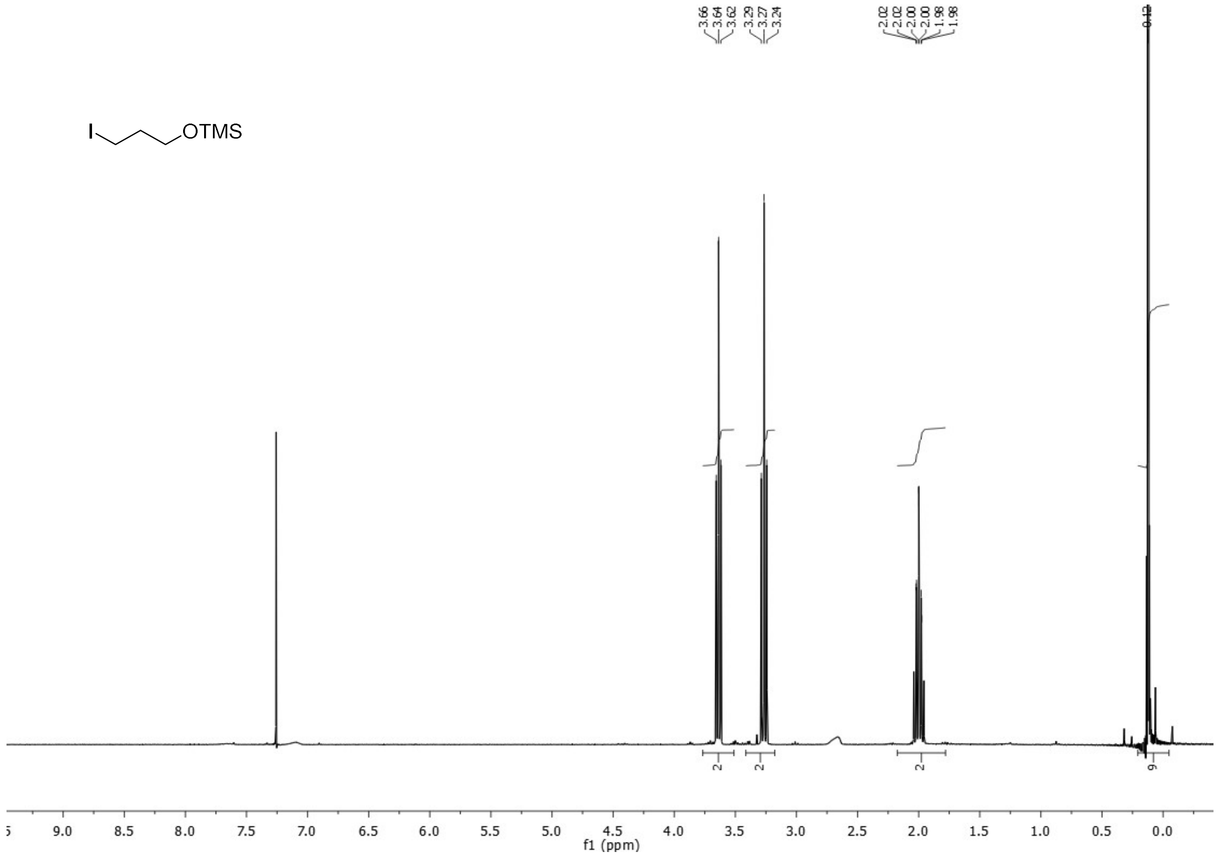

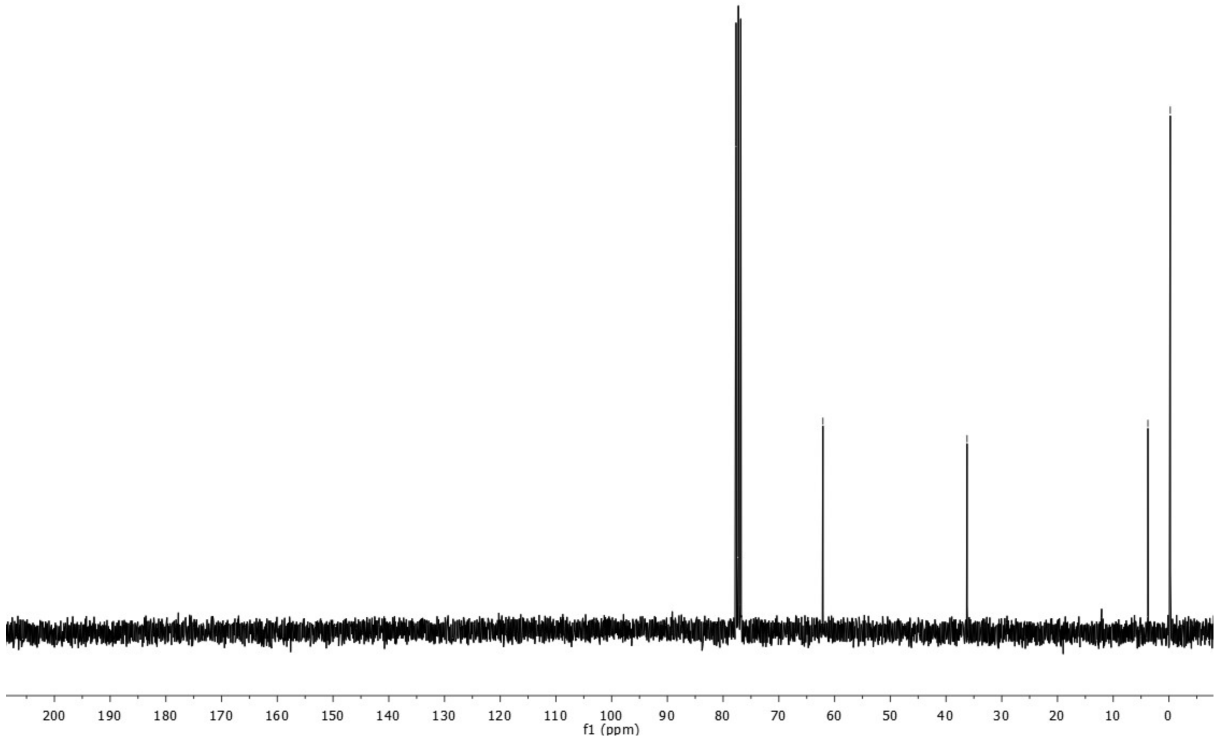

Fig. S28 ^{1}H NMR (300 MHz, $CDCl_3$) and ^{13}C NMR (75.5 MHz, $CDCl_3$) spectra of (3-iodopropoxy)trimethylsilane (**S7**).

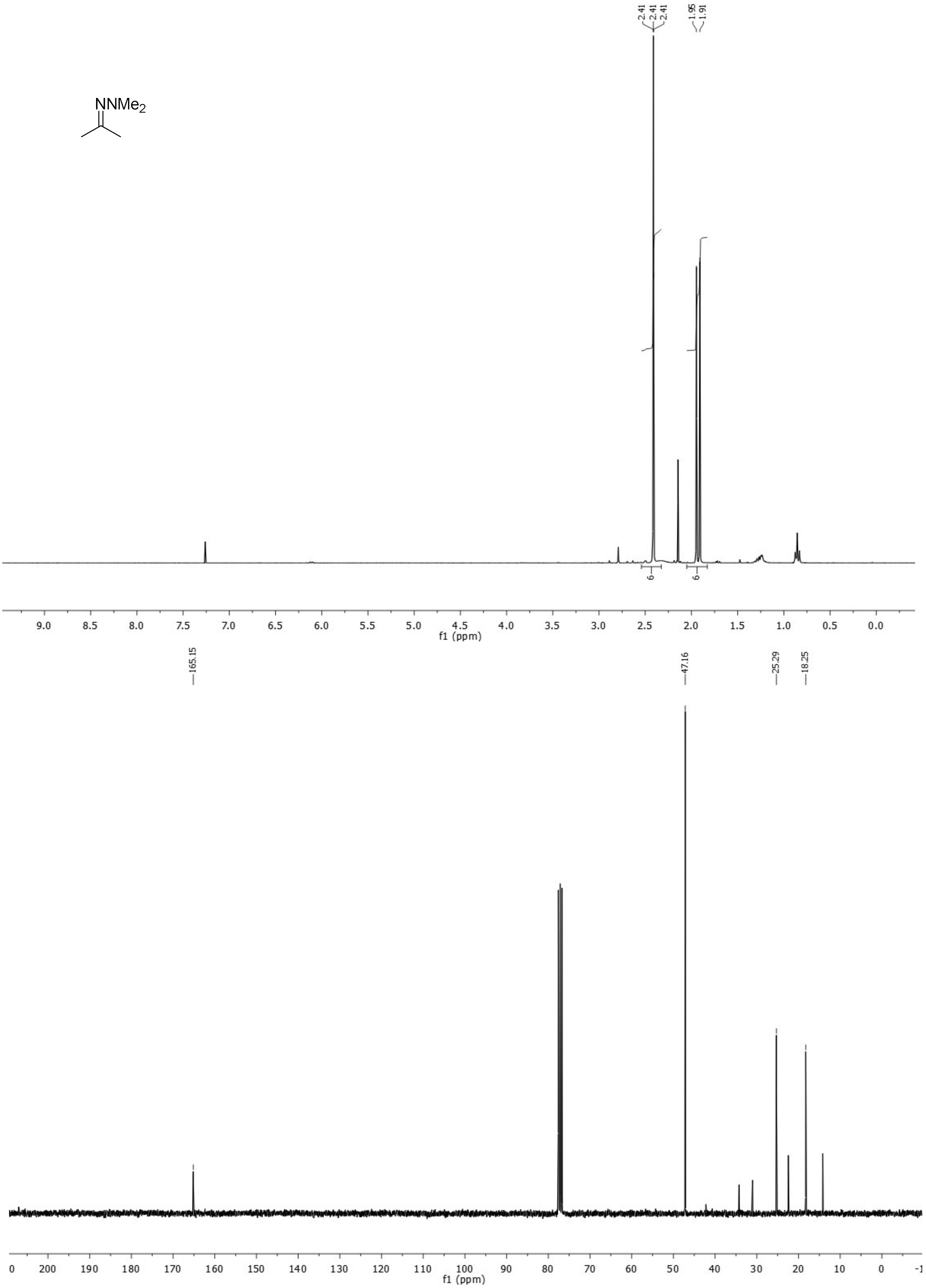

Fig. S29 ^{1}H NMR (300 MHz, $CDCl_3$) and ^{13}C NMR (75.5 MHz, $CDCl_3$) spectra of 1,1-dimethyl-2-(propan-2-ylidene)hydrazine (**S9**).

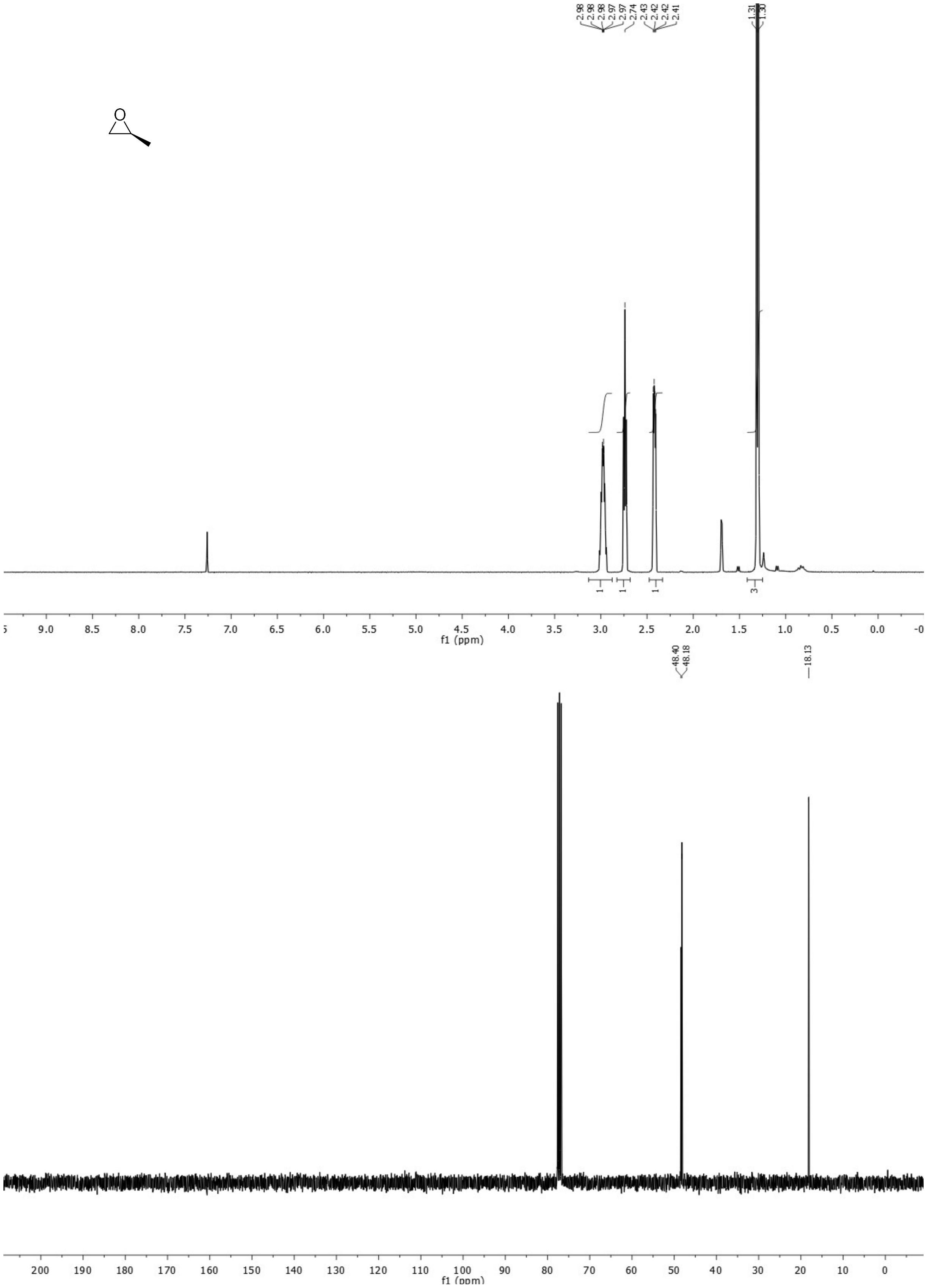

Fig. S30 ^{1}H NMR (300 MHz, CDCl$_3$) and ^{13}C NMR (75.5 MHz, CDCl$_3$) spectra of 1,1-dimethyl-2-(propan-2-ylidene)hydrazine (*S*)-**S10**).

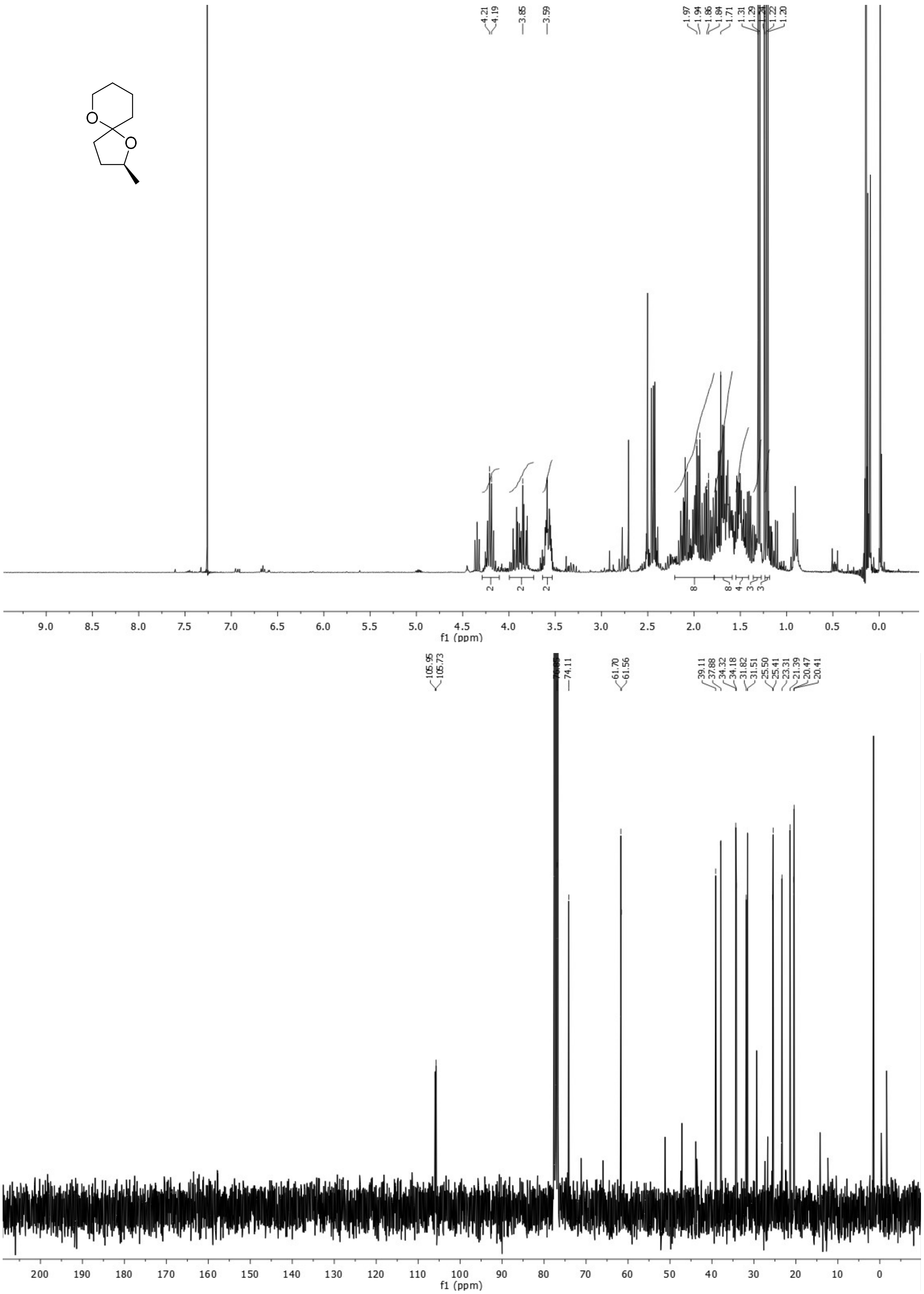

Fig. S31 ^{1}H NMR (300 MHz, $CDCl_3$) and ^{13}C NMR (75.5 MHz, $CDCl_3$) spectra of (2*S*, 5*SR*)-2-methyl-1,6-dioxaspiro[4.5]decane (**3**).

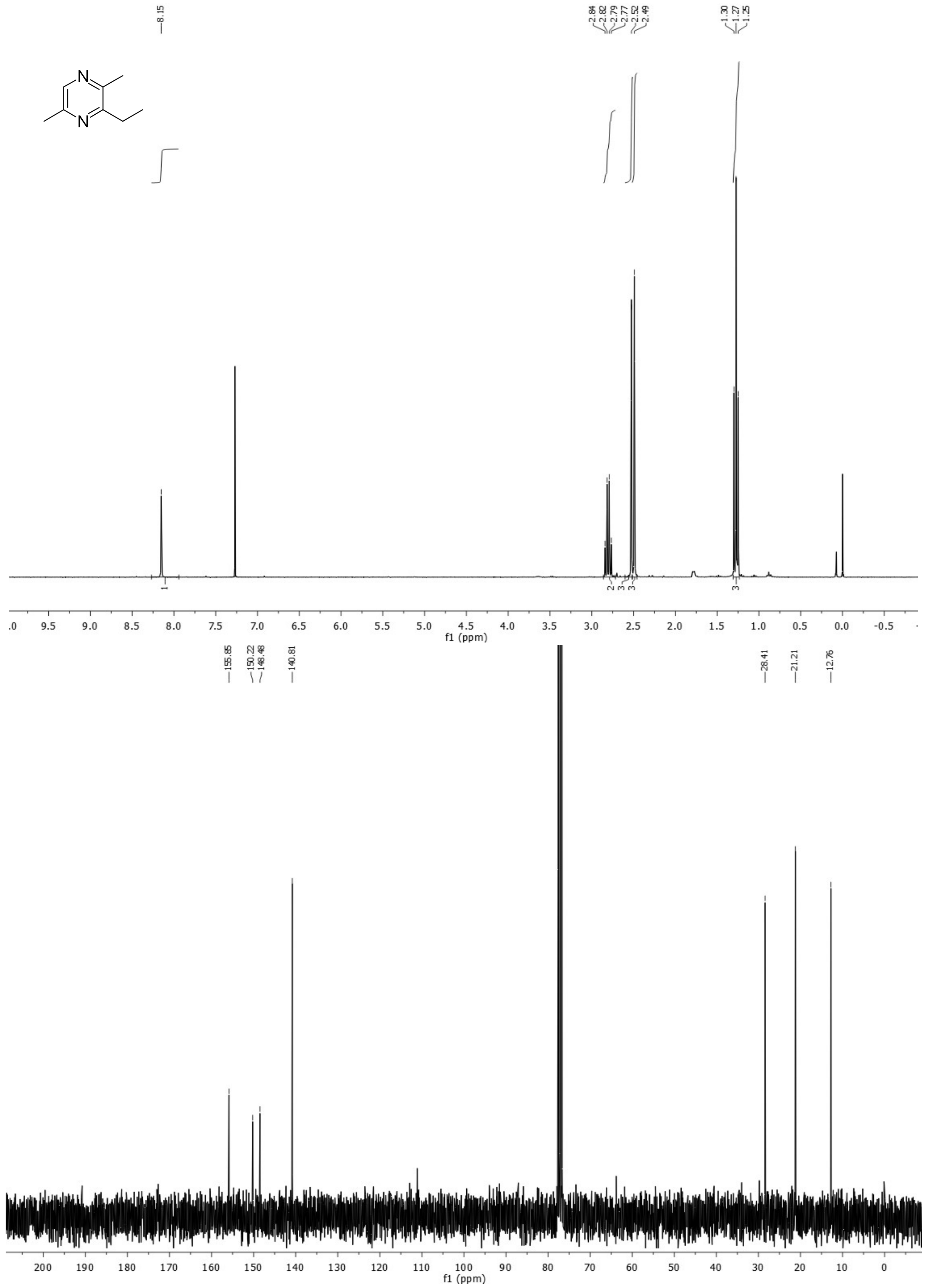

Fig. S32 ^{1}H NMR (300 MHz, $CDCl_3$) and ^{13}C NMR (75.5 MHz, $CDCl_3$) spectra of 3-ethyl-2,5-dimethylpyrazine (**4**).

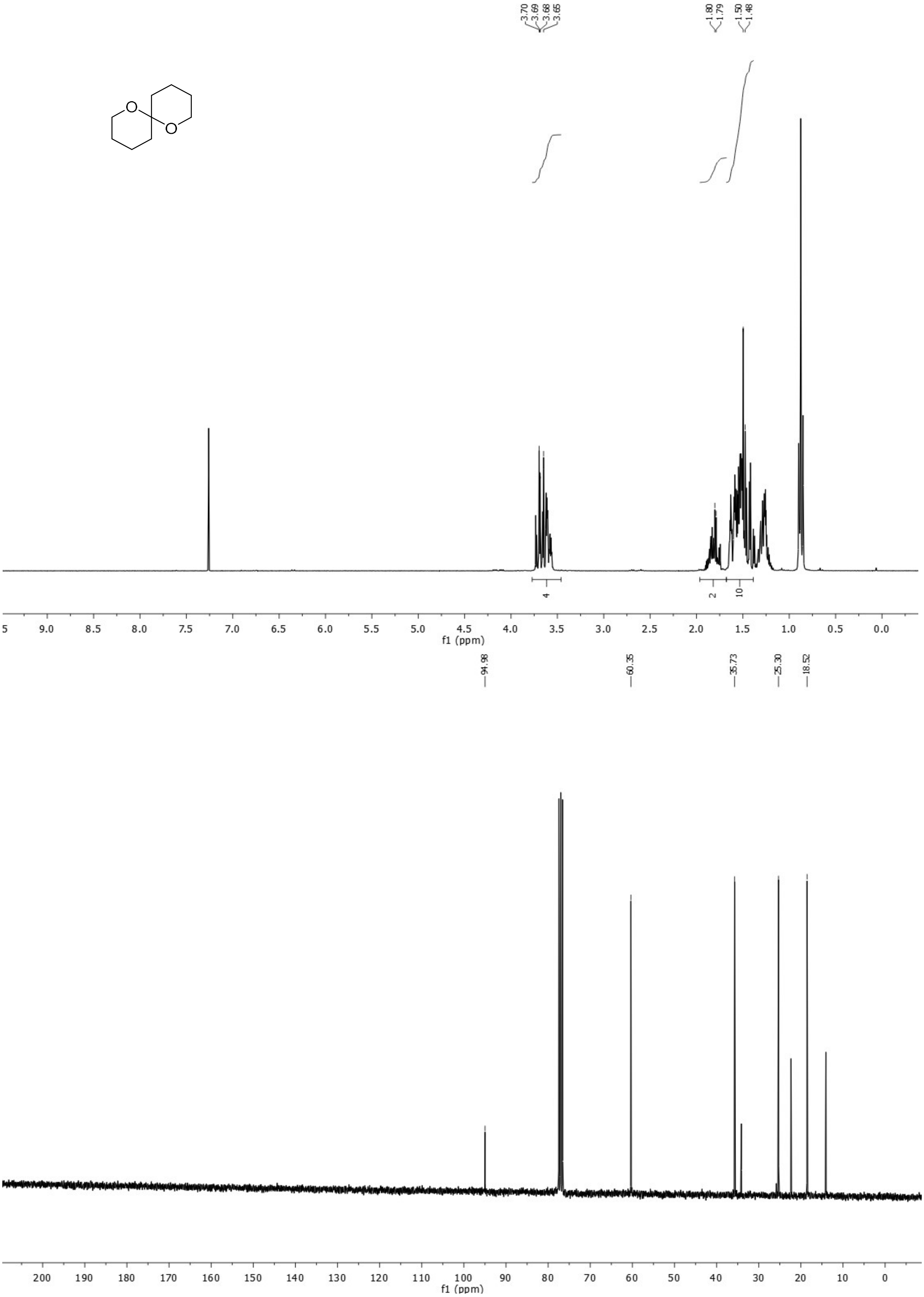

Fig. S33 ^{1}H NMR (300 MHz, $CDCl_3$) and ^{13}C NMR (75.5 MHz, $CDCl_3$) spectra of 1,7-dioxaspiro[5.5]undecane (**5**).

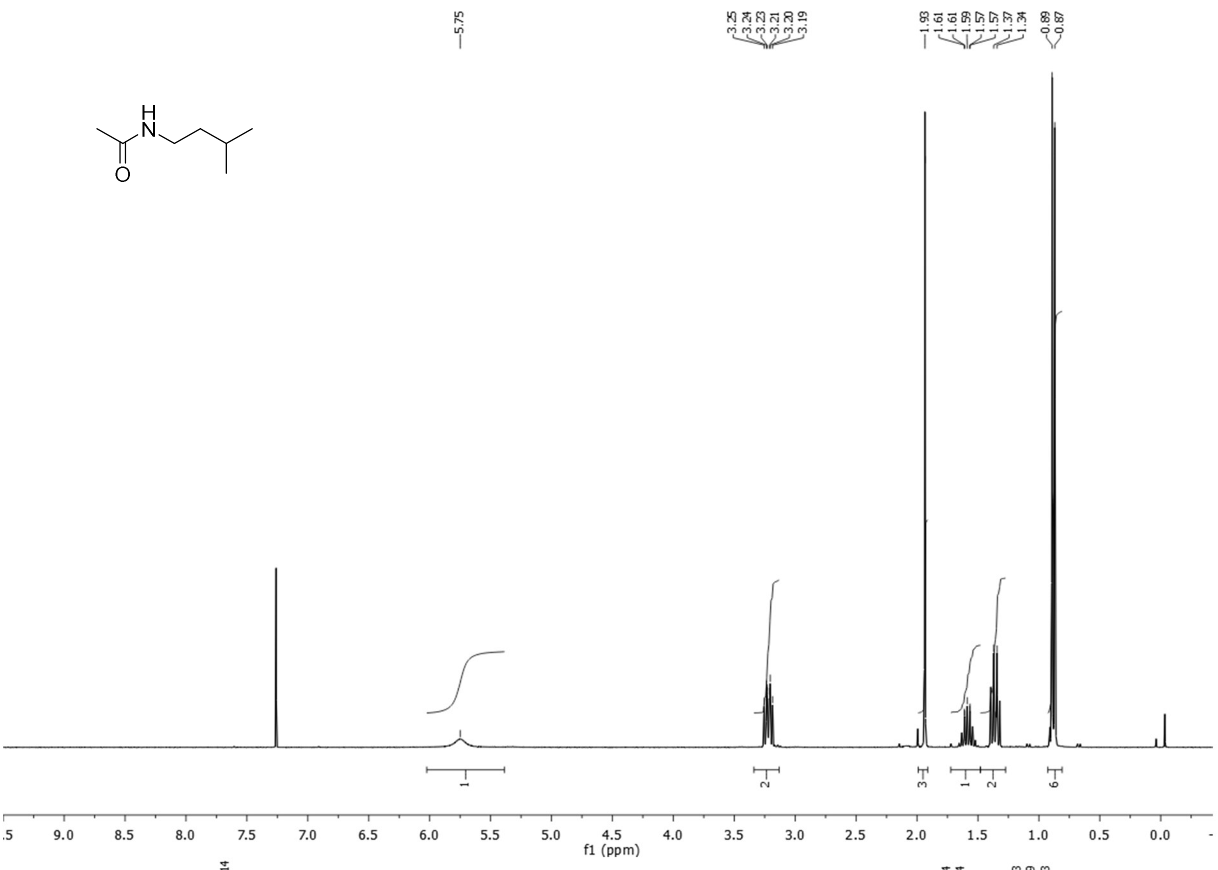

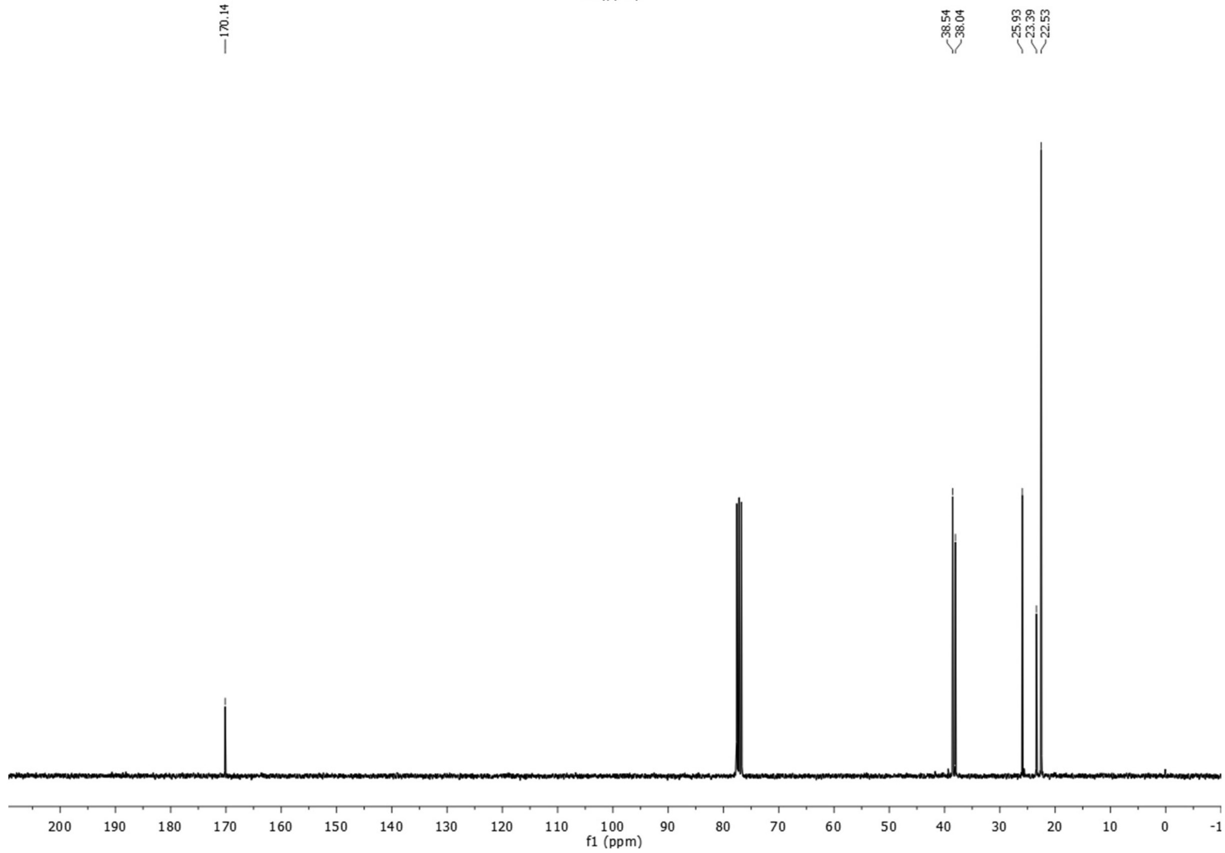

Fig. S34 ^{1}H NMR (300 MHz, $CDCl_3$) and ^{13}C NMR (75.5 MHz, $CDCl_3$) spectra of *N*-isopentylacetamide (**6**).

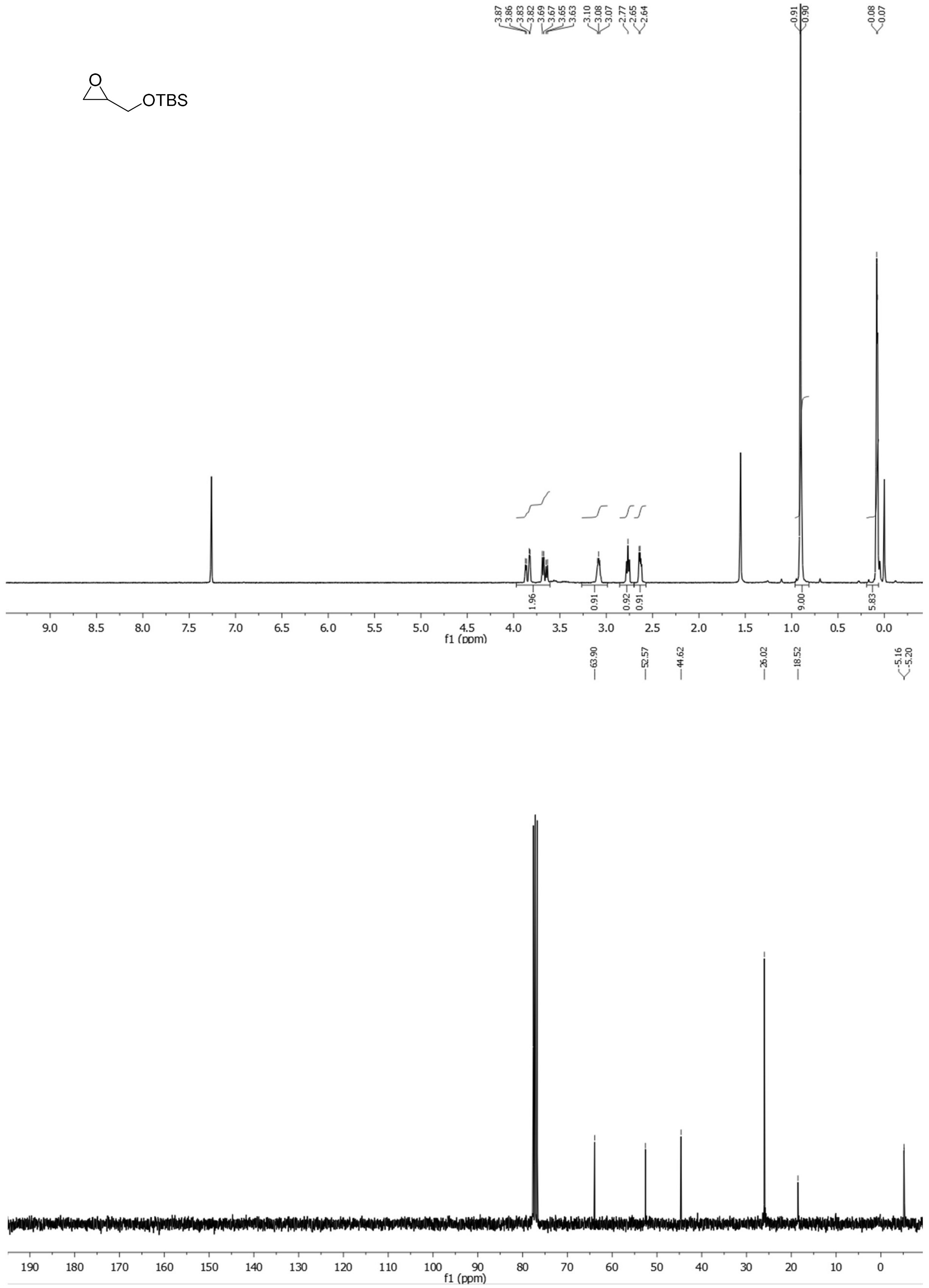

Fig. S35 ^{1}H NMR (300 MHz, $CDCl_3$) and ^{13}C NMR (75.5 MHz, $CDCl_3$) spectra of tert-butyldimethyl(oxiran-2-ylmethoxy)silane (**S17**).

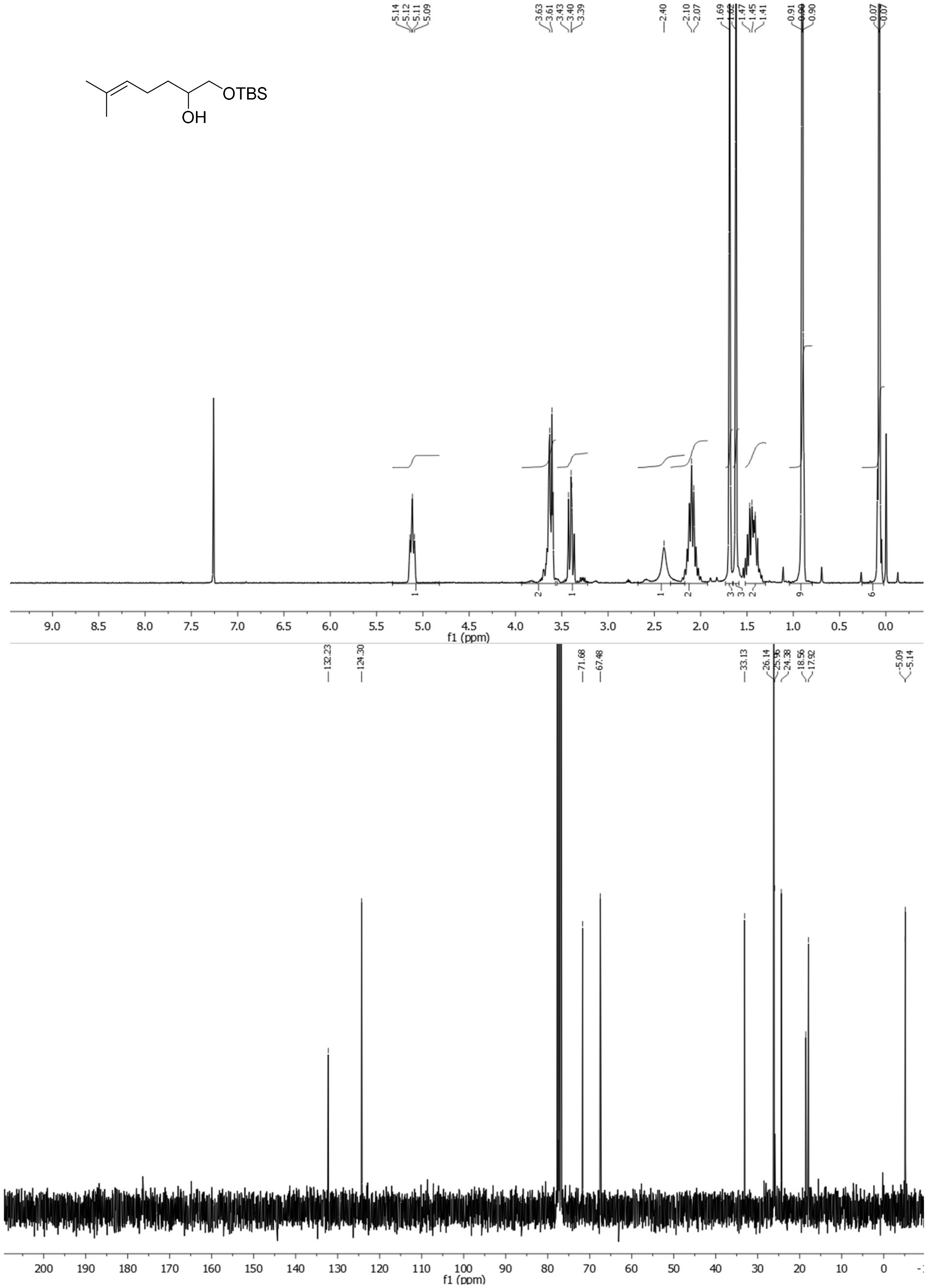

Fig. S36 ^{1}H NMR (300 MHz, $CDCl_3$) and ^{13}C NMR (75.5 MHz, $CDCl_3$) spectra of 1-((tert-butyldimethylsilyl)oxy)-6-methylhept-5-en-2-ol (**S19**).

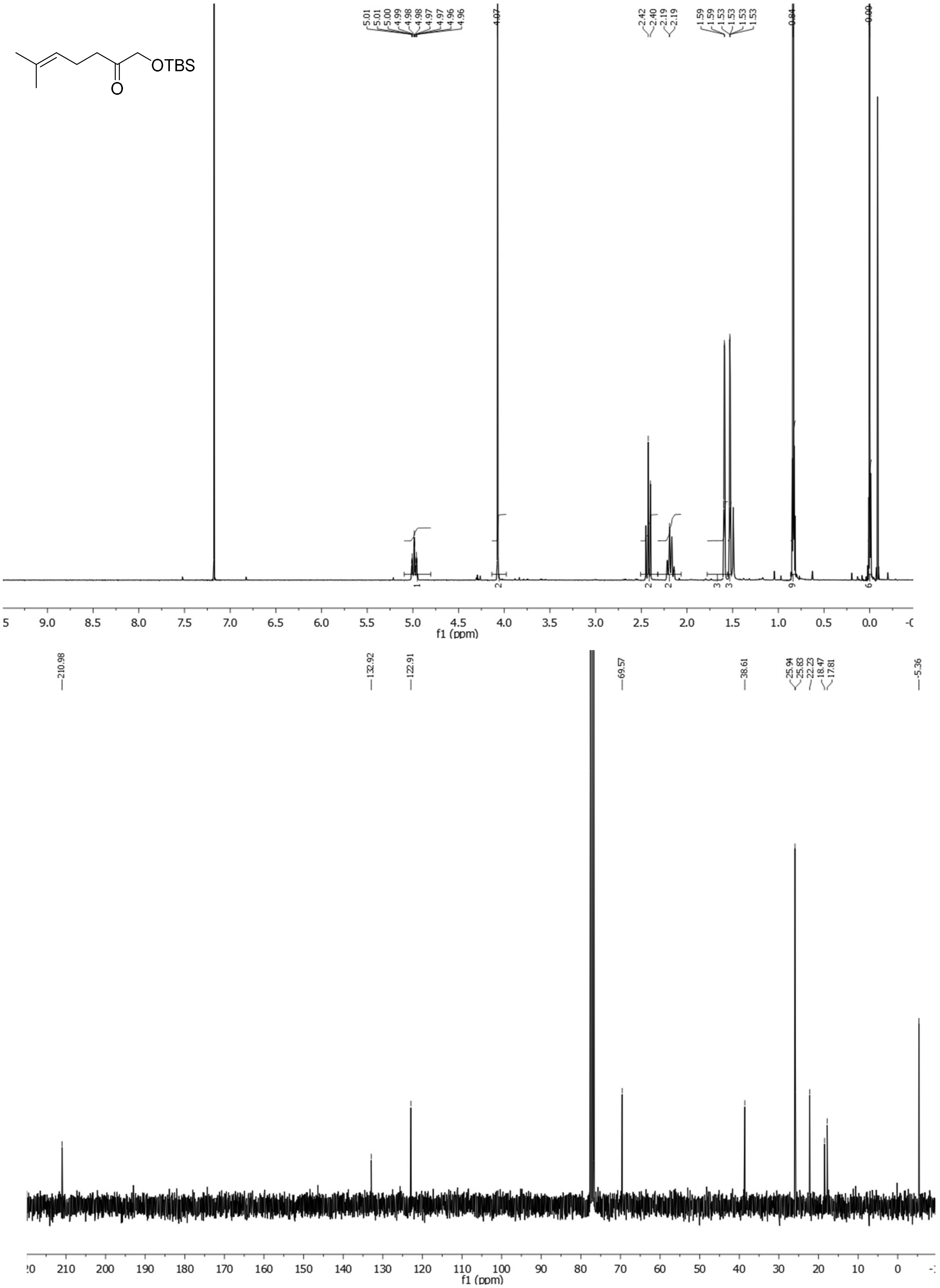

Fig. S37 ^{1}H NMR (300 MHz, $CDCl_3$) and ^{13}C NMR (75.5 MHz, $CDCl_3$) spectra of 1-((tert-butyldimethylsilyl)oxy)-6-methylhept-5-en-2-one (**S20**).

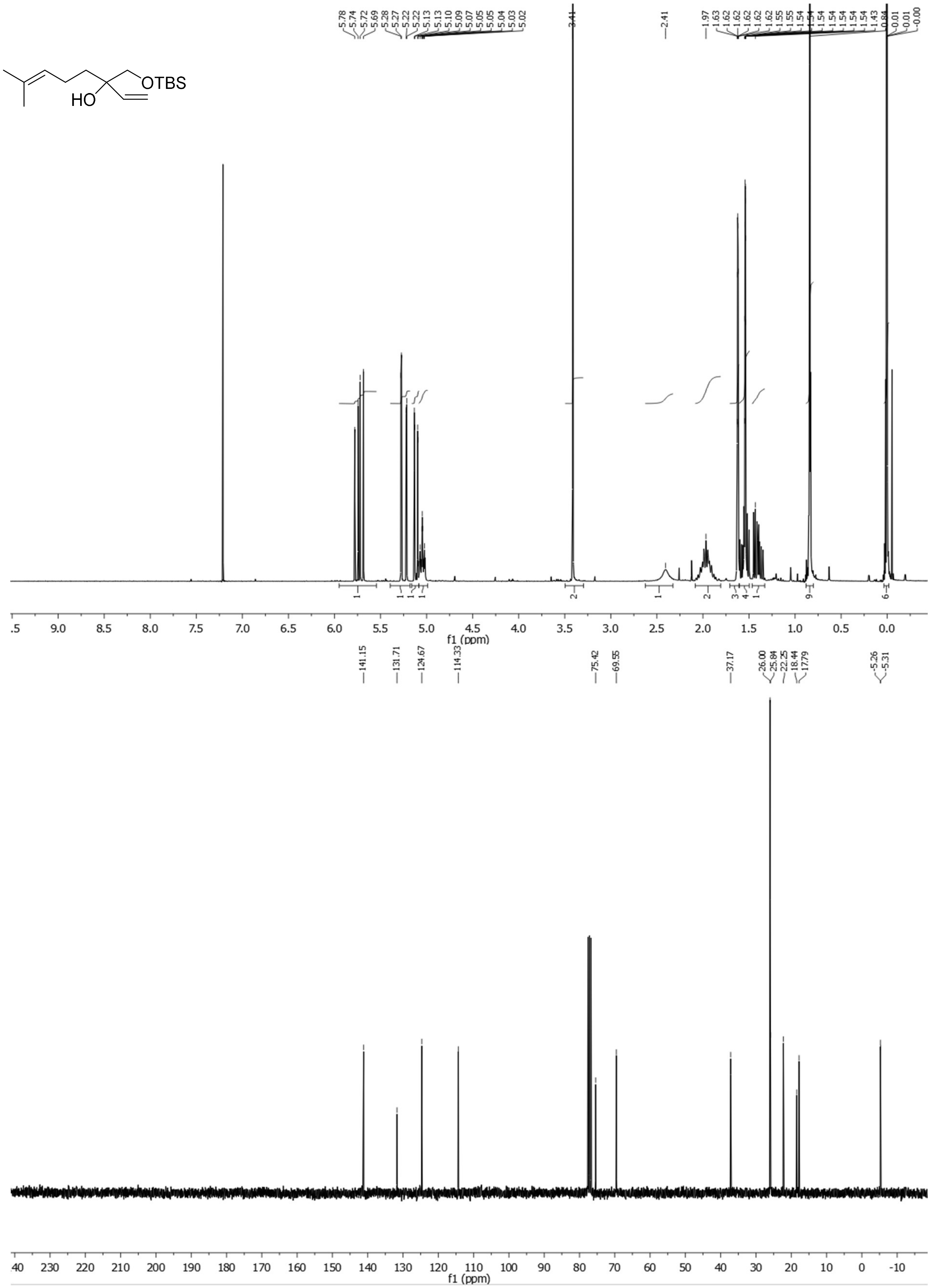

Fig. S38 ^{1}H NMR (300 MHz, $CDCl_3$) and ^{13}C NMR (75.5 MHz, $CDCl_3$) spectra of 3-(((tert-butyldimethylsilyl)oxy)methyl)-7-methylocta-1,6-dien-3-ol (**S21**).

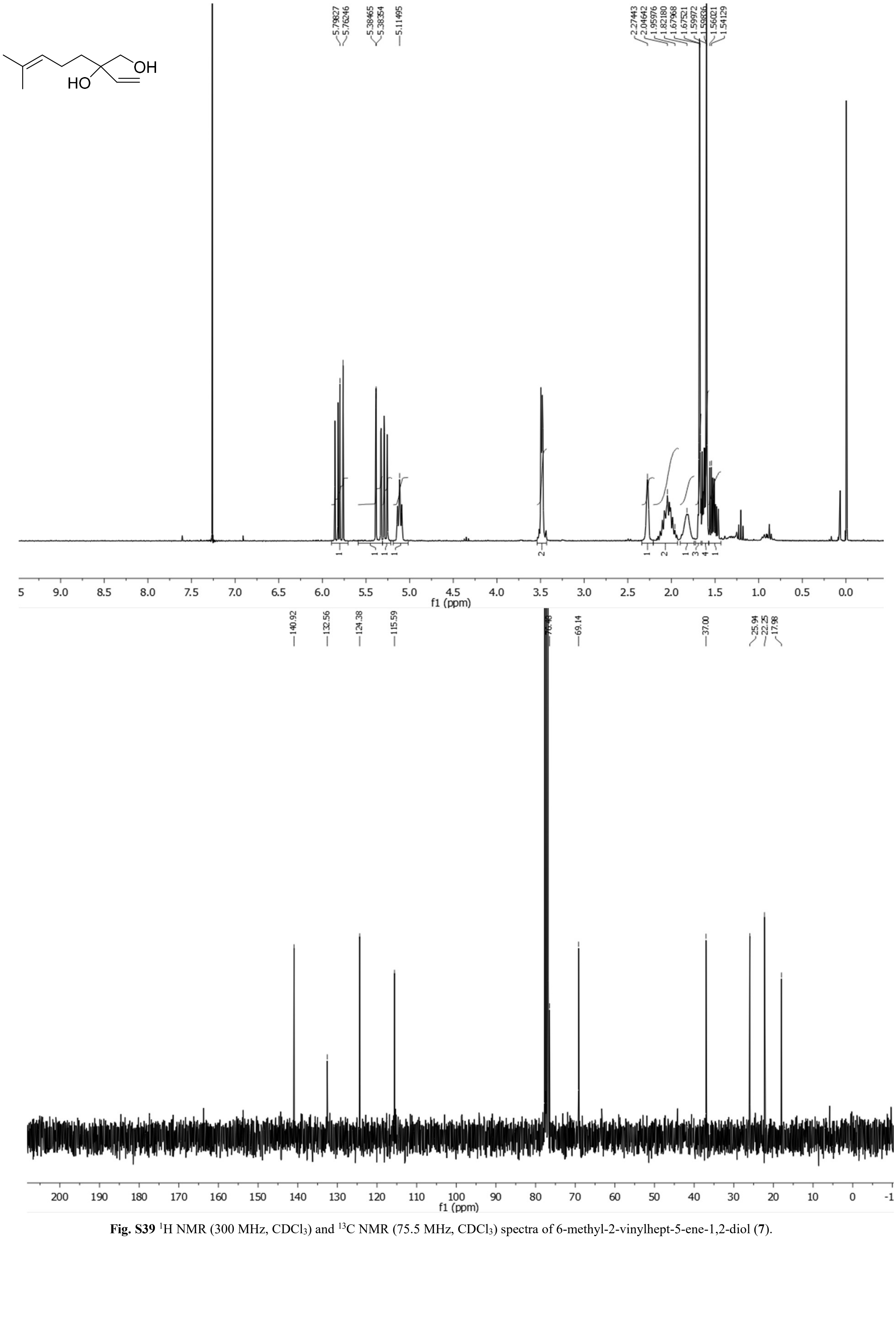

Fig. S39 ^{1}H NMR (300 MHz, $CDCl_3$) and ^{13}C NMR (75.5 MHz, $CDCl_3$) spectra of 6-methyl-2-vinylhept-5-ene-1,2-diol (**7**).

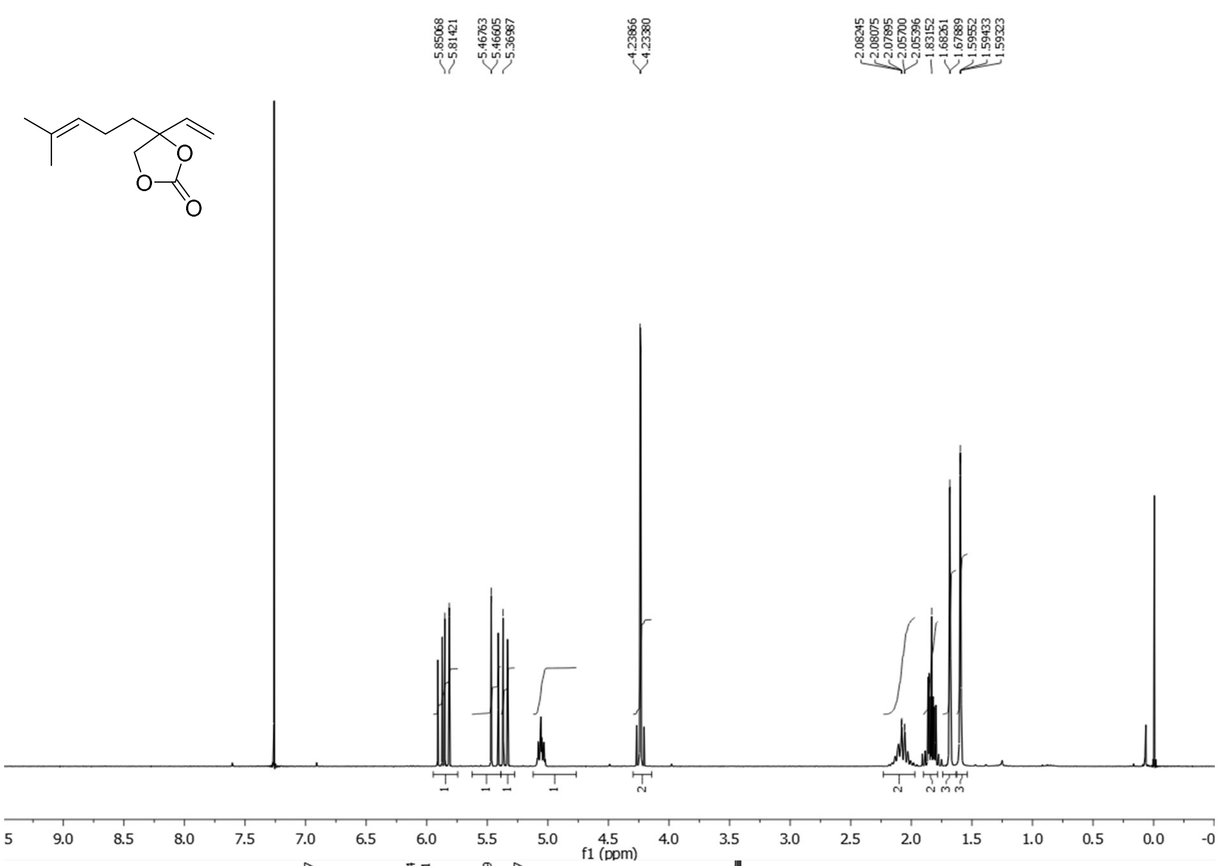

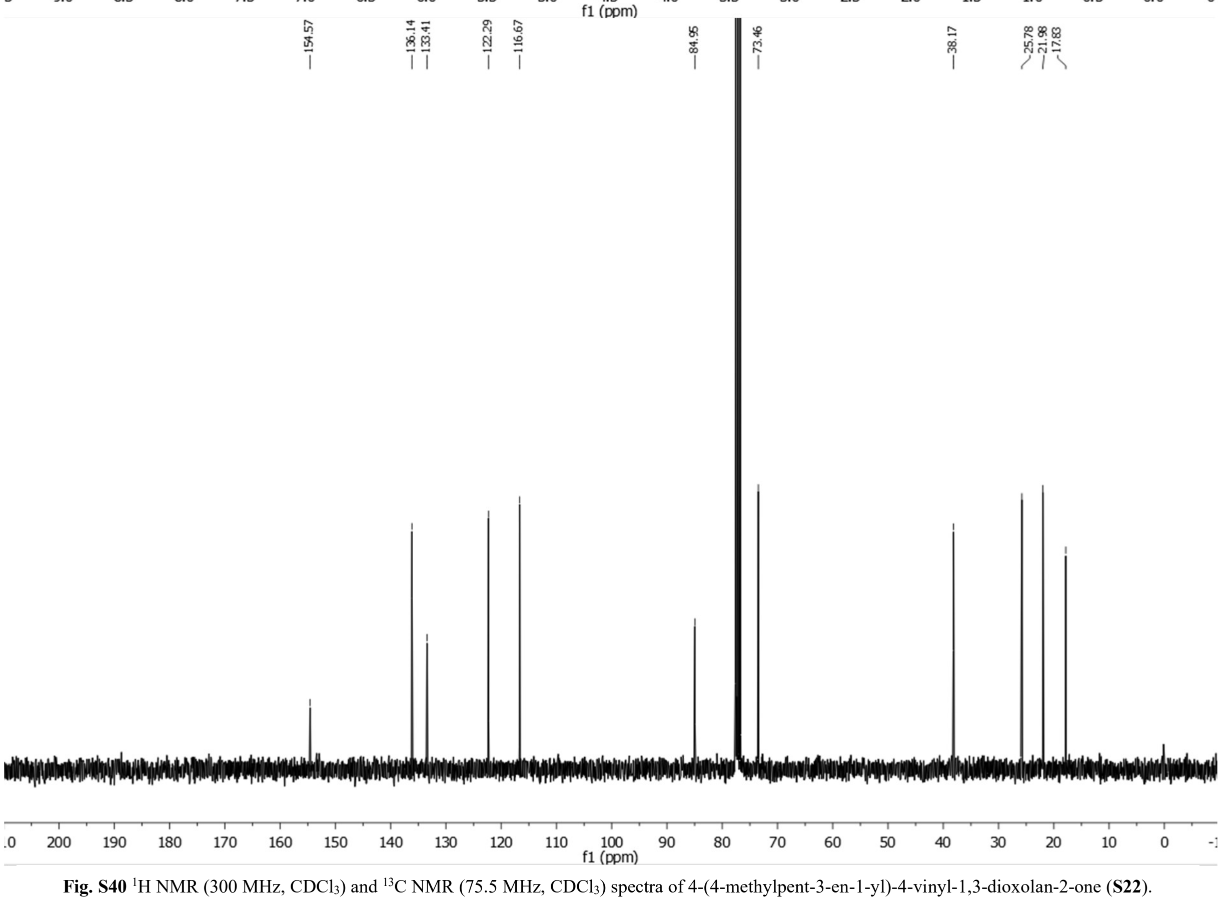

Fig. S40 ^{1}H NMR (300 MHz, $CDCl_3$) and ^{13}C NMR (75.5 MHz, $CDCl_3$) spectra of 4-(4-methylpent-3-en-1-yl)-4-vinyl-1,3-dioxolan-2-one (**S22**).

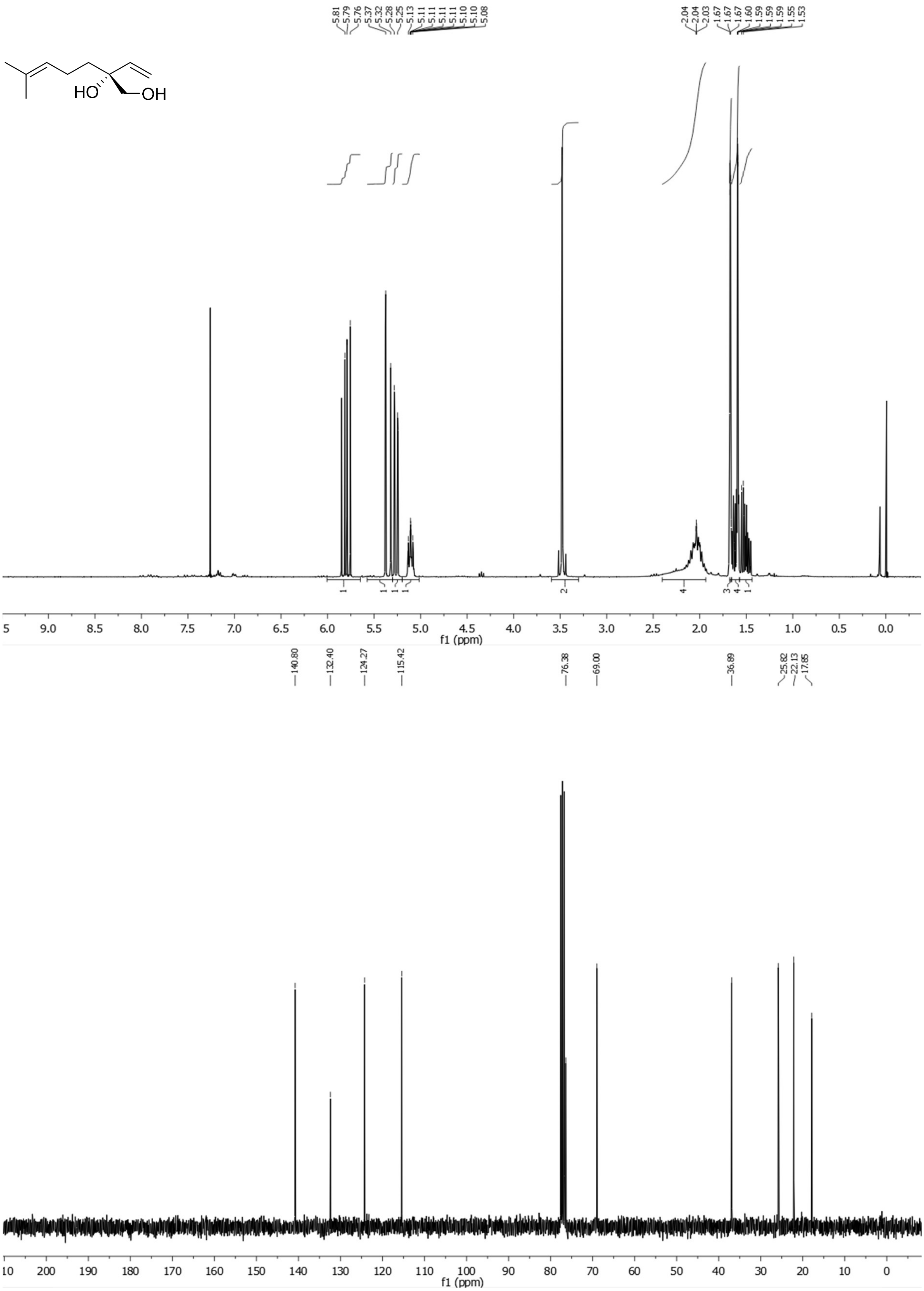

Fig. S41 ^{1}H NMR (300 MHz, $CDCl_3$) and ^{13}C NMR (75.5 MHz, $CDCl_3$) spectra of (*S*)-6-methyl-2-vinylhept-5-ene-1,2-diol ((*S*)-**7**).

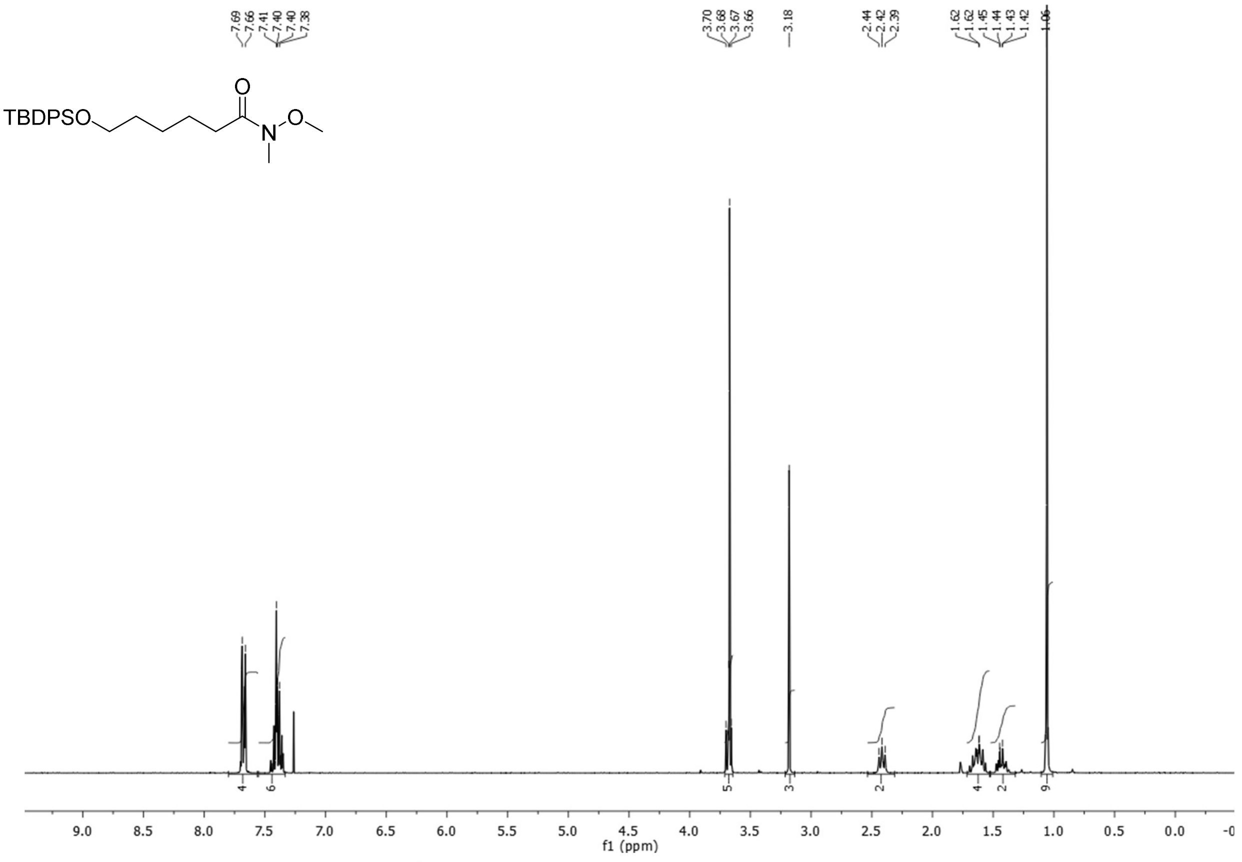

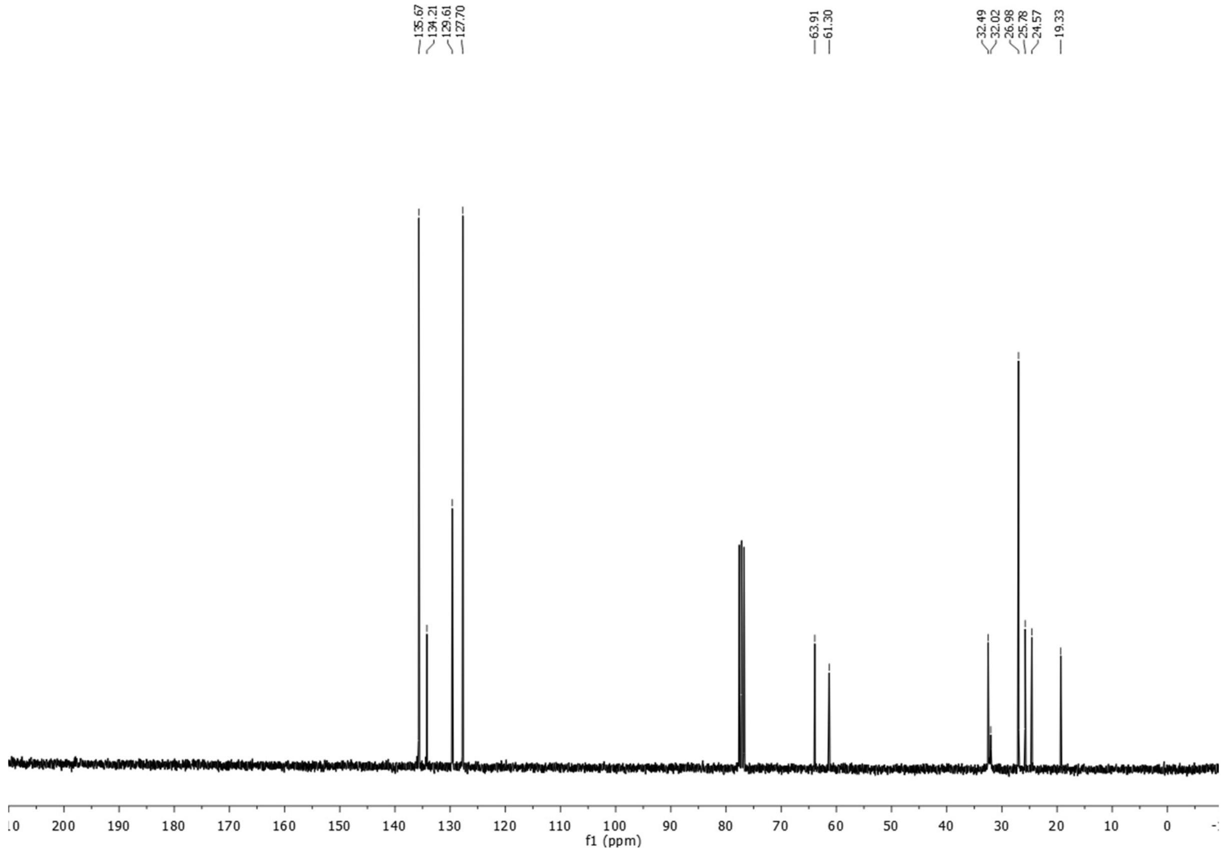

Fig. S42 ^{1}H NMR (300 MHz, $CDCl_3$) and ^{13}C NMR (75.5 MHz, $CDCl_3$) spectra of 6-((tert-butyldiphenylsilyl)oxy)-*N*-methoxy-*N*-methylhexanamide (**S24**).

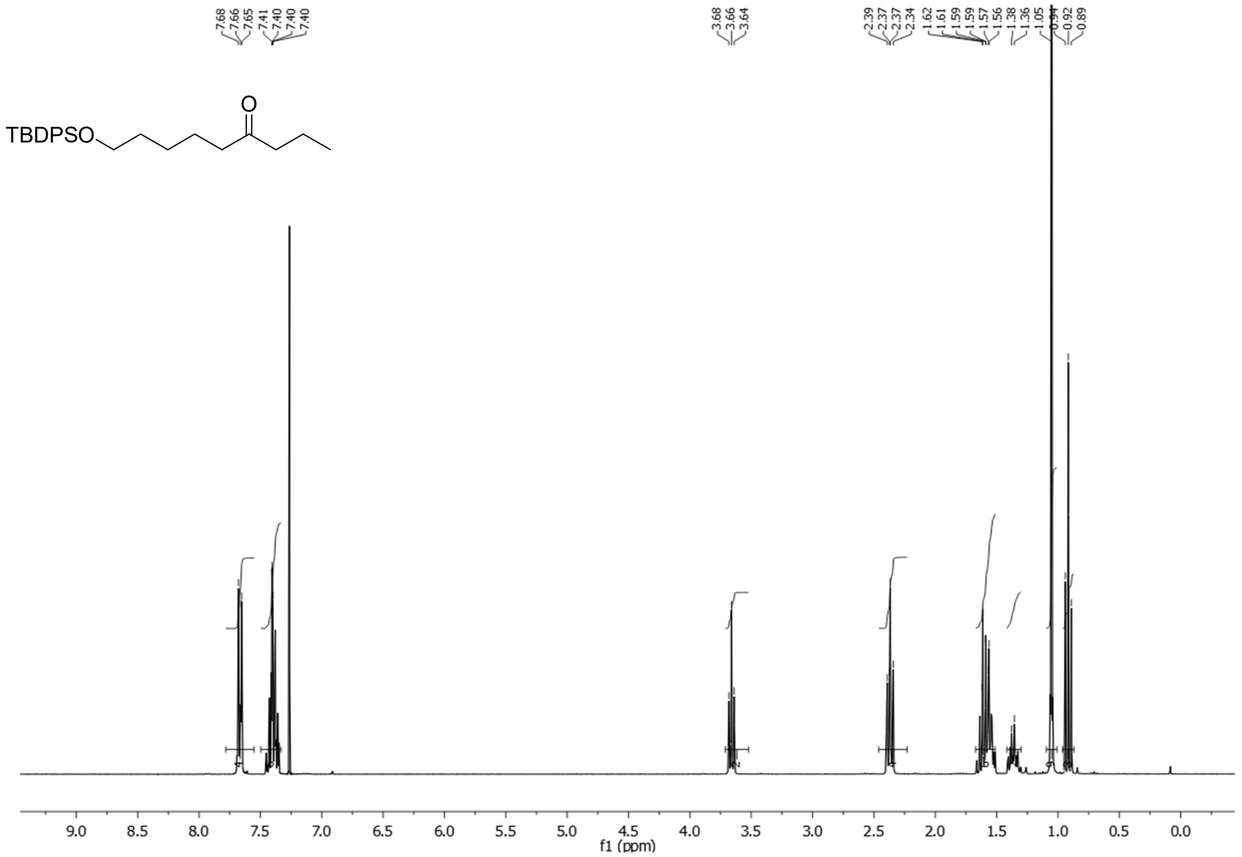

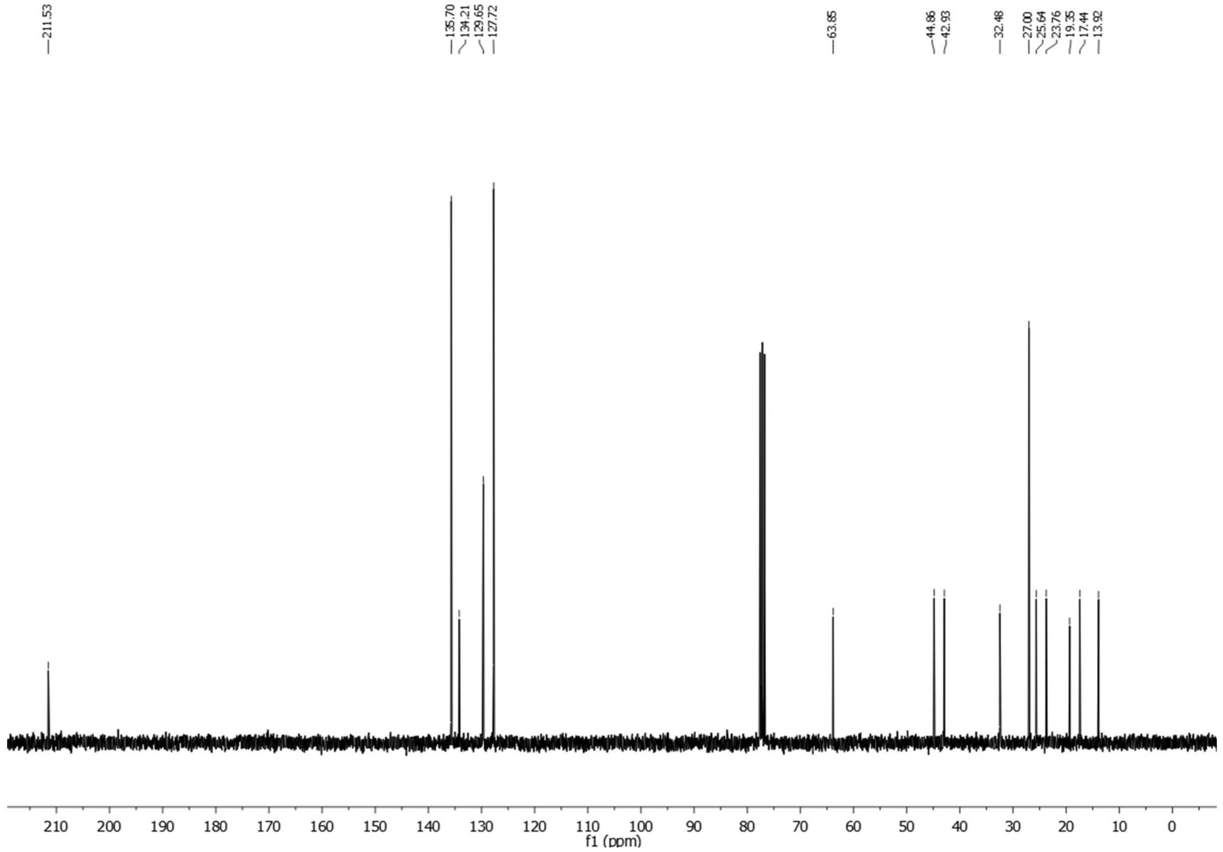

Fig. S43 ^{1}H NMR (300 MHz, $CDCl_3$) and ^{13}C NMR (75.5 MHz, $CDCl_3$) spectra of 9-((tert-butyldiphenylsilyl)oxy)nonan-4-one (**S25**).

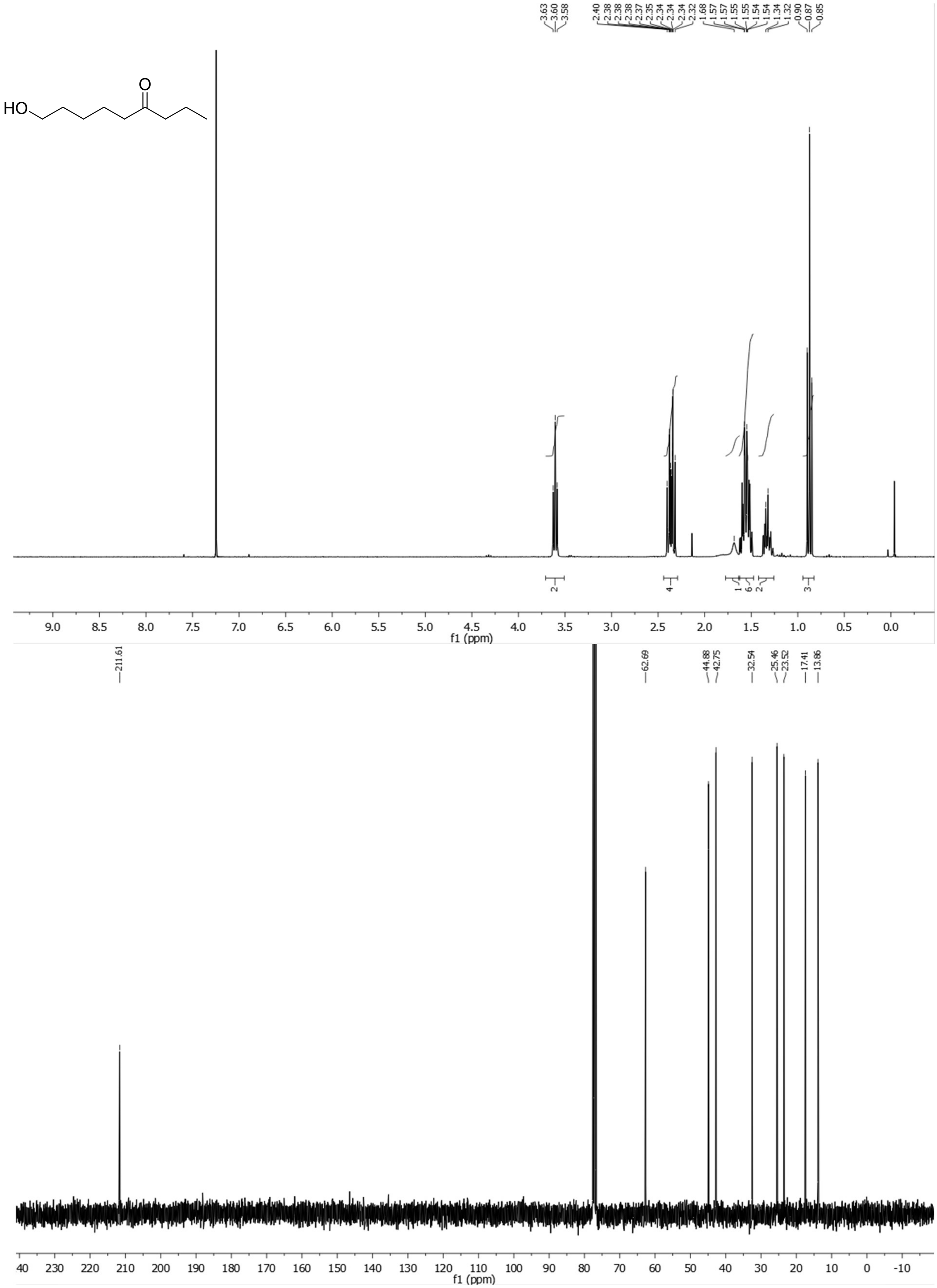

Fig. S44 ^{1}H NMR (300 MHz, $CDCl_3$) and ^{13}C NMR (75.5 MHz, $CDCl_3$) spectra of 6-oxo-nonan-1-ol (**8**).

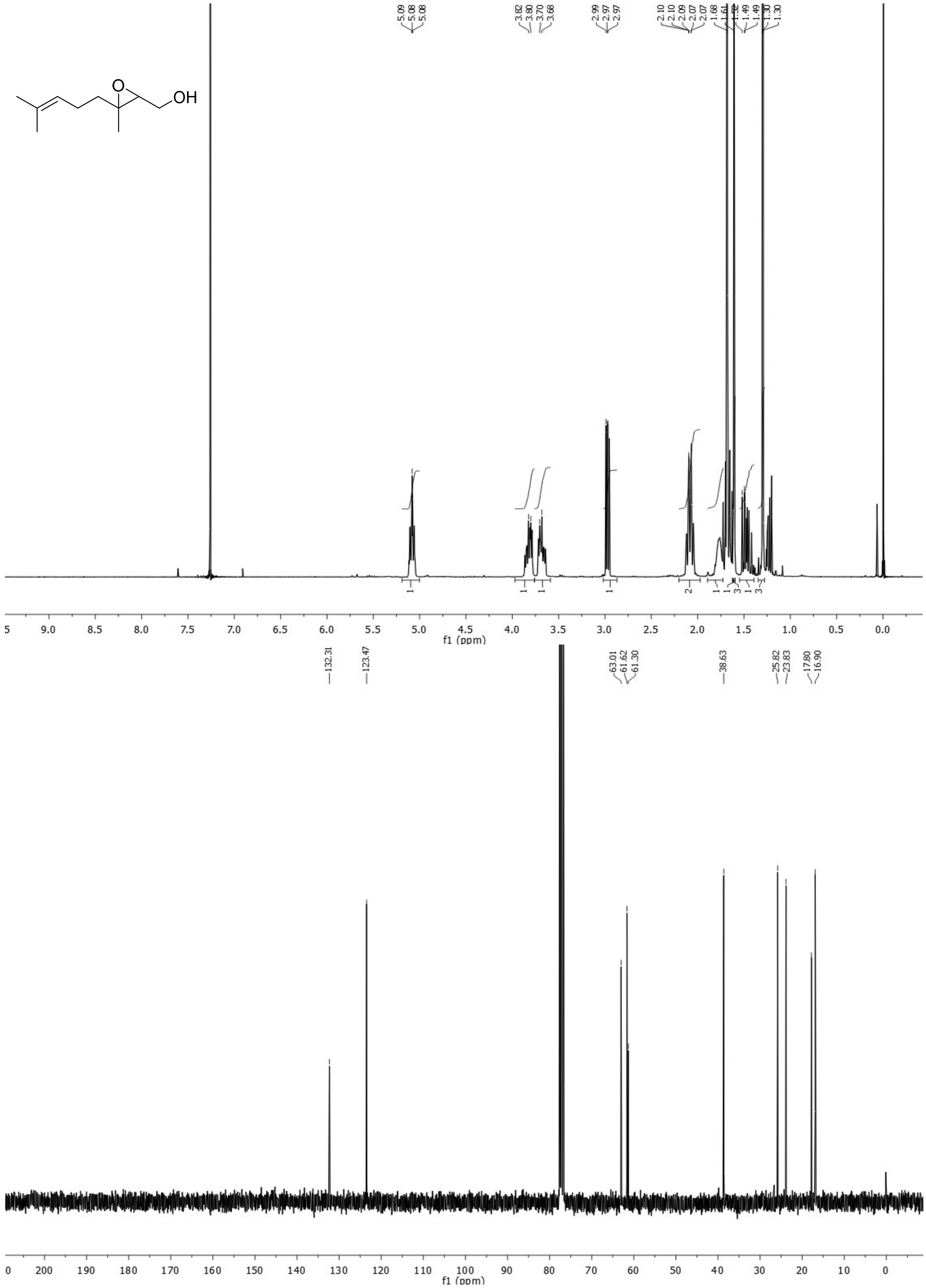

Fig. S45 ^{1}H NMR (300 MHz, $CDCl_3$) and ^{13}C NMR (75.5 MHz, $CDCl_3$) spectra of (3-methyl-3-(4-methylpent-3-en-1-yl)oxiran-2-yl)methanol (**S27**).

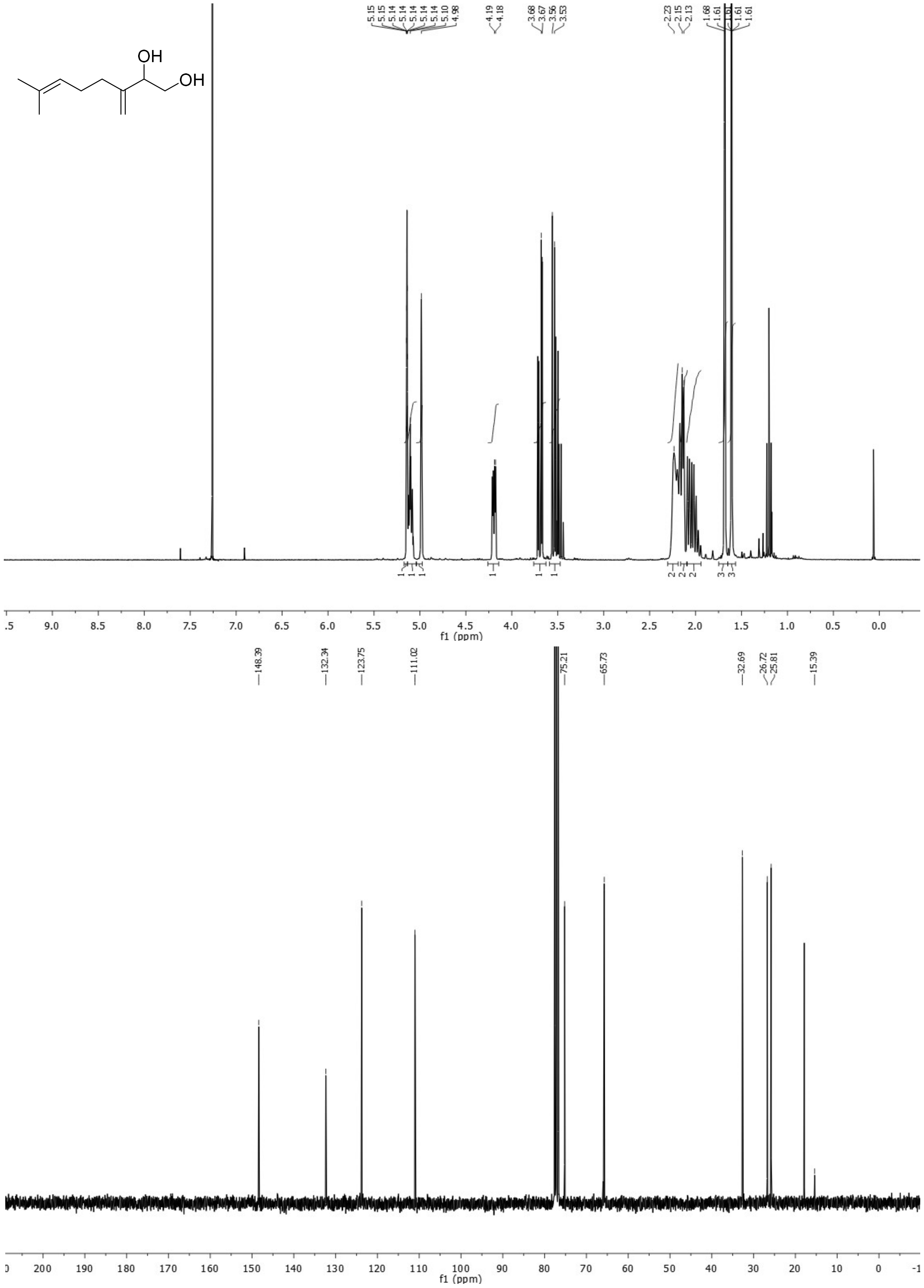

Fig. S46 ^{1}H NMR (300 MHz, $CDCl_3$) and ^{13}C NMR (75.5 MHz, $CDCl_3$) spectra of 7-methyl-3-methyleneoct-6-ene-1,2-diol (**9**).

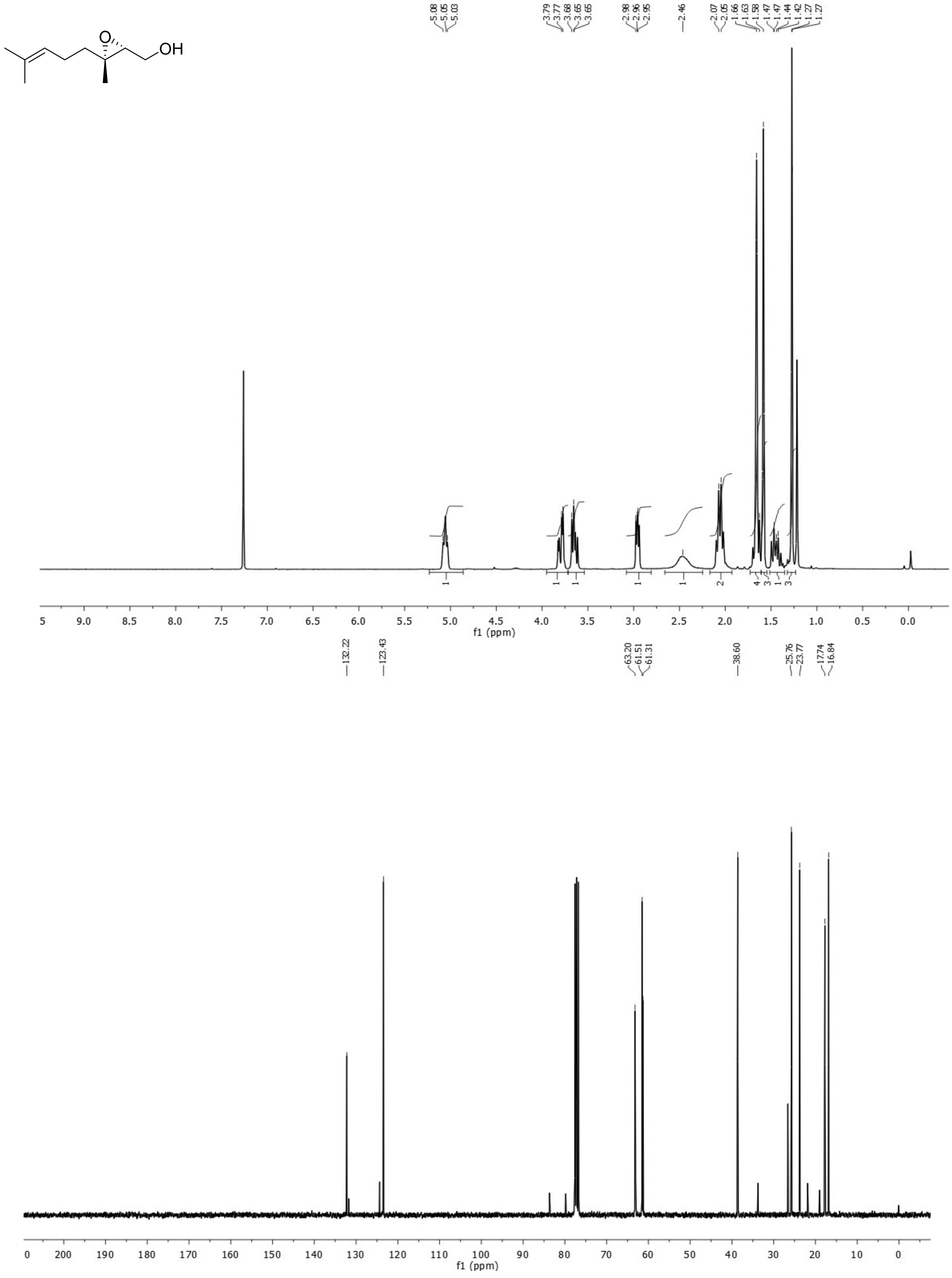

Fig. S47 ^{1}H NMR (300 MHz, $CDCl_3$) and ^{13}C NMR (75.5 MHz, $CDCl_3$) spectra of ((2*R*,3*R*)-3-methyl-3-(4-methylpent-3-en-1-yl)oxiran-2-yl)methanol ((2*R*,3*R*)-**S27**).

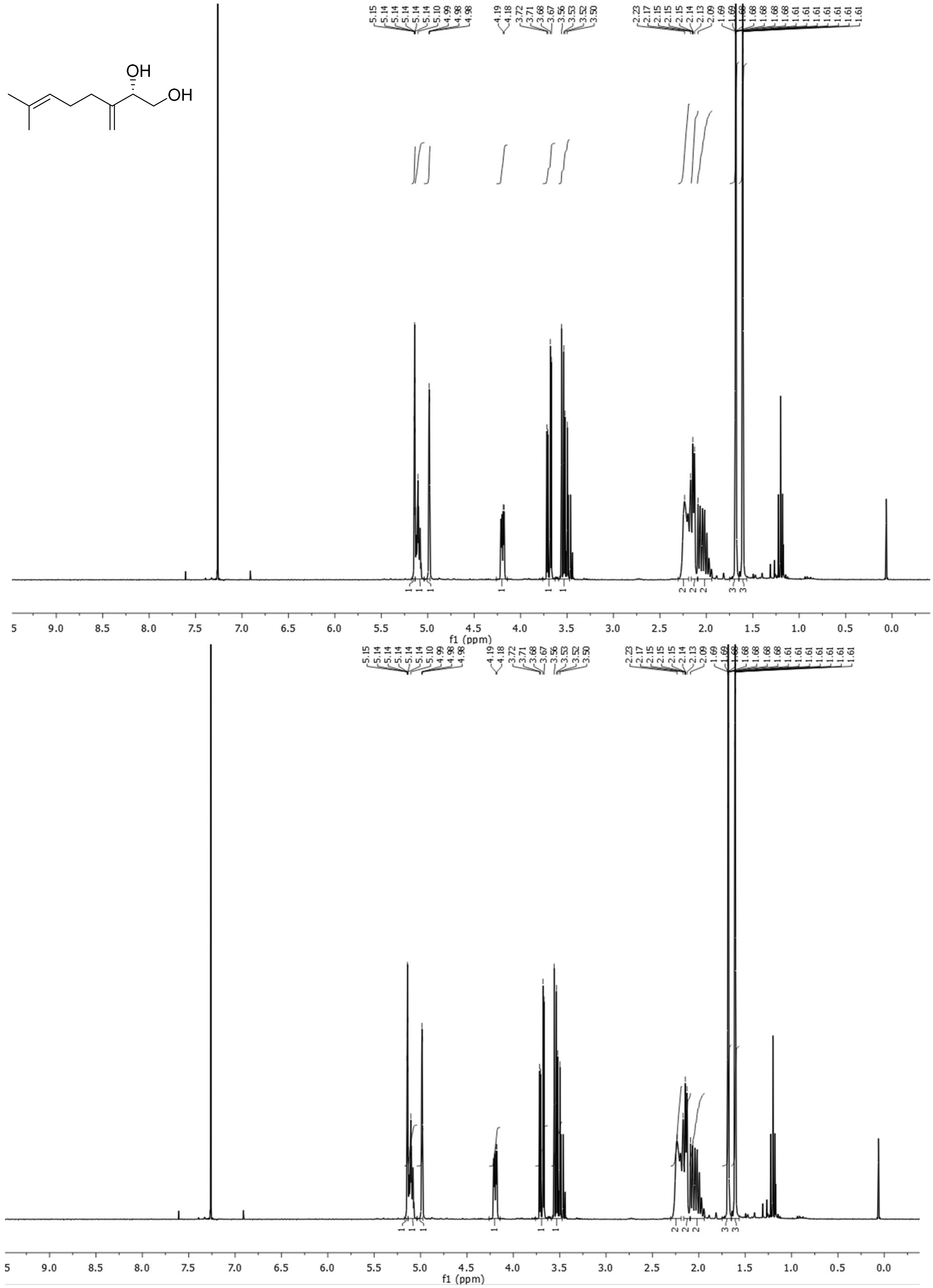

Fig. S48 ^{1}H NMR (300 MHz, $CDCl_3$) and ^{13}C NMR (75.5 MHz, $CDCl_3$) spectra of (*S*)-7-methyl-3-methyleneoct-6-ene-1,2-diol ((*S*)-**9**).

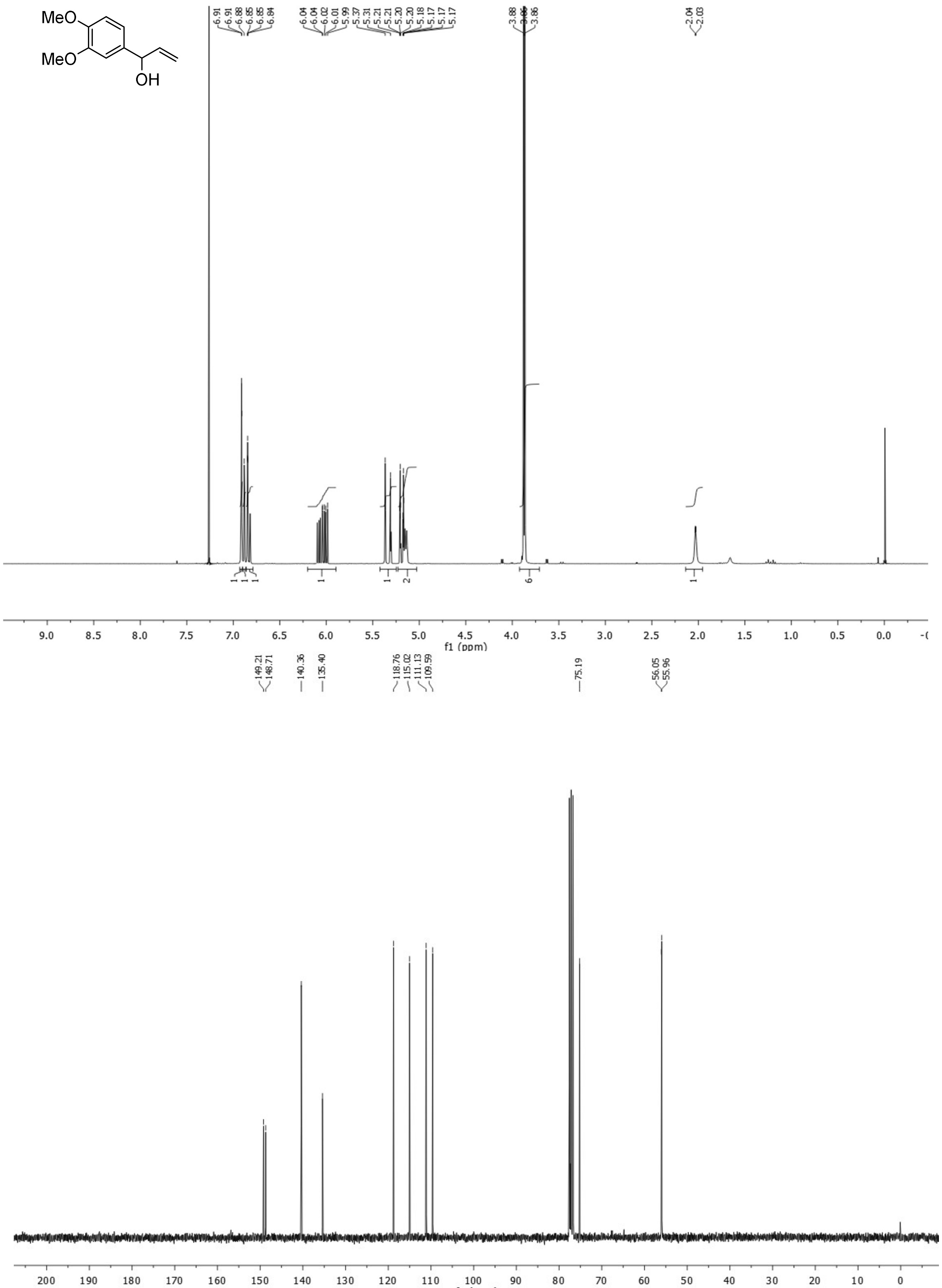

Fig. S49 ^{1}H NMR (300 MHz, $CDCl_3$) and ^{13}C NMR (75.5 MHz, $CDCl_3$) spectra of 1-(3,4-dimethoxyphenyl)prop-2-en-1-ol (**12**).

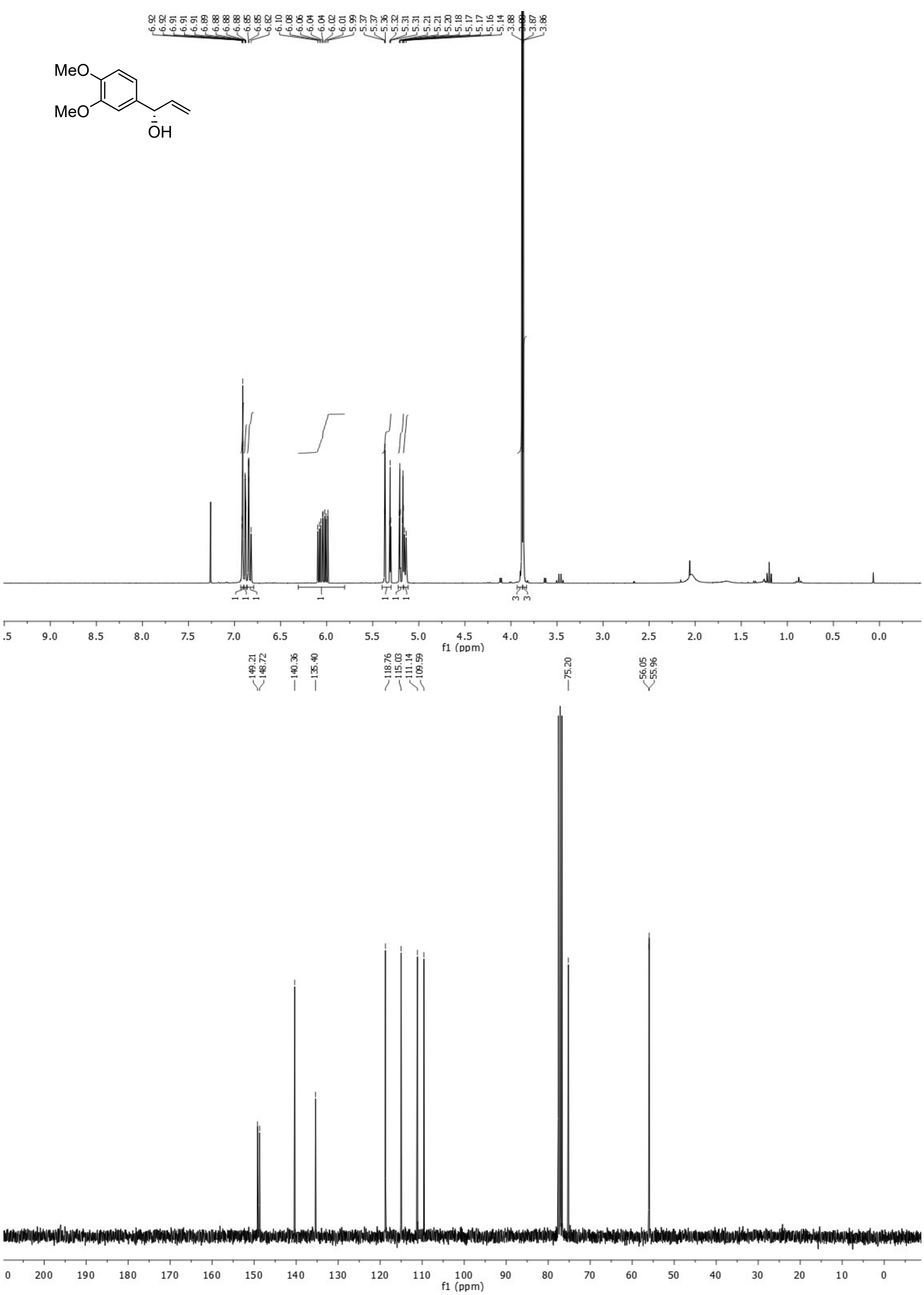

Fig. S50 ^{1}H NMR (300 MHz, $CDCl_3$) and ^{13}C NMR (75.5 MHz, $CDCl_3$) spectra of (*S*)-1-(3,4-dimethoxyphenyl)prop-2-en-1-ol ((*S*)-**12**).

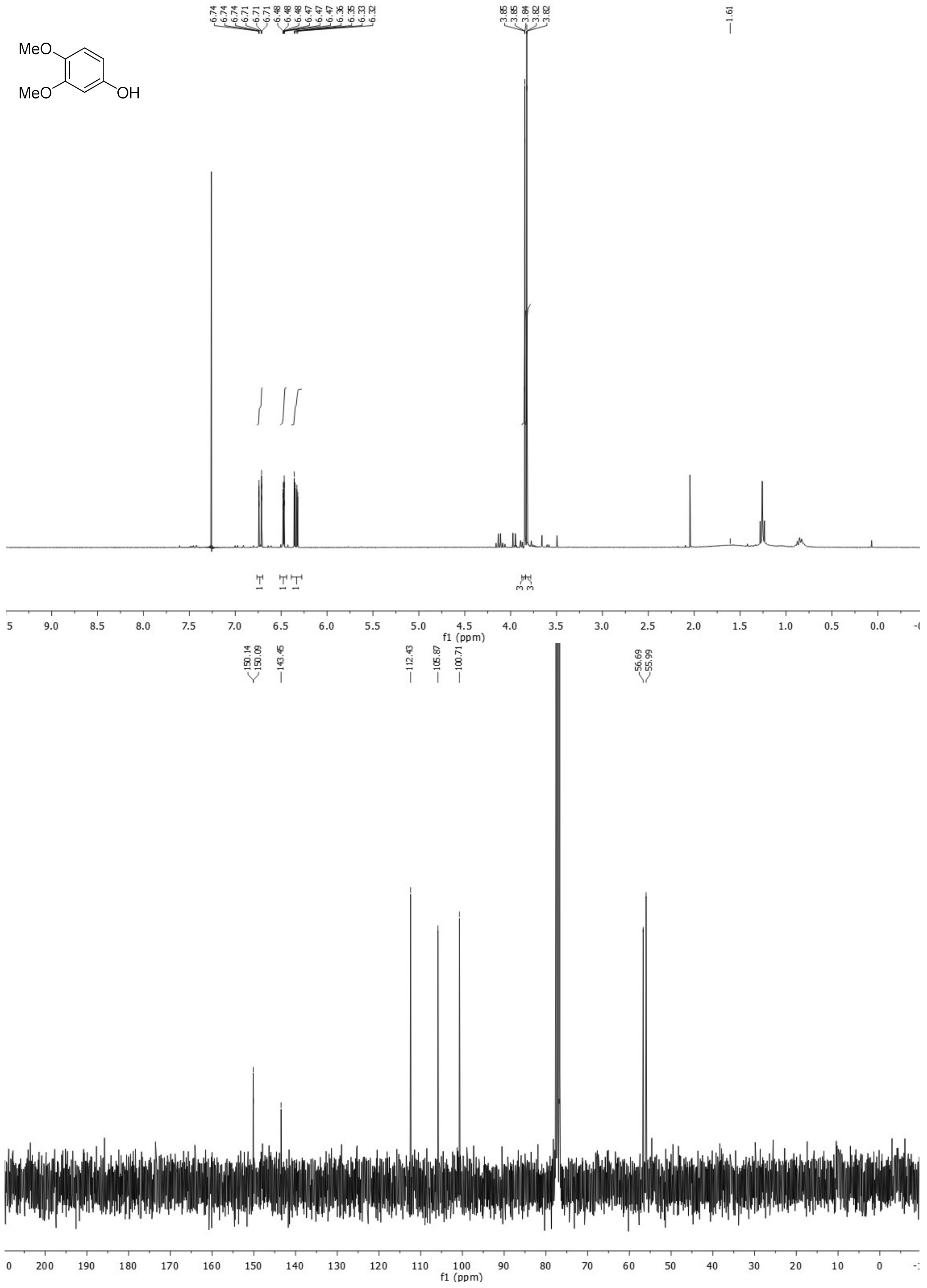

Fig. S51 ^{1}H NMR (300 MHz, $CDCl_3$) and ^{13}C NMR (75.5 MHz, $CDCl_3$) spectra of ethyl 3,4-dimethoxyphenol (**S29**).

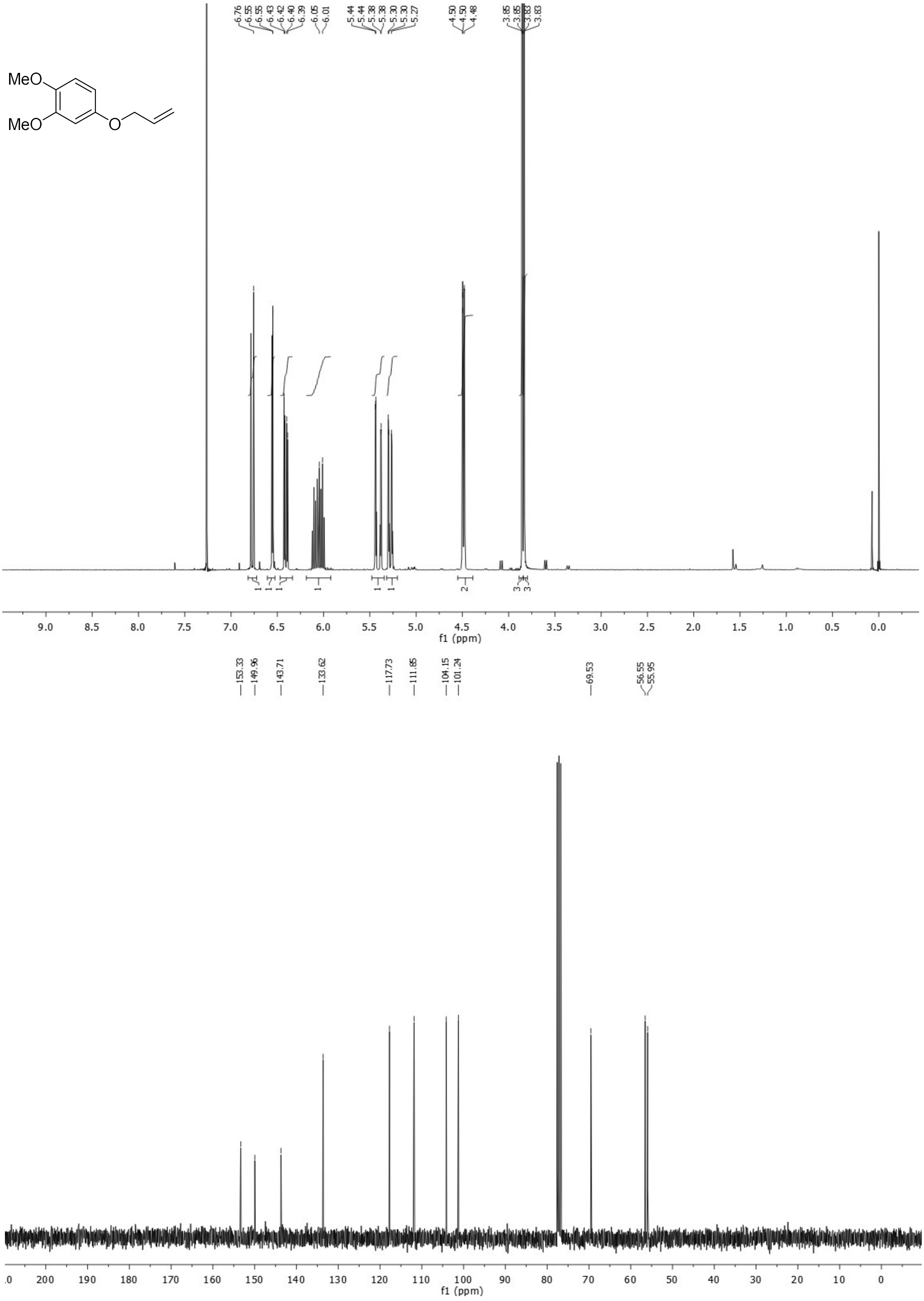

Fig. S52 ^{1}H NMR (300 MHz, $CDCl_3$) and ^{13}C NMR (75.5 MHz, $CDCl_3$) spectra of 4-(allyloxy)-1,2-dimethoxybenzene (**S30**).

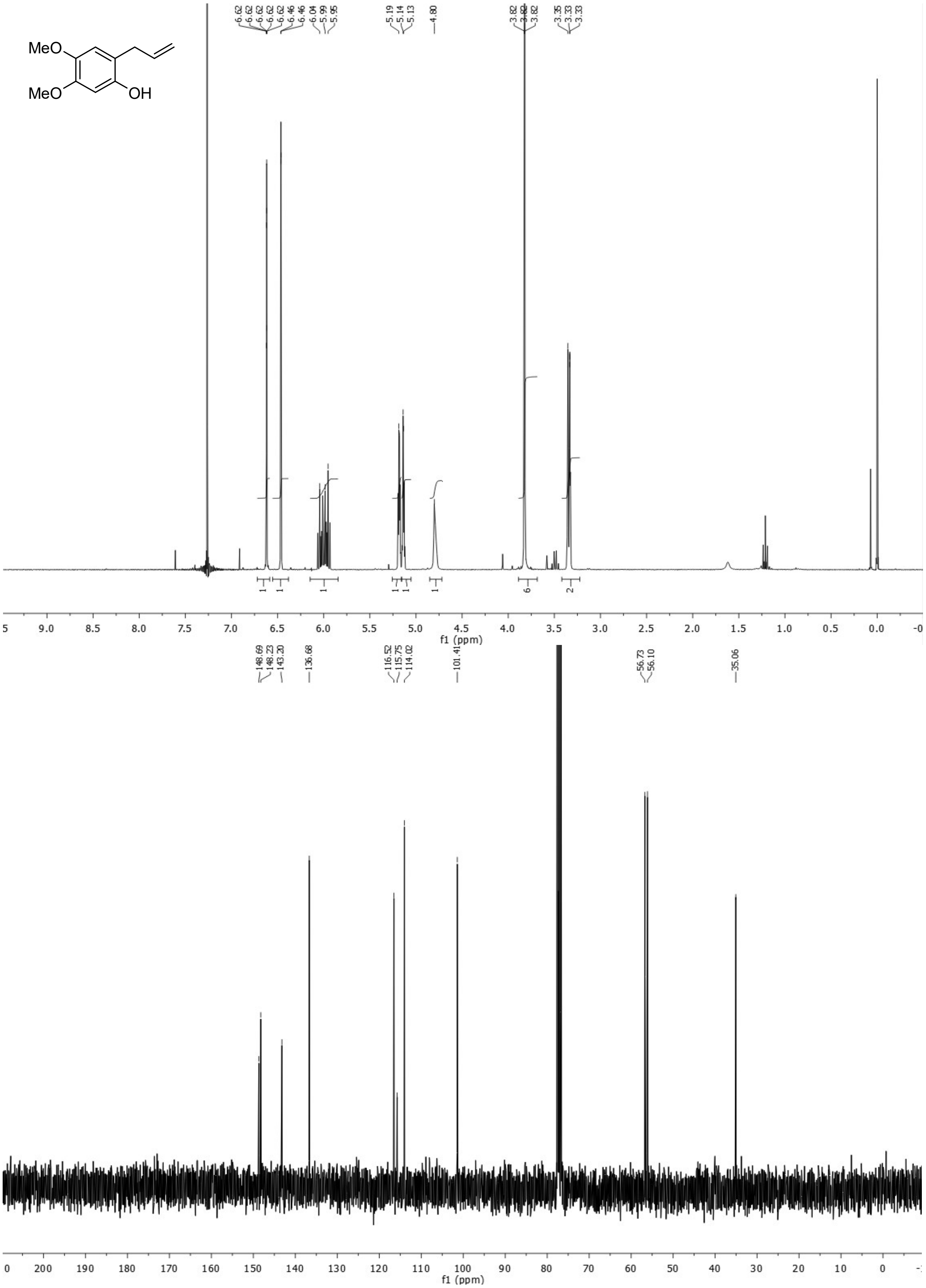

Fig. S53 ^{1}H NMR (300 MHz, $CDCl_3$) and ^{13}C NMR (75.5 MHz, $CDCl_3$) spectra of 2-allyl-4,5-dimethoxyphenol (**13**).

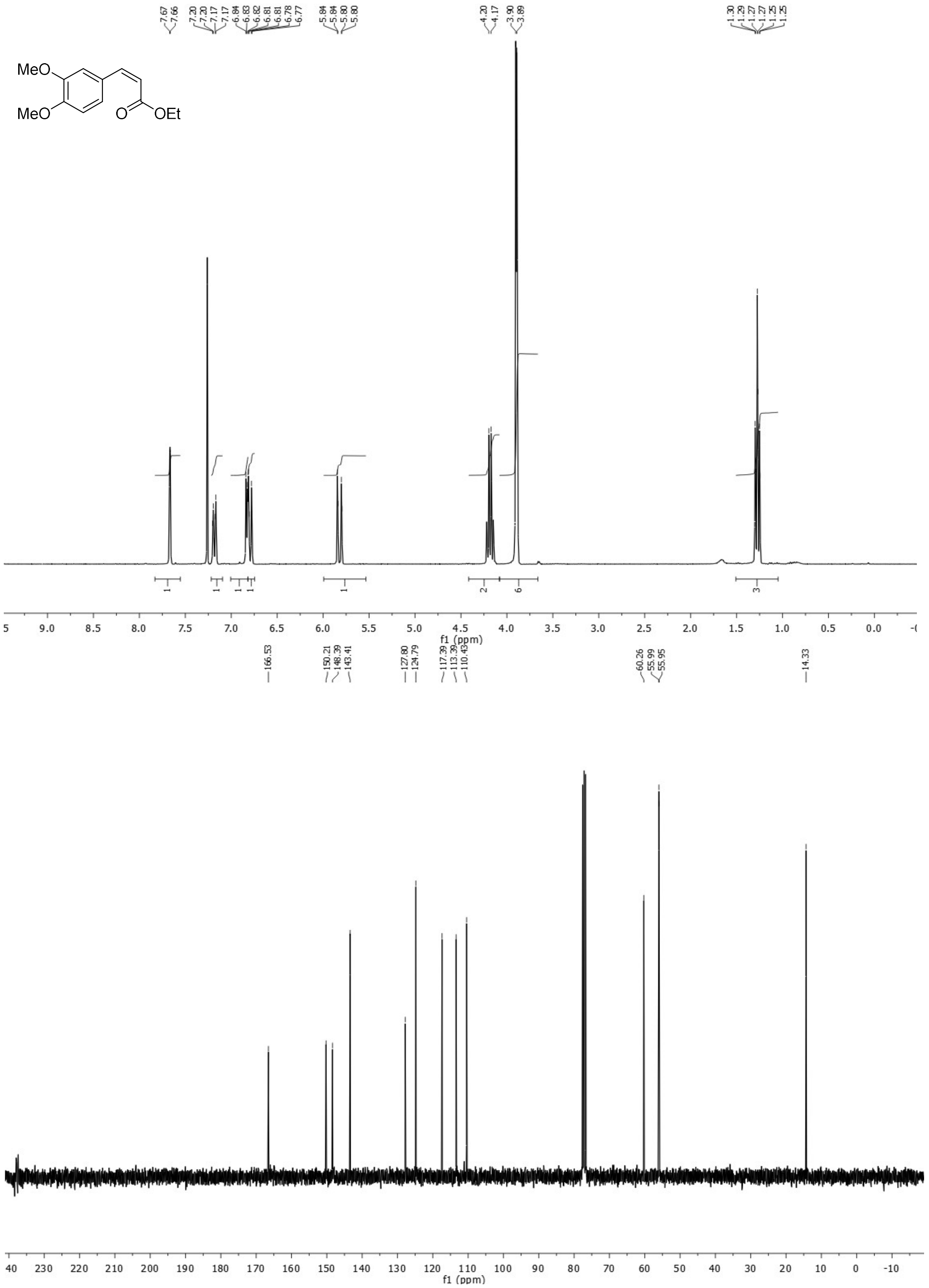

Fig. S54 ^{1}H NMR (300 MHz, $CDCl_3$) and ^{13}C NMR (75.5 MHz, $CDCl_3$) spectra of ethyl (*Z*)-3-(3,4-dimethoxyphenyl)acrylate (**S31**).

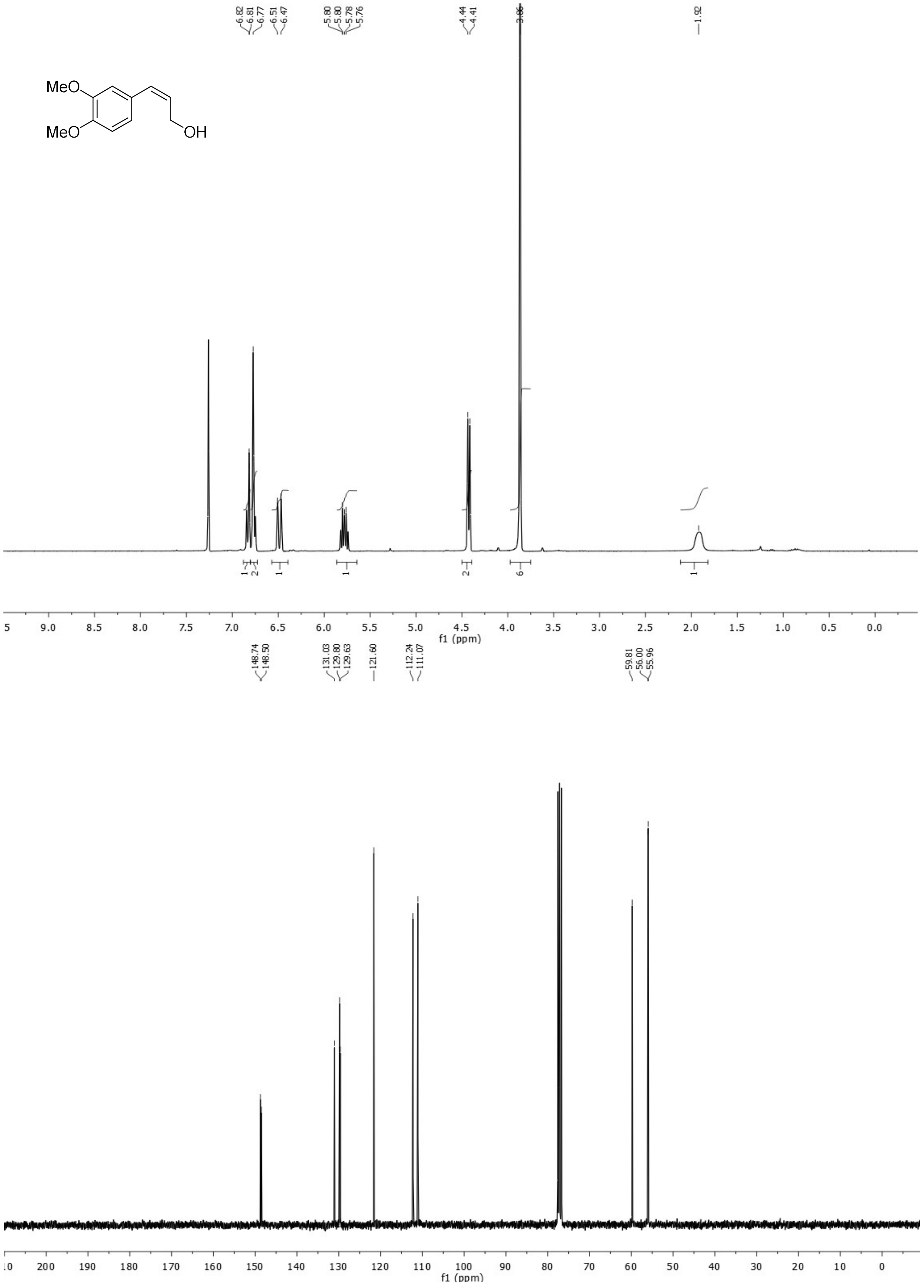

Fig. S55 ^{1}H NMR (300 MHz, $CDCl_3$) and ^{13}C NMR (75.5 MHz, $CDCl_3$) spectra of (*Z*)-3-(3,4-dimethoxyphenyl)prop-2-en-1-ol (**14**).

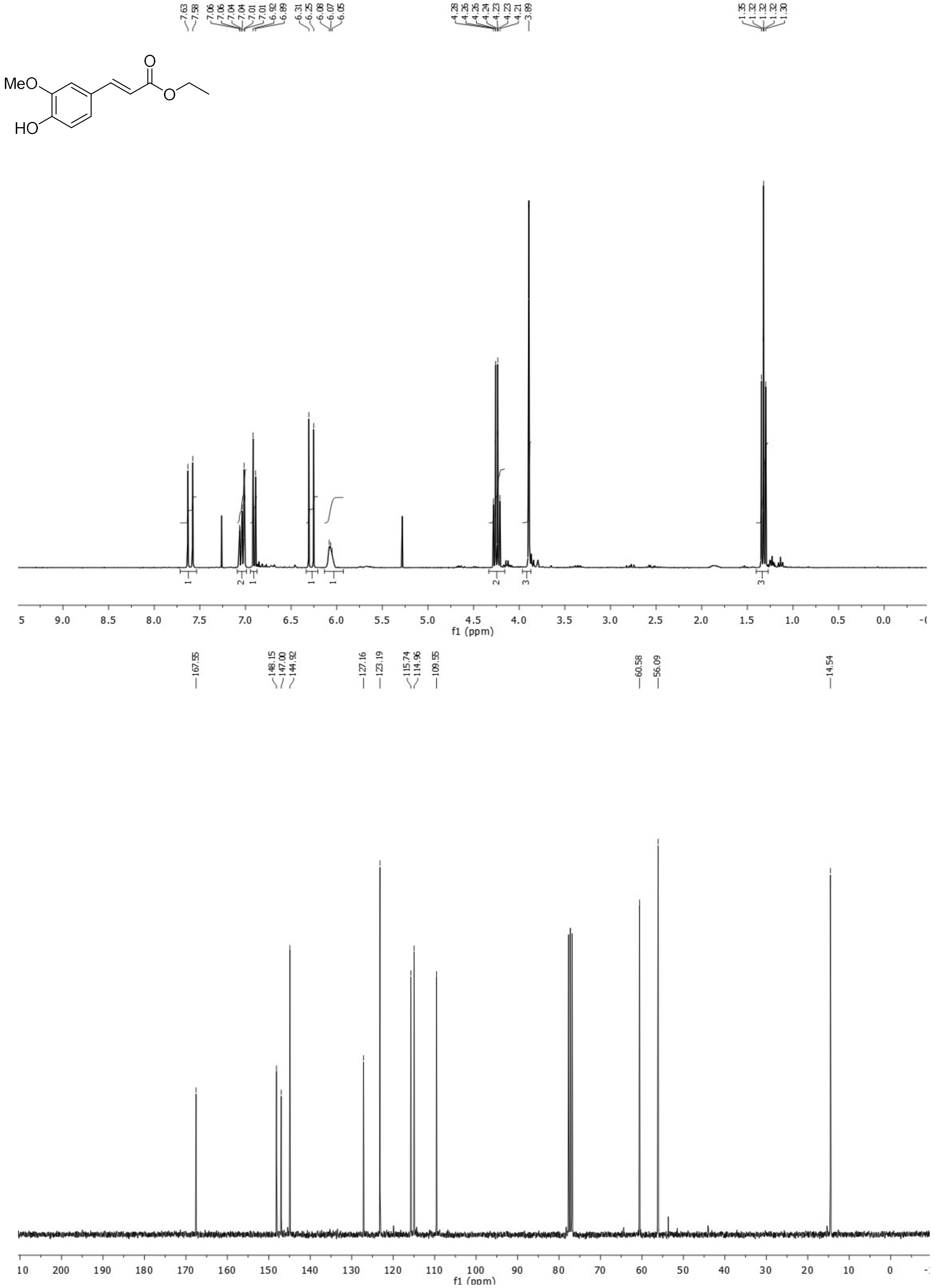

Fig. S56 ^{1}H NMR (300 MHz, $CDCl_3$) and ^{13}C NMR (75.5 MHz, $CDCl_3$) spectra of (*E*)-3-(4-hydroxy-3-methoxyphenyl)acrylate (**S33**).

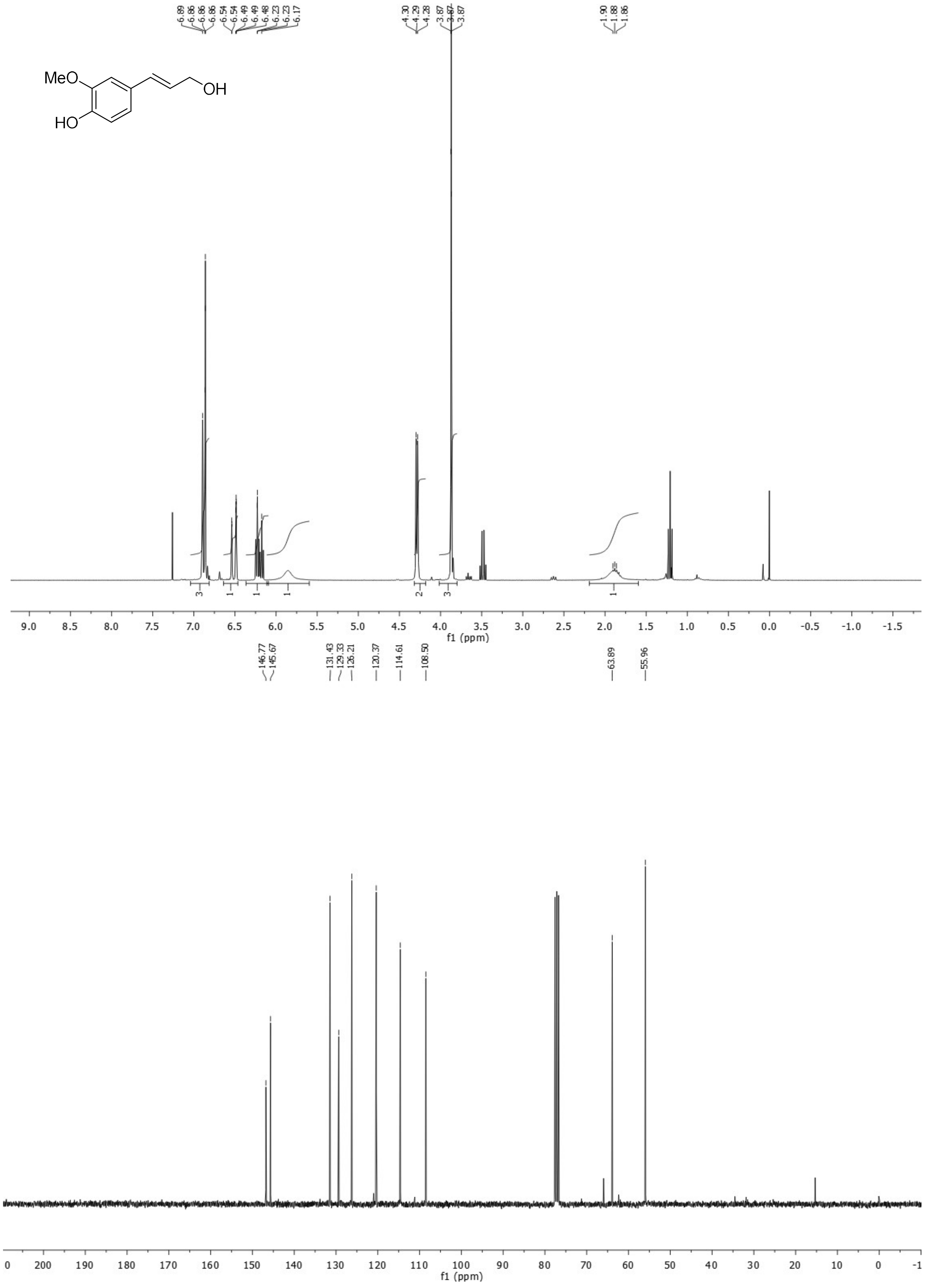

Fig. S57 ^{1}H NMR (300 MHz, $CDCl_3$) and ^{13}C NMR (75.5 MHz, $CDCl_3$) spectra of (*E*)-4-(3-hydroxyprop-1-en-1-yl)-2-methoxyphenol (**15**).

6 References

[1] Perkins VM, Fletcher TM, Kitching W, Drew RAI, Moore JC (1990) Chemical studies of rectal gland secretions of some species of*Bactrocera dorsalis* complex of fruit flies (diptera: Tephritidae). *J Chem Ecol* 16:2475–2487. https://doi.org/10.1007/BF01017470

[2] Meyers AI, Yamamoto Y, Mihelich ED, Bell RA (1980) Asymmetric synthesis of 2-Substitted Butyrolactones and Valerolactones. *J Org Chem* 45:2792–2796. https://doi.org/10.1021/jo01302a009

[3] Géant PY, Grenet E, Martínez J, Salom-Roig XJ (2016) Stereoselective divergent synthesis of 1,2-aminoalcohol-containing heterocycles from a common chiral nonracemic building block. *Tetrahedron Asymmetry* 27:22–30. https://doi.org/10.1016/j.tetasy.2015.11.008

[4] Schaus SE, Brandes BD, Larrow JF, Tokunaga M, Hansen KB, Gould AE, Furrow ME, Jacobsen EN (2002) Highly Selective Hydrolytic Kinetic Resolution of Terminal Epoxides Catalyzed by Chiral (salen)Co^{III} Complexes. Practical Synthesis of Enantioenriched Terminal Epoxides and 1,2-Diols. *J Am Chem Soc* 124:1307–1315. https://doi.org/10.1021/ja016737l

[5] Nicoli A, Haag F, Marcinek P, He R, Kreißl J, Stein J, Marchetto A, Dunkel A, Hofmann T, Krautwurst D, Pizio AD (2023) Modeling of odorant–receptor interactions. *J Chem Inf Model* 63:2014–2029. https://doi.org/10.1021/acs.jcim.2c00752

[6] Nabi A, Atmuri NDP, Arnold D, Saadati F, Tran H, Adak T, Dake GR, Ciufolini MA (2024) Claisen Self-Condensation of Lactones in the Synthesis of Ionizable Lipids. *J Org Chem* 89:12775–12778. https://doi.org/10.1021/acs.joc.4c01193

[7] Milan M, Carboni G, Salamone M, Costas M, Bietti M (2017) Tuning Selectivity in Aliphatic C–H Bond Oxidation of *N*-Alkylamides and Phthalimides Catalyzed by Manganese Complexes. *ACS Catal* 7:5903–5911. https://doi.org/10.1021/acscatal.7b02151

[8] Löbermann F, Weisheit L, Trauner D (2013) Intramolecular Vinyl Quinone Diels–Alder Reactions: Asymmetric Entry to the Cordiachrome Core and Synthesis of (−)-Isoglaziovianol. *Org Lett* 15:4324–4326. https://doi.org/10.1021/ol401787n

[9] Khan A, Khan S, Khan L, Zhao C, Mao Y, Chen Y, Zhang JY (2017) Enantioselective Construction of Tertiary C–O Bond via Allylic Substitution of Vinylethylene Carbonates with Water and Alcohols. *J Am Chem Soc* 139:10733–10741. https://doi.org/10.1021/jacs.7b04759

[10] Kudashev A, Baudoin O (2021) Site-selective Pd-catalyzed C(sp^3)–H arylation of heteroaromatic ketones. *Chem Eur J* 27:17688–17694. https://doi.org/10.1002/chem.202103467

[11] Davis CE, Bailey JL, Lockner JW, Coates RM (2003) Regio- and Stereoselectivity of Diethylaluminum Azide Opening of Trisubstituted Epoxides and Conversion of the 3° Azidohydrin Adducts to Isoprenoid Aziridines. *J Org Chem* 68:75–82. https://doi.org/10.1021/jo026506c

[12] Gally C, Nestl MB, Hauer B (2015) Engineering Rieske Non-Heme Iron Oxygenases for the Asymmetric Dihydroxylation of Alkenes. *Angew Chem Int Ed* 54:12952–12956. https://doi.org/10.1002/anie.201506527

[13] Chen X, Yao W, Zheng H, Wang H, Zhu D-Y, Wang S-H (2023) Enantiocontrolled total synthesis of (−)-retigeranic acid A. *J Am Chem Soc* 145:13549–13555. https://doi.org/10.1021/jacs.3c04850

[14] Stambaský J, Malkov A V, Kočovský P (2008) Synthesis of enantiopure 1-arylprop-2-en-1-ols and their *tert*-butyl carbonates. *J Org Chem* 73:9148–9150. https://doi.org/10.1021/jo801874r

[15] Häußler D, Gütschow M (2015) Synthesis of a Fluorescent-Labeled Bisbenzamidine Containing the Central (6,7-Dimethoxy-4-coumaryl)Alanine Building Block. *Heteroat Chem* 26:367–373. https://doi.org/10.1002/hc.21269

[16] Benbow JW, Katoch-Rouse R (2001) A Biomimetic Approach to Dihydrobenzofuran Synthesis *J Org Chem* 66:4965–4972. https://doi.org/10.1021/jo000696e

[17] Janicki I, Kiełbasiński P (2020) Still–Gennari olefination and its application in organic synthesis. *Adv Synth Catal* 362:2552–2596. https://doi.org/10.1002/adsc.201901591

[18] Gornati D, Sinisi R, Bertuolo S, De Matteo M, Di Fabio R (2022) Efficient synthesis of a novel euchromatic histone methyl transferase 2 (G9a) inhibitor. *Results in Chemistry* 4:100654. https://doi.org/10.1016/j.rechem.2022.100654

[19] Nishida R, Tan KH, Fukami H (1988) *Cis*-3,4-dimethoxycinnamyl alcohol from the rectal glands of male Oriental fruit fly, *Dacus dorsalis. Chem Express* 3:207–210.

[20] Wang C, Qian C, Roman M, Glasser GW, Eske RA (2013) Surface-initiated dehydrogenative polymerization of monolignols: a quartz crystal microbalance with dissipation monitoring and atomic force microscopy study *Biomacromolecules* 14:3964–3972. https://doi.org/10.1021/bm401084h

[21] Gangar M, Ittuveetil A, Goyal S, Pal A, Harikrishnan M, Nair VA (2016) Anti selective glycolate aldol reactions of (*S*)-4-isopropyl-1-[(*R*)-1-phenylethyl]imidazolidin-2-one: application towards the asymmetric synthesis of 8-4′-oxyneolignans. *RSC Adv* 6:102116–102126. https://doi.org/10.1039/C6RA22026F
